# Optimizing prescription of resistance training for body composition, muscle strength, and physical performance in older adults with sarcopenia: a systematic review and meta-analysis

**DOI:** 10.1186/s11556-025-00399-2

**Published:** 2026-01-21

**Authors:** Zhiyuan Tan, Yang Jiang, Darren G Candow, Carlo Castagna, Xiaolong Wang, Huakun Zheng

**Affiliations:** 1 University of Belgrade, Faculty of Sport and Physical Education, Belgrade, Serbia; 2 Yunnan Normal University, School of Physical Education, Kunming, China; 3 University of Regina, Faculty of Kinesiology & Health Studies, Regina, Canada; 4 Department of Education and Sport Sciences, Pegaso Telematic University, Naples, Italy; 5 China University of Geosciences, School of Physical Education, Wuhan, China; 6 Shanghai University of Sport, School of Exercise And Health, Shanghai, China

**Keywords:** Aging population, Exercise therapy, Postural balance, Hand strength, Rehabilitation, Randomized controlled trials

## Abstract

**Objective:**

This systematic review and meta-analysis aimed to address key gaps in understanding the role of resistance training (RT) as an intervention to mitigate age-related sarcopenia. Specifically, it examined: (i) effects on body composition and physical performance; (ii) moderating influences of age and training intensity; and (iii) the presence of a dose–response relationship within the FITT–VP framework.

**Methods:**

A comprehensive search of multiple databases identified randomized controlled trials (RCTs) evaluating RT in older adults with sarcopenia. Data on body composition, muscle strength (MS), and functional performance were extracted. Moderator analyses assessed the impact of participant and intervention characteristics, and meta-regression was performed to explore dose–response patterns.

**Results:**

Twenty-five RCTs involving 1,302 participants were included. RT produced significant improvements in MS (ES = 0.71), lean mass (LM, [ES = 0.22]), fat mass (FM, [ES = − 0.17]), walking ability (WA, [ES = 0.41]), grip strength ([GS, [ES = 0.55]), muscle quality (MQ, [ES = 1.25]) (all *p <* 0.05), but this large effect size was based on only two studies and requires caution interpretation. Dose–response meta-regression revealed a significant non-linear relationship between total RT duration and functional gains, with optimal estimated cumulative volumes of ~ 2,716 min for WA.

**Conclusion:**

RT is a robust, evidence-based strategy for enhancing MS, functional performance, and body composition in sarcopenic older adults. Findings suggest approximate cumulative duration ranges (~ 1,043 min for MS and ~ 2,716 min for WA) that were associated with maximal gains in pooled analyses. These values should be interpreted as exploratory indicators supporting individualized programming within the FITT-VP framework. Clinicians and exercise practitioners should tailor intensity (60–80% 1RM), frequency, and progression to optimize adherence, effectiveness, and long-term functional outcomes in sarcopenia management.

**Supplementary Information:**

The online version contains supplementary material available at 10.1186/s11556-025-00399-2.

## Key points

1. Effectiveness of RT: this review confirms that resistance training is a robust and effective intervention for improving muscle mass, strength, and physical performance in older adults with sarcopenia.

2. Optimization via FITT-VP: by applying the FITT-VP framework, the study identified a dose–response relationship, providing practical thresholds for training volume to optimize specific outcomes such as muscle strength and walking ability.

3. Clinical and Practical Relevance: findings offer the first evidence-based blueprint for tailoring RT protocols, supporting the development of precise clinical guidelines and enabling practitioners to prescribe targeted, individualized interventions to enhance functional outcomes and quality of life.

**Supplementary Information:**

The online version contains supplementary material available at 10.1186/s11556-025-00399-2.

## Introduction

The global demographic shift toward an aging population has evolved from a projected scenario to an immediate public health challenge. According to the United Nations World Population Prospects (2022), the number of individuals aged 60 years and older is expected to reach 2.1 billion by 2050 [1]. This unprecedented rise has placed age-related conditions such as sarcopenia at the forefront of geriatric care. Sarcopenia, a progressive skeletal muscle disorder characterized by declines in muscle mass (MM), strength, and physical performance [2] is associated with frailty, falls and fractures [3], impaired mobility and respiratory capacity [4], cognitive decline [5], diminished quality of life [5], increased mortality [6], and limited physical function [7]. Its prevalence increases steeply with age, affecting roughly 10–16% of older adults globally [8] and up to 50% of adults aged 80 years and above [9]. Given this increase and its socioeconomic consequences, prevention and treating sarcopenia is critical for maintaining independence and reducing care-related costs in aging societies.

Pharmacological therapies have yet to demonstrate consistent benefits for sarcopenia, with most agents offering limited efficacy and safety profiles [10, 11]. In contrast, exercise interventions, particularly resistance training (RT) have become the cornerstone of sarcopenia management, endorsed by the American College of Sports Medicine (ACSM) as a first-line treatment [12, 13]. RT, defined as planned, progressive exercise using external or body-weight resistance to elicit skeletal muscle adaptation [14], improves muscle quantity (MQ) and function through both neural and hypertrophic mechanisms [15]. Evidence indicates that RT promotes 30–60% gains in strength [16], increases in lean mass (LM) (5–10%) [16], and measurable improvements in postural balance (approximately 20–30%) [17], gait speed (GS) (0.08–0.2 m/s) [18, 19], and overall mobility (8–15% range of motion gain) [20]. Meta-analytic data confirm 1.1 kg increase in LM among sarcopenic older adults [16]. These gains are clinically meaningful: enhanced lower-limb strength reduces the likelihood of progressing to severe sarcopenia (OR = 0.65, 95% CI 0.52–0.81) [21], while higher GS correlates with reduced hospitalizations for heart failure (OR = 0.88, 95% CI 0.84–0.92) [22] and lower all-cause mortality [23]. Collectively, these outcomes position RT as a disease-modifying intervention capable of breaking the vicious cycle of muscle loss, functional decline, and disability.

Despite robust evidence supporting RT, key knowledge gaps persist regarding its long-term efficacy and optimal implementation. Meta-analyses by Peterson et al. (2011) [16] reported significant increase in LM (1.1 kg, 95% CI 1.0–1.2) and reductions in fat mass (FM) (1.0 kg, 95% CI 0.7–1.3) after 18–20 weeks, corroborated by Beckwée et al. (2019) [24] and Liao et al. (2017) [25]. Yet, inconsistencies remain concerning sustainability and intensity. Reginster et al. (2021) [26] identified diminished effects beyond 12 months, whereas Aagaard et al. (2010) [27] emphasized cumulative adaptations with prolonged RT. Sherrington et al. (2019) [28] demonstrated RT’s ability to enhance GS (0.08 m/s, 95% CI 0.04–0.12) and reduce falls by 23% (95% CI 15–31%), though substantial heterogeneity (I² > 70%) across trials remains [18, 29–31]. Such variations in moderate to high intensity (60–80% 1RM), frequency, and duration hinder the identification of optimal RT prescriptions for sarcopenic populations.

Although the FITT-VP framework (Frequency, Intensity, Time, Type, Volume, Progression) provides a systematic basis for exercise prescription [14, 32], previous studies have predominantly used it for descriptive categorization rather than quantitative optimization [33]. The lack of integrated analysis of its parameters, frequency, intensity, and overall volume interact to determine training outcomes has limited the translation of experimental evidence into precise clinical guidance [34, 35]. Recent consensus statements, including Bae et al. (2025) [36], highlight the urgent need for precision exercise prescription in older adults. However, quantitative thresholds linking RT characteristics to specific gains in muscle strength (MS) and mobility remain undefined.

Accordingly, this systematic review and meta-analysis aims to elucidate the effectiveness and optimization of RT in older adults with sarcopenia. Specifically, it (i) evaluates whether RT improves body composition and physical performance; (ii) examines whether its efficacy is moderated by individual or intervention-related factors (e.g., age, intensity, session, frequency); and (iii) quantifies the dose–response relationship within a FITT-VP-based analytical framework. By explicitly situating this analysis within a quantitative FITT-VP model, the study advances beyond descriptive evidence to identify empirically derived training volume thresholds for key functional outcomes, providing an evidence-based foundation for individualized RT optimization in sarcopenia management.

## Method

### Design

This study was conducted following the Preferred Reporting Items for Systematic Reviews and Meta-Analyses (PRISMA) guidelines [37] to ensure methodological transparency and rigor. The protocol for this systematic review has been prospectively registered on the PROSPERO platform (Registration ID: CRD420251061962), while the protocol registration was done after study retrieval and before data analysis.

### Search strategy

A comprehensive literature search was conducted across five databases (PubMed, Cochrane Library, Embase, SPORTDiscus, and Web of Science Core Collection) on April 7^th^−15^th^, 2025, with an updated search on September 19th, 2025 to verify completeness. The search strategy employed Boolean operators and was rigorously structured according to PICOS framework principles, combining free-text terms with Medical Subject Headings (MeSH) to ensure methodological thoroughness and precision. The core search syntax included: (“sarcopenia” OR “muscle loss” OR “muscle atrophy” OR “muscle weakness”) AND (“resistance training” OR “resistance exercise” OR “strength training” OR “weight training” OR “weight exercise” OR “elastic band” OR “progressive resistance” OR “grip strengthener” OR “1 RM” OR “TRX training”) AND (“aged” OR “elderly” OR “older adults” OR “seniors” OR “geriatric”). No date or sample size restrictions were applied during the search process. Additionally, we searched Google Scholar and ResearchGate and performed backward and forward snowballing via reference lists and citing articles to ensure the evidence base was as comprehensive as possible. The complete database search strategy is available in Supplementary Material S28.

### Selection process

All duplicate records were removed with EndNote 21 (Clarivate Analytics, Philadelphia, PA, USA). Subsequently, the remaining records were exported and independently screened by two authors (YJ and ZYT) based on predefined eligibility criteria. Initial screening was performed by reviewing titles and abstracts. Any discrepancies between reviewers were resolved through discussion with reference to the established criteria, with consensus achieved through mutual agreement. In cases where consensus could not be reached, the third author (HKZ) was consulted for arbitration. To enhance screening efficiency while maintaining rigorous oversight, we employed an AI-assisted approach using the ASReview tool (following the methodology described by Quan et al. [38]). ASReview applies active learning to prioritize relevant records, which has been validated to maintain sensitivity comparable to full manual screening in systematic reviews across multiple domains [38, 39]. To mitigate potential algorithmic bias and prevent the oversight of relevant records, we implemented a multi-layer supervision mechanism as recommended in recent AI-assisted systematic review frameworks [39]. Specifically, all records excluded by ASReview underwent full manual verification by an independent researcher (ZYT). This ensured that no potentially eligible study was erroneously discarded by the AI algorithm. Furthermore, to strengthen quality control, a second researcher (YJ) cross-checked a random sample of 20% of the AI-excluded records, with no discrepancies found, confirming the robustness of the AI-assisted screening process.

Finally, full-text articles were comprehensively evaluated by two independent researchers (ZYT and YJ) to determine final eligibility. Any disagreements during full-text screening were resolved using the same consensus protocol applied during title and abstract screening.

### Eligibility criteria

This systematic review adhered to strict PICOS criteria (Population, Intervention, Comparison, Outcome, Study design) criteria [40], including only randomized controlled trials (RCTs) published in English peer-reviewed journals involving adults aged ≥ 60 years with sarcopenia diagnosed according to recognized definitions (e.g., European Working Group on Sarcopenia in Older People [EWGSOP] [2], Asian Working Group for Sarcopenia [AWGS] [41], or Foundation for the National Institutes of Health [FNIH] criteria [42], or author defined cut offs). In some trials, participants were described as having sarcopenic obesity when sarcopenia definitions were applied in conjunction with obesity criteria based on Body Mass Index (BMI) or body-fat percentage (BFP) [43]. Given that sarcopenic obesity represents a distinct phenotype, these studies were flagged as a potential source of heterogeneity, and sensitivity analysis and subgroup analysis were used to investigate whether this phenotype significantly affected the main effect pooling; if significant effects were found, exclusion was performed [44].

The study population included both community-dwelling and institutionalized individuals with stable chronic comorbidities. Eligible interventions focused on RT as the primary modality (e.g., free weights, machines, and elastic bands), requiring explicit reporting of at least two of the following dose parameters: frequency, intensity, duration, or volume. Comparators included no exercise, non-RT interventions, or alternative RT regimens. The intervention measures of the experimental group were based on the control group with the addition of RT program. Primary outcomes included Body Composition Metrics (e.g., BMI, PBF, FM, Body Fat Mass [BFM], Body weight [BW], Skeletal Muscle Mass [SMM] and LM), Muscle Quantity Index (e.g., Skeletal Muscle Index [SMI]), Muscle Function Metrics (e.g., GS, MS and Muscle Quality [MQ]) and Physical Performance Metric (e.g., Walking Ability [WA]). We excluded animal studies, secondary analyses, conference abstracts, and grey literature to ensure methodological rigor.

### Data extraction

Two independent researchers (ZYT and YJ) systematically extracted data using a predefined standardized Excel template, capturing: (1) study characteristics (e.g., author, year), (2) participant characteristics (e.g., sex, age, weight, height, sample size and sarcopenic obesity), (3) RT intervention parameters (modality, intensity, frequency, duration, training sets and training repetition), (4) information on training adherence (attendance, session completion) and safety (adverse events or withdrawal reasons), and (5) body composition and physical performance outcomes. If adherence or safety data were missing, it was recorded as “not reported,” and adherence was expressed as the percentage of attended sessions out of the total prescribed. For graphical or inaccessible data, corresponding authors were contacted twice within 14 days; unresponsive cases were resolved using WebPlotDigitizer v4.8 (https://apps.automeris.io/wpd4/), a validated high-accuracy tool [45]. This rigorous approach ensured comprehensive and reproducible data collection in accordance with PRISMA guidelines [46].

### Data conversion

We systematically extracted mean values, standard deviations (SDs) and sample sizes from primary studies to calculate pre-post intervention differences. When only confidence intervals or standard errors (SEs) were reported, we converted them according to the Cochrane Handbook [40]. Given that correlation coefficients (r) between pre- and post-RT intervention measurements were rarely reported in included studies, we conservatively assumed *r* = 0.50 based on Cochrane recommendations [40]. To account for potential bias from small sample sizes, which were typical among eligible studies, we applied Hedges and Olkin’s g correction for effect size calculations [47]. The following conversion formulas were used:

$$\mathrm{SD}_\mathrm{pooled}=\sqrt{\frac{\left(\left(\mathrm{n}_1-1\right)\times\mathrm{SD}^2_1+\left(\mathrm{n}_2-1\right)\times\mathrm{SD}^2_2\right)}{\left(\mathrm{n}_1+\mathrm{n}_2-2\right)}}$$

$$\begin {aligned}\text{Effect Size}&=\frac{\left(\mathrm{Mean}_\mathrm{post}-\mathrm{Mean}_\mathrm{pre}\right)}{\mathrm{SD}_\mathrm{pooled}}\\&\times\left(1-\frac{3}{4\left(n_1+n_2-2\right)-1}\right)\end {aligned}$$

The effect sizes (ES) were calculated using the following parameters: n₁ and n₂ represent the sample sizes of the control and experimental groups at baseline and post-intervention, respectively; SD₁ and SD₂ denote the standard deviations of the control and experimental groups at baseline and post-intervention; and Meanₚ_r_ₑ and Meanₚₒₛₜ indicate the mean values at baseline and post-intervention. The magnitude of the ES was classified according to established clinical significance thresholds: < 0.2 (negligible), 0.2–0.5 (small), 0.5–0.8 (moderate), and > 0.8 (large) [48]. This standardized approach ensured consistent interpretation of intervention effects across studies while accounting for variability in baseline characteristics and outcome measurements.

### Assessment of methodological quality

Two independent investigators (ZYT and YJ) evaluated the methodological quality and reporting completeness of included studies using the TESTEX tool (specifically designed for exercise training research) and the Cochrane Risk of Bias 2.0 (RoB 2.0) tool. The TESTEX scale (total score: 15 points−5 for study quality and 10 for reporting quality) assesses 12 core criteria, addressing limitations of traditional tools by eliminating redundant items (e.g., blinding) while incorporating critical exercise-specific standards such as training dosage, intensity adjustment and control group activity monitoring [49]. Risk of bias was assessed using the RoB 2 tool [50] across five domains: randomization process, deviations from intended interventions, missing outcome data, outcome measurement, and selective reporting. Each study was independently evaluated by two reviewers (ZYT & YJ), with disagreements resolved through discussion.

#### Quality of evidence assessment

Certainty of evidence was graded using the GRADE framework [51], considering study limitations, consistency, directness, precision, and publication bias. This approach ensures a systematic and rigorous appraisal of both methodological quality and the reliability of findings [52].

Outcomes assessed included: Body composition indices: BMI, PBF, FM, SMM; Muscle quantity: SMI; Muscle function: GS, overall MS, and MQ; Physical performance: WA. Each outcome began as having “high” quality of evidence (reflecting the randomized design) and was subject to potential downgrading based on five domains: (1) risk of bias, (2) inconsistency (I² heterogeneity), (3) indirectness (variations in PICOS elements), (4) imprecision (wide confidence intervals), and (5) publications bias (funnel plot asymmetry or Egger’s test).

### Statistical analysis

#### Meta-analysis framework

All data analyses were conducted according to the recommendations of the Cochrane Handbook for Systematic Reviews of Interventions [53] and Hedges & Olkin’s Statistical Methods for Meta-Analysis [47]. Effect sizes (standardized mean differences, SMDs) and their 95% confidence intervals were calculated. A random-effects model was adopted to account for variability across studies [54]. Statistical heterogeneity was quantified by the I² statistic, with 25%, 50%, and 75% indicating low, moderate, and high heterogeneity, respectively [53, 55].

Exercise intensity for RT was categorized into low (< 50% 1RM), moderate (50–75% 1RM), and high (> 75% 1RM) levels based on the ACSM Guidelines for Exercise Testing and Prescription (2021) and previous RT meta-analyses in older adults [32, 34, 56]. This criterion provides a physiologically meaningful and reproducible framework for intensity classification, ensuring transparency and comparability.

Dose–response relationships were further modeled using fractional-polynomial regression following Hedges & Olkin [47, 57]. A non-linear meta-regression model was applied to examine potential dose–response patterns between cumulative RT volume and outcomes of interest. Because this analysis used aggregated trial-level data, it represents an exploratory approach that can reveal associative trends but cannot establish causal relationships or precise clinical thresholds. Publication bias was examined via Egger’s regression test and visual funnel-plot inspection; a p-value < 0.05 indicated potential bias [58].

#### Moderators analysis

Potential sources of heterogeneity and moderating factors (e.g., baseline participant characteristics and training protocols) were analyzed, with categorical variables examined via subgroup analysis and continuous variables via meta-regression [59]. Specifically, participants’ age and BMI were included in the meta-regression analysis.

Meta-regression analyses were performed only for outcomes that had sufficient data across studies (≥ 10 effect sizes with distinct training durations) [60]. Specifically, analyses were feasible for MS and WA. Other outcomes (e.g., LM, FM, and MQ) were excluded due to insufficient and highly heterogeneous data, which precluded reliable estimation.

#### Training dose determination and model fitting

Exercise volume was calculated according to the ACSM guidelines as session duration × frequency × cycle (weeks) [32]. Cumulative training volume (CTV) was selected as the primary dose metric, representing total accumulated duration of RT exposure (session time × weekly frequency × intervention weeks). This metric provides an integrated measure of workload across diverse protocols and reflects the fundamental determinants of neuromuscular adaptation under progressive overload. Using CTV allows modeling of non-linear dose-response relationships between total training dose and clinical outcomes (MS, WA, and body-composition indices), aligning with the FITT-VP framework by quantitatively operationalizing its “Volume” and “Time” components [34].

Because a strictly linear increase in rehabilitation effectiveness with increasing total exercise volume is biologically implausible, and preliminary comparisons indicated a better fit for nonlinear than for linear specifications, we focused on modelling potential nonlinear dose–response patterns [61]. Excluding linear relationships, a nonlinear association between total exercise volume and improvement in sarcopenia-related outcomes was examined using a restricted cubic spline (RCS) framework, comparing models with 3, 4, and 5 knots and retaining the specification that provided the best overall fit [62]. For nonlinear models exhibiting a clear inverted U-shaped trend, the dose corresponding to the maximum effect (i.e., the point on the fitted curve at which the effect size was maximized and the 95% CI did not cross 0) was extracted. A piecewise linear regression model was then constructed to validate this cut-point, with the likelihood ratio test (LRT) used to calculate the chi-square statistic; a p-value < 0.05 confirmed the statistical significance of the cut-point [63].

#### Sensitivity analyses and publication bias

We adopted a three-stage hybrid strategy to diagnose potential publication bias: (1) a multilevel meta-regression–based Egger’s regression test; (2) nonparametric Trim-and-Fill imputation under a random-effects framework; and (3) contour-enhanced funnel plot inspection combined with Egger’s regression testing (asymmetry significance threshold: *p* > 0.05) [58, 64, 65]. Sensitivity analyses were conducted using cluster-robust variance estimation with small-sample correction to account for within-study dependence and sampling variability. Systematic variation of key parameters was used to assess the stability of model coefficients across clinically relevant ranges, with model recalibration triggered when perturbations altered effect direction or statistical significance (*p* < 0.05). When results remained invariant across parameter ranges, the original estimates were retained [66].

## Results

### Literature search results

**Fig. 1:**
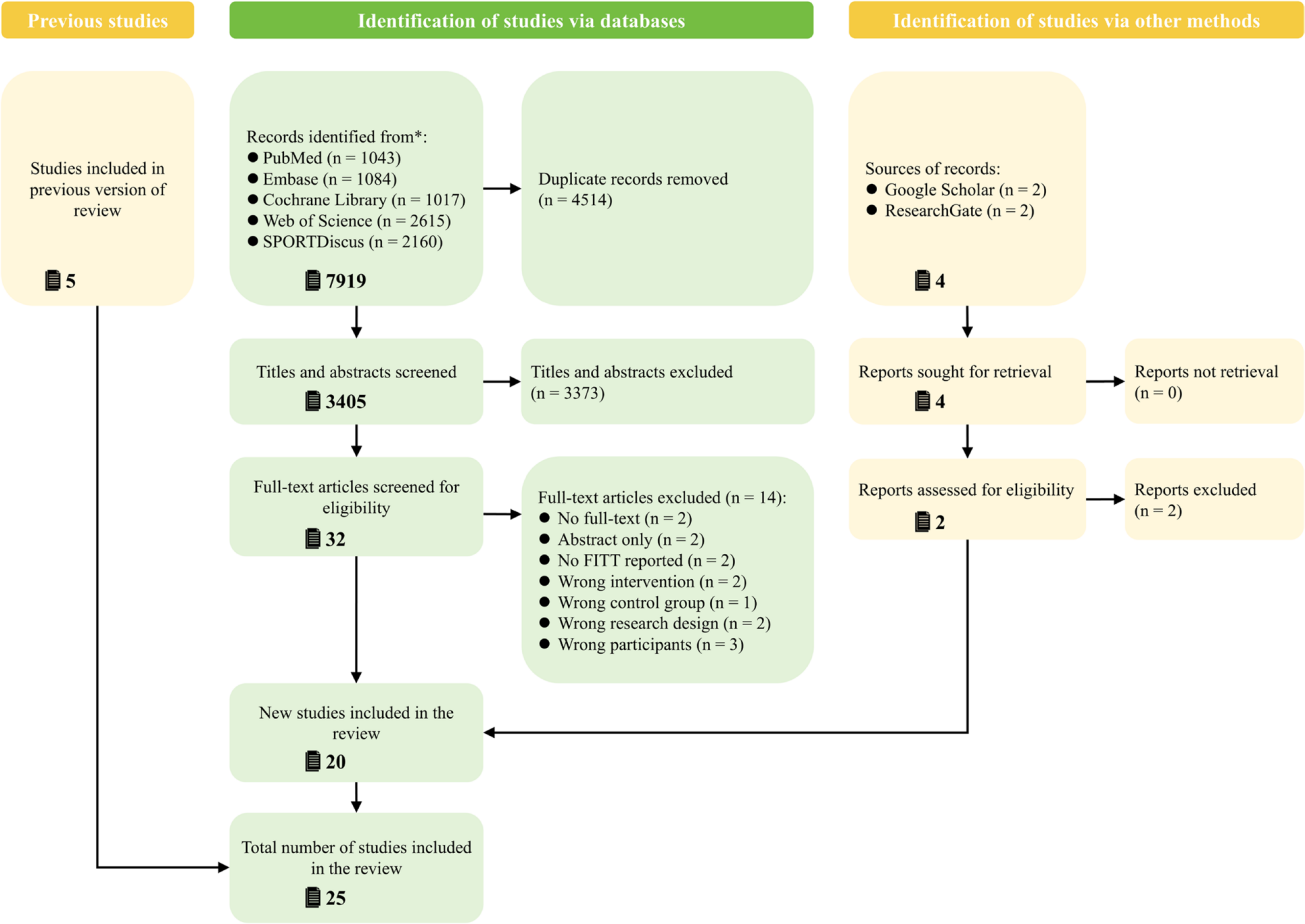
PRISMA flow diagram illustrating study selection and screening process

The PRISMA flow diagram for the study selection process is presented in Fig. 1. Literature searches in 5 electronic databases (PubMed, Embase, Cochrane Library, Web of Science, and SPORTDiscus) initially yielded 7,919 publications. After removal of 4,514 duplicates, 3,405 unique records remained for title and abstract screening. Following this screening, 3,373 records were excluded as not meeting the predefined inclusion criteria. The full texts of 32 articles were assessed for eligibility, of which 14 were excluded for various reasons (e.g., unavailable full text, abstract only, irrelevant intervention, inappropriate control, unsuitable design, or non-eligible participants). Ultimately, 18 studies from the database search met the inclusion criteria. In addition, other sources (Google Scholar and ResearchGate) yielded 4 additional reports, of which 2 met the eligibility criteria after full-text assessment. Combining these 20 new studies with 5 studies retained from the previous version, a total of 25 studies were included in the final meta-analysis.

### Characteristics of included studies

The meta-analysis included 25 RCTs comprising a total of 1,302 patients living with sarcopenia. Individual study sample sizes varied from 7 to 36 participants, with ages spanning 60.4 to 87.1 years, BMI ranging from 18.96 to 31.4 kg/m², and cohorts consisting of either single-sex or mixed-sex populations. Geographically, the studies represented diverse ethnic groups: 11 studies involved Chinese participants, 4 focused on Japanese populations, 2 examined Spanish, German, and Brazilian cohorts, respectively, while single studies were conducted in Korean, Iranian, Swedish, and Italian populations, respectively.

Notably, Among the 25 included RCTs diagnostic standards for sarcopenia varied (EWGSOP, *n* = 9; AWGS, *n* = 10; FNIH, *n* = 2; author defined cut offs, *n* = 4), with the latter (author-defined cutoffs) tailored to their specific study populations. Additionally, 6 trials explicitly enrolled sarcopenic obese participants and were analysed separately in sensitivity testing to account for the unique clinical characteristics of this subgroup.

Among the 25 included RCTs, 25 reported explicit adherence data. Mean training attendance ranged from 64.5% to 100%, indicating generally high compliance. Studies incorporating supervision or progressive elastic band protocols often reached adherence ≥ 97.6%. Regarding safety, no major adverser events (e.g., falls, fractures, cardiovascular) were documented. Several studies noted mild, transient muscle soreness and fatigue that resolved spontaneously. A detailed summary appears in Supplementary Material S29.

All interventions implemented RT-based protocols, with exercise modalities including weight training, kettlebell training, elastic band training, body-weight training, and chair MS training. Detailed participant characteristics and intervention protocols are summarized in Supplementary Material S2.

### Effects of RT on body composition

**Fig. 2:**
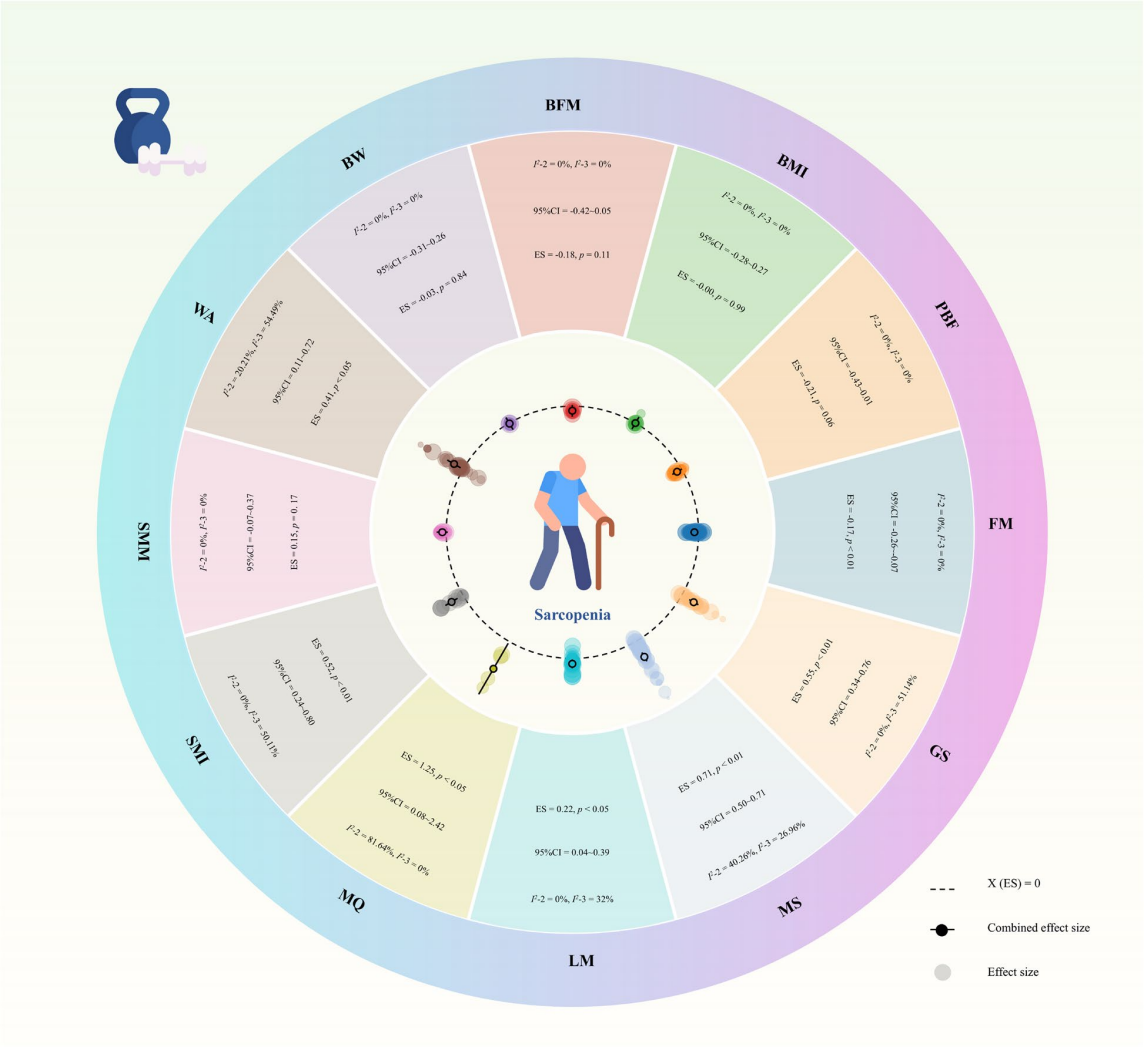
Summarizes the pooled effect sizes for each primary outcome, including body composition and muscle function indices. Note: BMI: Body Mass Index, PBF: percent body fat, FM: fat mass, BFM: body fat mass, BW: body weight, SMM: skeletal muscle mass, LM: lean mass, SMI: skeletal muscle index, GS: grip strength, MS: muscle strength, MQ: muscle quality, WA: walk ability

A meta-analysis of 25 studies evaluated the effects of RT on body composition compared to control conditions, revealing consistent improvements across multiple parameters. The findings demonstrate nuanced effects on various metrics, as detailed below: FM (16 studies; effect size [ES] =−0.17, 95% CI [–0.26,−0.07], *p* < 0.01), indicating a consistent benefit in decreasing adipose tissue. Moreover, RT significantly increased LM (11 studies; ES = 0.22, 95% CI [0.04, 0.39], *p* < 0.05), supporting its efficacy in promoting muscle hypertrophy. Results for other body composition variables (e.g., BMI, PBF, BFM, BW, and SMM) that did not reach statistical significance are presented in Fig. 2. In addition, sensitivity analysis results showed that the pooled results were stable, see Supplementary Materials S11.

### Effects of RT on muscle quantity index

Our analysis of 11 studies evaluated the impact of RT on the SMI, a key indicator of MQ relative to body size. The results demonstrated a significant positive effect of RT on SMI (ES = 0.52, 95% CI [0.24, 0.80], *p* < 0.01) (Fig. 2). In addition, sensitivity analysis results showed that the pooled results were stable, see Supplementary Materials S12.

### Effects of RT on muscle function

A meta-analysis of 19 studies evaluated the effects of RT on GS, revealing a significant improvement (ES = 0.55, 95% CI [0.34, 0.76], *p* < 0.01). Similarly, 21 studies assessed RT’s impact on overall MS, demonstrating a robust positive effect (ES = 0.71, 95% CI [0.50, 0.71], *p* < 0.01). Additionally, 2 studies examined RT’s influence on MQ, showing a significant enhancement (ES = 1.25, 95% CI [0.08, 2.42], *p* < 0.05) (Fig. 2). In addition, sensitivity analysis results showed that the pooled results were stable, see Supplementary Materials S13.

### Effects of RT on physical performance

The pooled analysis of 16 studies evaluated the effects of RT on WA, a key indicator of physical performance. The results demonstrated a significant improvement in WA following RT (ES = 0.41, 95% CI [0.11, 0.72], *p* < 0.05) (Fig. 2). In addition, sensitivity analysis results showed that the pooled results were stable, see Supplementary Materials S14.

### RT dose-response effects

**Fig. 3:**
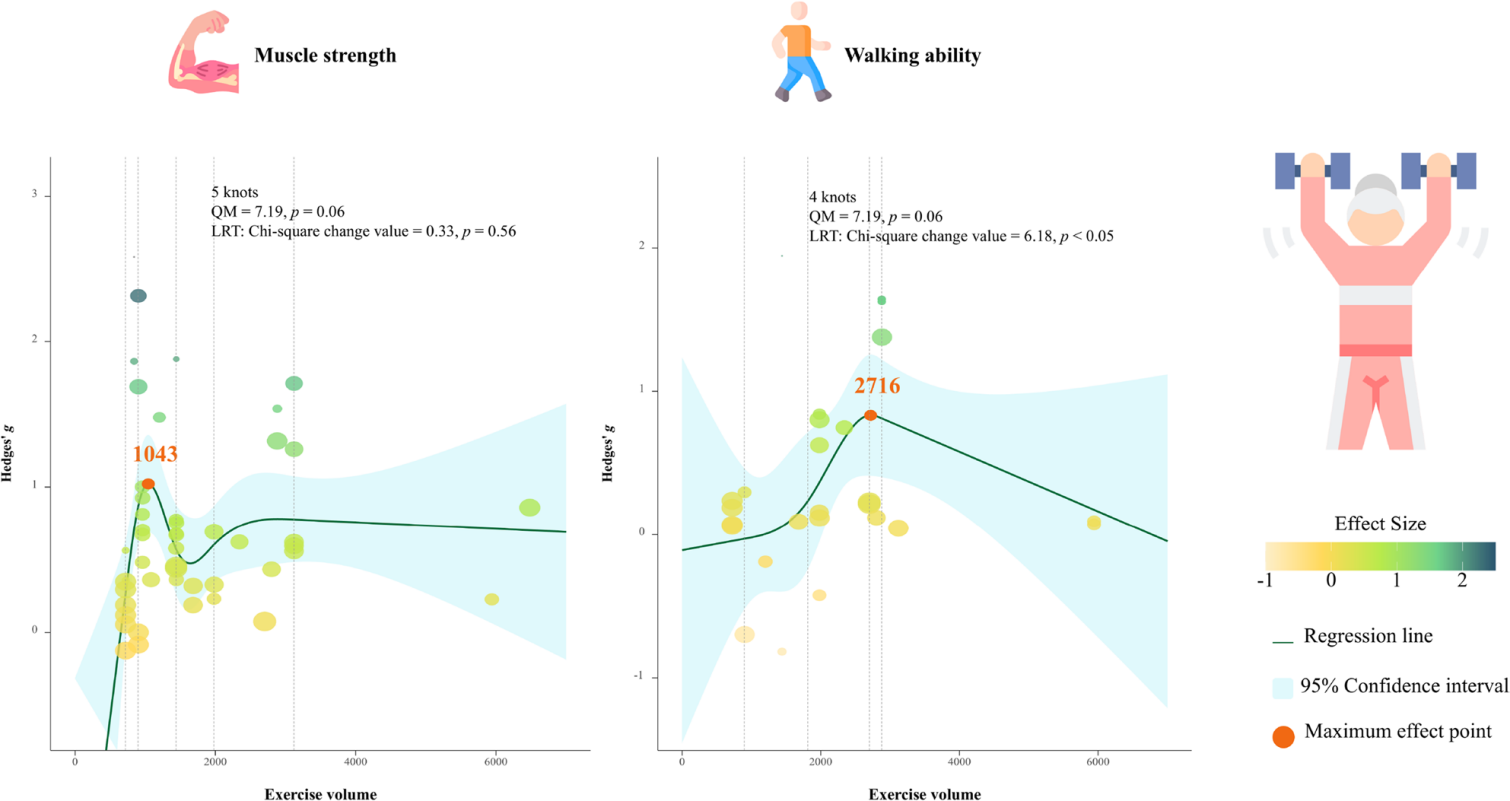
Presents the dose-response curves for muscle strength and walking ability derived from the non-linear meta regression analysis. Note: QM: Q statistic for Moderators, LRT: likelihood ratio test. Volume expressed as accumulated training time (minutes). For clinical interpretation, thresholds correspond approximately to moderate-intensity RT programs (60–80% 1RM, 2–3 sessions/week, 2–3 sets of 8–12 repetitions) over 8–12 weeks

A three-level meta-analysis integrating data from multiple randomized trials demonstrated that RT significantly improved MS (ES = 0.71, 95% CI [0.50, 0.71], *p* < 0.01). Sensitivity analyses confirmed the robustness of these pooled results (Supplementary Material S13). To further explore non-linear volume patterns, restricted cubic spline (RCS) meta-regression was conducted on aggregated study-level data. For MS, the curve suggested an apparent peak at approximately 1,043 min of total training volume, though this trend did not reach statistical significance (LRT χ² = 0.33, *p* = 0.56); thus, should be considered a potential inflection point rather than a definitive physiological threshold, consistent with the exploratory nature of the trend. For WA, a significant non-linear relationship was observed, indicating that an optimal cumulative RT volume of roughly 2,716 min could yield maximal functional improvement (LRT χ² = 6.18, *p* < 0.05), see Fig. 3. Dose–response modeling was limited to MS and WA because these outcomes provided sufficient data points for calculating cumulative training time, other endpoints (e.g., LM, FM, MQ) showed positive RT effects but lacked complete FITT-VP parameter reporting, preventing reliable non-linear estimation.

### Assessment of publication bias and certainty of evidence

Publication bias was assessed using funnel plot inspection and Egger’s regression test for all primary and secondary outcomes. Among the indicators, only GS (*p* = 0.0406), MS (*p* < 0.01), and WA (*p* = 0.011) demonstrated potential small-study effects, while the remaining outcomes showed *p*-values > 0.05, suggesting minimal risk of bias overall (see Supplementary Material S27).

Minor asymmetry observed in the body composition analyses may reflect small-study effects rather than true publication bias, yet this possibility cannot be excluded. The presence of potential bias was considered in the GRADE certainty appraisal, with body composition outcomes downgraded by one level for “risk of publication bias.”

The GRADE assessment indicated that, for all outcomes, the risks of bias, inconsistency and indirectness were judged as not serious, and no additional concerns were identified. However, imprecision was rated as serious for several key functional outcomes, including GS, overall MS, MQ, SMI, and WA, resulting in low to very low certainty of evidence for these domains. In contrast, outcomes related to body composition including LM, SMM, BW, BFM, BMI, BFP and total FM showed no serious limitations across any domain, and were therefore graded as having moderate certainty of evidence. Overall, these findings suggest that the evidence is more robust for body composition changes, whereas conclusions regarding improvements in muscle function and physical performance should be interpreted with greater caution due to imprecision and lower certainty.

Final summary ratings (“high,” “moderate,” “low,” or “very low”) and the justification for any downgrading are presented in Supplementary Material S26. This systematic grading ensures transparent evaluation of both the methodological rigor and the reliability of synthesized evidence.

### Assessment of methodological quality and level of evidence

The methodological quality and risk of bias of the 25 included studies were assessed using the TESTEX scale and Cochrane RoB 2.0 tool, with overall evidence-level assessment results are presented in Supplementary Material S23, S24. The studies exhibited robust methodological performance in key domains, including clearly defined eligibility criteria (100% fulfillment) and comparable baseline characteristics between groups (100% fulfillment). Randomization processes were consistently well-executed, with all studies rated as low risk in the RoB 2.0 randomization domain and TESTEX scores ranging from 5 to 10 (median = 7), indicating high reliability of the primary outcome data. Despite these strengths, several limitations were noted. Allocation concealment was universally absent (0% fulfillment in TESTEX), and assessor blinding was implemented in only 32% of studies. Furthermore, outcome measures were assessed in ≥ 85% of participants in just 44% of studies, with some studies showing elevated risks of missing outcome data. These deficiencies contributed to potential implementation bias and led to unclear overall risk-of-bias ratings in three studies [67–69] due to uncertainties in deviations from intended interventions, as per RoB 2.0. Nevertheless, 22 studies were classified as having low overall risk of bias.

## Discussion

This systematic review and meta-analysis comprehensively evaluated the effects of RT on body composition, muscle function, and physical performance in older adults with sarcopenia. The findings demonstrate that RT elicits significant improvements in MS, GS, MM, SMI, and WA, while producing a small but statistically significant reduction in FM. A large pooled effect was also observed for MQ (ES = 1.25). However, this estimate was derived from only two studies and should therefore be interpreted cautiously. Although the reduction in FM was statistically significant (ES = − 0.17), the magnitude of the effect is small and may not translate into clinically meaningful body fat changes. This modest response likely reflects that most RT interventions were not primarily designed to produce fat-loss, but rather to enhance MS and MQ. Future research with larger samples and standardized MQ measurement methods is warranted to validate this result.

A nonlinear dose–response relationship was identified, suggesting that optimal total training volumes approximately 2,716 min for WA may maximize functional gains. Although a nonlinear trend was observed for MS, the likelihood ratio test did not reach statistical significance (*p* = 0.56). Consequently, the identified 1,043 min threshold should be considered an approximate indicator of total RT exposure rather than a definitive prescription. These findings reinforce RT as a clinically robust, evidence-based modality for sarcopenia management and extend beyond general efficacy by providing actionable optimization parameters.

Unlike previous reviews that described FITT-VP components qualitatively, the present study quantifies dose–response thresholds, thereby operationalizing this framework for practical implementation. The recent meta-analysis provided valuable evidence on exercise interventions in older adults with sarcopenic obesity [70]. In contrast, our review integrates a broader sarcopenia spectrum including both sarcopenic and sarcopenic-obese populations, and employs quantitative meta-regression within the FITT-VP framework [32]. Together, these works offer complementary perspectives [34]. Kim et al. (2023) [70] established the general efficacy of RT, whereas the present study identifies dose-specific prescriptions and thresholds applicable to diverse sarcopenic phenotypes. The identified cumulative training volumes (~ 1,043 min for MS, ~ 2,716 min for WA) not only complement prior conceptual recommendations [71] but also transform descriptive associations into precise, evidence-based guidance for individualized RT prescription. By integrating the FITT-VP framework, this quantitative approach addresses key limitations of previous meta-analyses such as protocol heterogeneity and provides a methodological foundation for developing personalized, clinically translatable interventions to improve muscle function, mobility, and overall quality of life in aging populations [60].

### Effects of RT on body composition

One of the core findings of this systematic review and meta-analysis is that RT serves as an effective intervention to significantly improve body composition in older adults living with sarcopenia. Our findings indicate that RT not only effectively reduces FM but also improves BMI and total BW to some extent. This result re-affirms the critical role of RT in combating the pathophysiological processes of sarcopenia, specifically by optimizing body composition to slow or even reverse the vicious cycle of muscle loss and functional decline.

Our findings are in strong agreement with the meta-analysis by Peterson et al. (2011) [16], which reported significant lean body mass gains in older adults after structured RT programs, and with Strasser & Schobersberger (2011) [72], who documented statistically significant but small reductions in FM following resistance-based interventions in the elderly. Similar results were observed in the Cochrane review by Liu & Latham (2009) [73], though those authors did not identify optimal training volumes nor systematically account for moderator effects. In contrast, our study extends the literature by integrating the FITT-VP framework into a meta-regression model, thereby quantifying a nonlinear dose–response relationship and identifying optimal total training exposure (~ 1,043 min) for maximizing MS outcomes. While RT was associated with improved LM and FM indices, the analysis did not establish specific volume thresholds for these body composition metrics due to heterogeneous reporting across studies. This level of protocol specificity is largely absent in earlier research, representing a methodological and practical advance.

Theoretically, our findings refine current exercise prescription models by linking precise RT parameters with body composition outcomes in sarcopenic populations, moving beyond generalized recommendations. Practically, they provide clinicians with evidence-based, quantifiable guidelines for tailoring exercise interventions to maximize muscle gain and fat loss in older adults, ultimately enhancing functional independence and reducing healthcare burden.

### Effects of RT on muscle quantity index

A primary and crucial finding of this systematic review and meta-analysis is the significant improvement in muscle mass index among older adults living with sarcopenia following RT interventions. Our analysis confirms that RT serves as a potent stimulus for muscle hypertrophy, directly counteracting the defining pathological feature of sarcopenia, the age-related loss of MM.

Our results are consistent with the meta-analysis by Peterson et al. (2011) [74], which demonstrated significant gains in LM and appendicular SMM in older adults following RT, and with Liao et al. (2017) [25], who found that protein supplementation combined with RT further augments muscle mass indices in aging populations. Similarly, Shen et al. (2023) [19] reported RT as one of the most effective exercise modalities for increasing MQ in sarcopenia. However, unlike these studies, our analysis integrated the FITT-VP framework and meta-regression modeling to identify an optimal training volume threshold for Muscle Quantity Index improvements, highlighting a non-linear dose–response pattern that was not addressed in previous research.

Theoretically, these findings refine interventional models for sarcopenia by linking quantifiable training parameters to measurable hypertrophic outcomes. Practically, they offer clinicians and exercise professionals precise, evidence-based RT prescriptions to maximize MQ and mitigate disability risks in the elderly population.

### Effects of RT on muscle function

Beyond the foundational improvements in MM, this meta-analysis provides robust evidence that RT leads to substantial enhancements in muscle function, specifically in MS and WA, in older adults living with sarcopenia.

In sarcopenic older adults, RT enhances muscle function by inducing multifaceted adaptations at neural, muscular, and tendinous levels. Neurologically, RT increases motor unit recruitment, discharge rates, and intermuscular coordination, thereby improving the efficiency of voluntary force production [75]. At the muscular level, hypertrophy of fast-twitch fibers improves peak force and power output, while enhanced excitation contraction coupling boosts rate of force development [76]. RT also increases tendon stiffness and musculotendinous unit compliance, facilitating more effective force transmission to the skeleton, which is crucial for functional movements such as gait and chair-rise in elderly individuals [77]. Moreover, improved neuromuscular junction integrity and mitochondrial efficiency, as reported in aging muscle, further contribute to sustained contractile performance [78].

Our results align with the findings of Straight et al. (2016) [79], who observed significant gains in GS and chair-rise performance in older adults after 12 weeks of progressive RT, and the meta-analysis by Liu & Latham (2009) [73], which showed robust improvements in strength-related functional outcomes across various elderly cohorts. Similarly, Tieland et al. (2012) [80] confirmed that combined RT and adequate protein intake amplify muscle functionality by synergistically improving muscle power and coordination. However, our study expands these findings by identifying dose-dependent, nonlinear improvements in functional measures, such as WA, and by determining optimal training volumes (e.g., ~ 2,716 min, equivalent to approximately 45 h in total, which could be operationalized as three 60-minute sessions per week over 15 weeks) for WA using the FITT-VP framework, an element largely absent from prior work.

Theoretically, these results integrate neural and muscular adaptation mechanisms with quantitative exercise prescription, bridging a key gap between mechanistic understanding and clinical application. Practically, they offer practitioners a precise blueprint for designing RT interventions that maximize functional recovery, preserve independence, and reduce fall risk in sarcopenic elderly populations.

### Effects of RT on physical performance

In sarcopenic older adults, RT enhances physical performance through synergistic improvements in MS, neuromotor coordination, and metabolic capacity. Increased muscle cross-sectional area and contractile protein content improve absolute force production, enabling more efficient execution of daily tasks [74]. Neural adaptations, including improved motor unit recruitment patterns, reduced antagonist co‐activation, and enhanced synchronization, contribute to faster and more controlled movements [81]. At the metabolic level, RT promotes mitochondrial biogenesis and capillary density in active muscle fibers, delaying fatigue and improving endurance‐related functional tasks such as walking and stair climbing [82]. These effects directly translate into better scores in standardized functional tests, including GS, chair‐rise time, and the short physical performance battery (SPPB).

Our findings are in line with the network meta-analysis by Shen et al. (2023) [19], which ranked RT among the most effective exercise modalities for enhancing physical performance in sarcopenic elderly, particularly in improving GS and SPPB scores. Similarly, Liu & Latham (2009) [83] confirmed that progressive RT significantly improves performance‐based measures across older adult populations. Beaudart et al. (2017) [84] observed that gains in physical performance were mediated not only by MQ but also by improved MQ and neuromuscular efficiency, supporting our mechanistic model. However, unlike most previous studies, our analysis incorporated a dose–response perspective using the FITT‐VP framework, revealing non‐linear optimal thresholds of total RT time for maximal functional gains adding a practical prescription nuance largely missing from earlier literature.

Mechanistically, the beneficial effects of RT on sarcopenia-related outcomes can be explained by several complementary biological and physiological pathways. RT activates the mechanistic target of rapamycin (mTOR) signaling cascade, which promotes muscle protein synthesis and hypertrophy, especially in type II fibers that are highly susceptible to age-related atrophy [85, 86]. In parallel, RT suppresses myostatin, a key negative regulator of muscle growth [87] while increasing IGF-1 expression and stimulating satellite cell proliferation, all of which enhance regenerative capacity and tissue repair [88, 89]. Moreover, repetitive RT elicits neuromuscular adaptations that improve motor unit recruitment, synchronization, and junctional integrity, ultimately enhancing strength and functional performance [90]. This integration of anabolic and neural mechanisms may provide the biological basis for the dose–response thresholds observed in our meta-analysis, linking exercise volume and intensity to meaningful functional improvements [91].

Theoretically, these results integrate morphological, neural, and metabolic adaptations to explain physical performance enhancement in sarcopenic older adults. Practically, they provide precise and evidence-based RT protocols that can be implemented by clinicians and physical therapists to improve mobility, reduce fall risk, and promote independence in aging populations.

### RT dose-response effects

In sarcopenic older adults, the dose–response relationship between RT and improvements in MS, MQ, and physical performance appears to follow a non-linear, inverted-U pattern. This reflects the interplay between training-induced anabolic signaling such as mTORC1 activation, satellite-cell proliferation, and enhanced motor-unit recruitment, and individual recovery capacity [92, 93]. Moderate total training volumes (e.g., frequency × sets × repetitions × load) promote hypertrophy and neuromuscular adaptation, whereas volumes exceeding the adaptive threshold may impair recovery, elevate inflammation and cortisol, and attenuate anabolic pathways [34].

Exploratory aggregate-level meta-regression identified apparent inflection points, with cumulative RT volumes of approximately 1,043 min for MS and 2,716 min for WA being associated with the greatest observed improvements across included trials. These estimates should be regarded as descriptive indicators rather than strict clinical cut-offs, as they are influenced by heterogeneity in program intensity, volume, progression, and participant characteristics. Large-scale, standardized RCTs are needed to confirm these ranges and refine clinically applicable thresholds [60].

Consistent with the hormesis model, training benefits rise with dose until an optimal threshold is reached, then plateau or decline as fatigue accumulates [94]. Progressive overload implemented by gradual increases (about 2–10%) in load or volume when prescribed repetitions are comfortably achieved is essential for sustaining adaptation within the FITT-VP paradigm [32]. Individualizing progression models (linear, undulating, or autoregulatory) to match functional goals and recovery capacity can maximize adaptation, minimize fatigue and injury risk, and enhance long-term compliance in sarcopenic older adults [95].

### Publication bias and interpretation

Egger’s test revealed evidence of potential publication bias for some primary outcomes (GS, MS, and WA). Although trim-and-fill corrections indicated that the overall effect sizes remained in the same direction, the magnitude of improvement may have been slightly overestimated due to small-study effects and selective reporting of positive results. Although publication bias was detected for a subset of outcomes (GS, MS, WA), the direction of effects remained consistent after trim-and-fill correction. This suggests that the overall pattern of benefits is robust, yet the absolute effect sizes should be interpreted with caution. Therefore, these findings should be interpreted with appropriate caution when translating to clinical practice, and future large, pre-registered, multi-center trials are warranted to confirm these dose–response relationships with reduced risk of publication bias. Given the exploratory nature of spline-based meta-regression, the observed intensity–response pattern should be considered hypothesis-generating rather than confirmatory.

### Limitations and perspectives

Although this meta-analysis provides robust evidence that RT improves MS and WA in sarcopenic older adults, several methodological limitations warrant caution. The dose–response estimates (~ 1,043 min for MS; ~2,716 min for WA) were derived from aggregated study-level training durations, which may oversimplify the complex interplay among intensity, volume, frequency, and progression; thus, these values should be regarded as preliminary quantitative references rather than definitive clinical prescriptions. Substantial heterogeneity in RT protocols, participant characteristics, intervention lengths, and assessment methods may have influenced pooled estimates, while variability in exercise modalities and divergent diagnostic criteria for sarcopenia further limited subgroup analyses and introduced residual heterogeneity.

Reporting on adherence and adverse event monitoring was inconsistent across trials, preventing formal meta-analysis of these outcomes and restricting comprehensive evaluation of RT safety. Incomplete allocation concealment and limited blinding in several studies may have introduced bias, and inclusion of trials involving sarcopenic obesity, potentially characterized by distinct metabolic and inflammatory responses, adds to this complexity. Nevertheless, subgroup analyses indicated that the moderating effect of sarcopenic obesity was not significant across any outcome, although this population may differ from individuals with non-obese sarcopenia in important ways and therefore warrants greater attention in future research. Egger’s tests identified potential publication bias for GS (*p* = 0.0406), MS (*p* < 0.01), and WA (*p* = 0.011). Visual inspection of funnel plots (Supplementary Material S27) revealed slight asymmetry, suggesting possible small-study effects or selective reporting of positive outcomes. Trim-and-fill procedures indicated that corrected effect sizes remained in the same direction but were slightly reduced in magnitude, implying modest overestimation of benefits. Although statistical tests suggested limited overall publication bias for other outcomes, the small number of available RCTs may obscure asymmetry patterns, meaning publication bias cannot be completely ruled out. Although the three-level meta-analytic model captures complex data structures, the limited number of studies within each intensity category may restrict the precision of spline estimates. Therefore, our conclusions on dose–response trends should be viewed as preliminary. These considerations were factored into our GRADE appraisal, with specific outcomes downgraded for “risk of publication bias.”

Despite generally moderate-to-high methodological quality (TESTEX, RoB 2.0), these limitations underscore the need for adequately powered, standardized RCTs adopting uniform diagnostic criteria, transparent reporting of FITT–VP parameters, and systematic documentation of adherence, dropout reasons, and adverse events.

Future trials should prioritize pre-registered protocols, incorporate unpublished or ongoing studies where possible, and ensure consistent bias assessment to enhance robustness of pooled estimates, confirm dose–response relationships, and establish individualized, evidence-based RT prescriptions for sarcopenia management.

### Practical application

Based on pooled evidence and established guidelines [32, 96], optimal RT prescriptions for MS and WA in sarcopenic older adults can be defined as moderate to high intensity (60–80% 1RM), performed 2–3 times per week, with 2–3 sets of 8–12 repetitions for each major muscle group over 8–12 weeks. These parameters yield approximately 1,000–1,043 total minutes for MS improvement and ~ 2,716 min for WA enhancement. Expressing cumulative RT volume in minutes standardizes diverse protocols, enabling integration across trials.

High adherence rates and the absence of serious adverse events support the feasibility and safety of such programs, particularly under supervised conditions and within the prescribed intensity range. However, incomplete reporting of adverse events, compliance monitoring, and dropout causes in several studies limits comprehensive risk–benefit assessment. Future research should adopt standardized reporting of load, sets, frequency, progression, adherence, reasons for withdrawal, and safety outcomes to refine clinical precision and strengthen the evidence base.

This evidence provides a practical, FITT–VP–based blueprint for tailoring RT programs to individual functional goals, thereby improving adherence, maximizing effectiveness, and enhancing long-term quality of life among aging populations.

## Conclusions

This systematic review and meta-analysis confirms that RT is an effective intervention for older adults with sarcopenia. More importantly, it advances the field by moving beyond this general consensus to address the critical question of how to optimize RT for specific outcomes. While previous consensus statements and clinical guidelines have outlined broad exercise recommendations, they seldom define quantitative thresholds for dose–response optimization. Our meta-regression fills this gap by establishing empirical dose criteria around 1,043 min for MS and 2,716 min for WA, derived through nonlinear modeling within the FITT-VP framework. This quantification extends existing conceptual guidelines such as Bae et al. [36] into a measurable, implementable prescription model.

## Supplementary Information

Supplementary Material 1.

Supplementary Material 2.

## Data Availability

No datasets were generated or analysed during the current study.
