# Supplementary material for: Optimizing prescription of resistance training for body composition, muscle strength, and physical performance in older adults with sarcopenia: a systematic review and meta-analysis
Source: Eur Rev Aging Phys Act. 2026 Jan 21;23:8. doi: 10.1186/s11556-025-00399-2 (PMC12908291; doi:10.1186/s11556-025-00399-2)
Supplement: Supplementary file 2 — Supplementary Material 2. [file 11556_2025_399_MOESM2_ESM.docx]

**Supplementary materials**

**S1 PRISMA statement.**

| **Section/topic** | **#** | **Checklist item** | **Reported on page #** |
| --- | --- | --- | --- |
| **Optimizing Prescription of Resistance Training for Body Composition, Muscle Strength, and Physical Performance in Older Adults with Sarcopenia: A Systematic Review and Meta-Analysis** | | |  |
| Title | 1 | Identify the report as a systematic review, meta-analysis, or both. | p.1 |
| **ABSTRACT** | | |  |
| Structured summary | 2 | Provide a structured summary including, as applicable: background; objectives; data sources; study eligibility criteria, participants, and interventions; study appraisal and synthesis methods; results; limitations; conclusions and implications of key findings; systematic review registration number. | p.2 |
| **INTRODUCTION** | | |  |
| Rationale | 3 | Describe the rationale for the review in the context of what is already known. | p.3 |
| Objectives | 4 | Provide an explicit statement of questions being addressed with reference to participants, interventions, comparisons, outcomes, and study design (PICOS). | p.3-4 |
| **METHODS** | | |  |
| Protocol and registration | 5 | Indicate if a review protocol exists, if and where it can be accessed (e.g., Web address), and, if available, provide registration information including registration number. | p.5 |
| Eligibility criteria | 6 | Specify study characteristics (e.g., PICOS, length of follow-up) and report characteristics (e.g., years considered, language, publication status) used as criteria for eligibility, giving rationale. | p.5 |
| Information sources | 7 | Describe all information sources (e.g., databases with dates of coverage, contact with study authors to identify additional studies) in the search and date last searched. | p.5-6 |
| Search | 8 | Present full electronic search strategy for at least one database, including any limits used, such that it could be repeated. | p.5-6 |
| Study selection | 9 | State the process for selecting studies (i.e., screening, eligibility, included in systematic review, and, if applicable, included in the meta-analysis). | p.5–6 |
| Data collection process | 10 | Describe method of data extraction from reports (e.g., piloted forms, independently, in duplicate) and any processes for obtaining and confirming data from investigators. | p.9 |
| Data items | 11 | List and define all variables for which data were sought (e.g., PICOS, funding sources) and any assumptions and simplifications made. | p.9–10 |
| Risk of bias in individual studies | 12 | Describe methods used for assessing risk of bias of individual studies (including specification of whether this was done at the study or outcome level), and how this information is to be used in any data synthesis. | p.11 |
| Summary measures | 13 | State the principal summary measures (e.g., risk ratio, difference in means). | p.11 |
| Synthesis of results | 14 | Describe the methods of handling data and combining results of studies, if done, including measures of consistency (e.g., I^2^) for each meta-analysis. | p.12 |

Page 1 of 2

| **Section/topic** | **#** | **Checklist item** | **Reported on page #** |
| --- | --- | --- | --- |
| Risk of bias across studies | 15 | Specify any assessment of risk of bias that may affect the cumulative evidence (e.g., publication bias, selective reporting within studies). | p.12–13 |
| Additional analyses | 16 | Describe methods of additional analyses (e.g., sensitivity or subgroup analyses, meta-regression), if done, indicating which were pre-specified. | p.12–13 |
| **RESULTS** | | |  |
| Study selection | 17 | Give numbers of studies screened, assessed for eligibility, and included in the review, with reasons for exclusions at each stage, ideally with a flow diagram. | p.32 |
| Study characteristics | 18 | For each study, present characteristics for which data were extracted (e.g., study size, PICOS, follow-up period) and provide the citations. | p.14–15 |
| Risk of bias within studies | 19 | Present data on risk of bias of each study and, if available, any outcome level assessment (see item 12). | p.16 |
| Results of individual studies | 20 | For all outcomes considered (benefits or harms), present, for each study: (a) simple summary data for each intervention group (b) effect estimates and confidence intervals, ideally with a forest plot. | p.17–20 |
| Synthesis of results | 21 | Present results of each meta-analysis done, including confidence intervals and measures of consistency. | p.20–22 |
| Risk of bias across studies | 22 | Present results of any assessment of risk of bias across studies (see Item 15). | p.23 |
| Additional analysis | 23 | Give results of additional analyses, if done (e.g., sensitivity or subgroup analyses, meta-regression [see Item 16]). | p.24–25 |
| **DISCUSSION** | | |  |
| Summary of evidence | 24 | Summarize the main findings including the strength of evidence for each main outcome; consider their relevance to key groups (e.g., healthcare providers, users, and policy makers). | p.26 |
| Limitations | 25 | Discuss limitations at study and outcome level (e.g., risk of bias), and at review-level (e.g., incomplete retrieval of identified research, reporting bias). | p.27 |
| Conclusions | 26 | Provide a general interpretation of the results in the context of other evidence, and implications for future research. | p.28 |
| **FUNDING** | | |  |
| Funding | 27 | Describe sources of funding for the systematic review and other support (e.g., supply of data); role of funders for the systematic review. | p.29 |

*From:*  Moher D, Liberati A, Tetzlaff J, Altman DG, The PRISMA Group (2009). Preferred Reporting Items for Systematic Reviews and Meta-Analyses: The PRISMA Statement. PLoS Med 6(7): e1000097. doi:10.1371/journal.pmed1000097

For more information, visit: **[www.prisma-statement.org](http://www.prisma-statement.org.)**[.](http://www.prisma-statement.org.)

**S2 Basic characteristics of literature.**

| Study (year) | Country or region | Participants | Diagnostic criteria | Intervention Program | Exercise intensity | Exercise Frequency Session Per Week | Exercise Duration, Week | Exercise Session Duration, min | Outcome | Samplesize (n) | | Age (years) | BMI (kg/m^2^) |
| --- | --- | --- | --- | --- | --- | --- | --- | --- | --- | --- | --- | --- | --- |
|  |  |  |  |  |  |  |  |  |  | Male | Female |  |  |
| (Huang, Ku et al. 2017) | Taiwan | Sarcopenic obesity | AWGS | T-1: Weight training at 60–70% 1RM; 10 exercises targeting major muscle groups; progressive overload every 2 weeks; 3 sets of 8–12 reps. | 60-70% 1RM | 2 | 8 | 60 | Body composition: Skeletal muscle mass (kg), Body fat mass (kg), BMI (kg/m²), Percent body fat (%), Visceral fat area (cm²) Muscle strength: grip strength (kg), back extensor strength (kg), knee extensor strength (kg) Biomarker: serum IGF-1 (ng/mL) | 3 | 12 | 68.9 ± 4.4 | 28.3 ± 4.4 |
|  |  |  |  | T-2: Moderate intensity aerobic dance movements including stepping, knee lifts, squats, arm swings; 40-45 min actual training | Moderate intensity (not quantified) |  |  |  |  | 1 | 14 | 69.3 ± 3 | 26.8 ± 3.8 |
|  |  |  |  | T-3: resistance training once /week + aerobic training once /week (48h apart). | As per resistance training and aerobic training |  |  |  |  | 4 | 11 | 68.5 ± 2.7 | 27.2 ± 2.9 |
|  |  |  |  | C: Maintain usual lifestyle. | / |  |  |  |  | 2 | 13 | 68.6 ± 3.1 | 29 ± 3.9 |
| (Chen, Wu et al. 2018) | Taiwan | Sarcopenia | EWGSOP | T: 8-week kettlebell training, progressive resistance, 60 min/session | 60%-70% of 1RM | 2 | 8 | 60 | SMM (kg), ASM (kg), BFM (kg), VFA (cm²), grip strength (kg), back strength (kg), PEF (L/s), FVC (L), hs-CRP (mg/L), IL-6 (pg/mL), TNF-α (pg/mL) | 17 | | 66.7 ± 5.3 | / |
|  |  |  |  | C: No exercise training. | / |  |  |  |  | 16 | | 68.3 ± 2.8 | / |
| (Lee et al, 2021) | Taiwan | Osteosarcopenic adiposity | EWGSOP | T: Experimental group underwent a 12-week progressive elastic band resistance exercise program under supervision. | RPE: 13 | 3 | 12 | 55 | BMD (g/cm²), Physical capacity assessments (various tests) | 15 | | 70.13 ± 4.41 | 26.95 ± 3.31 |
|  |  |  |  | C: attended a 40-min group lecture and received a booklet with educational content regarding OSA and home exercise instructions with pictorial demonstrations | / |  |  |  |  | 12 | | 71.82 ± 5.23 | 28.93 ± 3.55 |
| (Liao, Tsauo et al. 2018) | Taiwan | Sarcopenic obesity | EWGSOP | T: The EG performed elastic band resistance training, which included upper limb exercises (chest press, shoulder press, rowing, etc.), lower limb exercises (knee extension, knee flexion, hip flexion and extension), and trunk muscle groups. | Moderate intensity, Borg RPE ~13 (somewhat hard) corresponding to 60-70% 1RM equivalent | 3 | 12 | 55 | Muscle mass: Appendicular Lean Mass (ALM, kg); Total Skeletal Mass (TSM, kg); Muscle Indices: AMI, LMI, SMI (%), Fat Mass (%); Muscle quality: Upper and lower extremity strength normalized by muscle mass (kg/kg or N/kg); Physical capacity: Functional Forward Reach (cm), Single Leg Stance (s), Gait Speed (m/s), Timed Up & Go (s), Timed Chair Rise (repetitions); Quality of life: SF-36 Physical Function (PF) and Physical Component Summary (PCS) scores |  | 33 | 66.67 ± 4.54 | 27.27 ± 3.72 |
|  |  |  |  | C: did not undergo any exercise intervention and only received routine care | / |  |  |  |  |  | 23 | 68.32 ± 6.05 | 29.16 ± 3.62 |
| (Liao, Tsauo et al. 2017) | Taiwan | Sarcopenic obesity | AWGS | T: The EG underwent 12 weeks of elastic resistance training using Theraband, with the training program covering multiple muscle groups commonly used in daily functional activities. | Moderate (Borg RPE ~13) | 3 | 12 | ~50 | Fat-free mass (kg), Leg lean mass (kg), Total fat mass (kg), % Body fat, Handgrip strength (kg), Lower extremity strength (N), Gait speed (m/s), SLS (s), TUG (s) |  | 25 | 66.39 ± 4.49 | 27.32 ± 3.33 |
|  |  |  |  | C: received no formal exercise intervention and maintained their regular lifestyle. | / |  |  |  |  |  | 21 | 68.42 ± 5.86 | 28.19 ± 3.27 |
| (Flor-Rufino et al, 2023) | Spain | Sarcopenia | EWGSOP | T: The intervention group (HIRT) underwent a 6-month (approximately 24-week) high-intensity resistance training program, with a frequency of twice per week and each session lasting 65 minutes | > 70% 1RM | 2 | 24 | 65 | Muscle mass (kg), Skeletal muscle index (kg/m²), Handgrip strength (kg), Knee extension strength (kg), Leg press 1RM (kg), Gait speed (m/s), SPPB score (points), MRI biomarkers: Proton density fat fraction (unitless), T2* relaxation time (ms), Apparent diffusion coefficient (10⁻³ mm²/s), Diffusion coefficient (10⁻³ mm²/s), Muscle volume (L), Fat volume (L) |  | 20 | 79.9 ± 7.2 | 26.2 ± 4.2 |
|  |  |  |  | C: The control group received no specific exercise intervention, with only telephone follow-ups to assess health status. | / |  |  |  |  |  | 18 | 79.6 ± 7.7 | 27.7 ± 2.9 |
| (Ghasemikaram et al, 2021) | Germany | Osteosarcopenia | author defined cut offs | T: Supervised single-set high intensity resistance training (HIRT), 2 sessions /week, 16 months. | High effort, range of repetitions (5-7 or 8-10 reps), work to failure | 2 | 64 | / | Thigh muscle and adipose tissue volume (cm³), fat fraction (%) | 21 |  | 77.8 ± 3.6 | 25 ± 3 |
|  |  |  |  | C: No exercise intervention. | / |  |  |  |  | 22 |  | 79.2 ± 4.7 | 24.5 ± 1.9 |
| (Yamada et al, 2019) | Japan | Sarcopenic or dynapenic | AWGS | T-1: groups performed body-weight resistance exercise | / | 2 | 12 | 30 | Skeletal muscle quality, muscle mass, muscle strength | 8 | 20 | 84.9 ± 5.6 | 21.3 ± 3.2 |
|  |  |  |  | T-2: groups performed body-weight resistance exercise + received protein (10g whey) and vitamin D (800 IU) supplements | / |  |  |  |  | 10 | 18 | 84.7 ± 5.1 | 22.6 ± 3 |
|  |  |  |  | T-3: received protein (10g whey) and vitamin D (800 IU) supplements | / |  |  |  |  | 8 | 20 | 83.2 ± 5.7 | 22.6 ± 4.2 |
|  |  |  |  | C: had no intervention | / |  |  |  |  | 13 | 15 | 83.9 ± 5.7 | 21.2 ± 2.9 |
| (Chien et al, 2022) | Taiwan | T2DM combined with Sarcopenia | author defined cut offs | T: resistance training | Progressive, starting at 0.5 kg, increasing to 1 kg after 1 month. | 3 | 12 | 30 | HbA1c (%), Handgrip strength (kg), Five times sit-to-stand test (s), ASM (kg), ASMI (kg/m²), Calf circumference (cm), WHOQOL-BREF | 5 | 15 | 67.6 ± 7.7 | 24.3 ± 3.4 |
|  |  |  |  | C: usual care | / |  |  |  |  | 2 | 18 | 67.3 ± 6.1 | 25.5 ± 3.7 |
| (Lichtenberg et al, 2019) | Germany | Osteosarcopenic | EWGSOP | T: High-intensity resistance training on machines | 70–85% of 1RM | 2 | 28 | 50 | Sarcopenia Z-score, SMI (kg/m²), gait velocity (m/s), handgrip strength (kg) | 21 |  | 77.8 ± 3.6 | / |
|  |  |  |  | C: maintained normal lifestyle | / |  |  |  |  | 22 |  | 79.2 ± 4.7 | / |
| (Vasconcelos et al, 2016) | Brazil | Sarcopenic obesity |  | T: 10-week progressive resistance exercise program with high-speed component, targeting lower-limb muscles, including open and closed chain exercises | Moderate to somewhat severe (Borg Scale) | 2 | 10 | 60 | Knee extensor strength (J), power (w), fatigue (%), SPPB (points), gait velocity (m/s), SF-36 (score) |  | 14 | 72 ± 4.6 | / |
|  |  |  |  | C: Health status monitoring via telephone calls | / |  |  |  |  |  | 14 | 72 ± 3.6 | / |
| (Dong et al, 2019) | China | Sarcopenia | AWGS | T: Exercises included one-leg raise-and-down and upper limb bouncing ball movement. Each exercise performed 10 × 10 cycles repeatedly. | Ankle weight of 0 kg and increased by 0.5 kg weekly until reaching 5 kg. | 3 | 12 | 65 | Maximal grip strength (kg), Daily pace (m/s), Physical activity level, Kt/V, Serum albumin (g/L), CRP (pg/mL), IL-6 (pg/mL), IL-10 (pg/mL), TNF-α (pg/mL) | 9 | 12 | / | 18.96 ± 3.08 |
|  |  |  |  | C: Routine hemodialysis care. | / |  |  |  |  | 12 | 8 | / | 20.49 ± 3.41 |
| (Rezaei et al, 2024) | Iran | Sarcopenia | author defined cut offs | T: Six specific exercises were included, targeting different muscle groups:Upper Body: Push-ups and Rowing.Lower Body: Squat and Lateral Lunge.Core: Lateral Flexion and Upper Body Extension.Each exercise consisted of 3 sets of 12 repetitions, with 1 minute of rest between each set. | 60–65% 1RM | 3 | 8 | 60 | Serum levels of muscle growth factors (ng/L, ng/ml, pmol/L) and functional indices (seconds, kg) | 10 |  | 72.5 ± 4.17 | 23.45 ± 0.9 |
|  |  |  |  | C: Maintained their regular lifestyle and recorded their exercise during the experiment period. | / |  |  |  |  | 9 |  | 76.5 ± 3.53 | 23.2 ± 2.8 |
| (Chiu et al, 2018) | Taiwan | Sarcopenic obese | author defined cut offs | T: Chair muscle strength training using 2–5 lbs sandbags on wrist or ankle joints and a grip ball. Exercises included arm curl, hand up, arm lateral raise, knee extension, calf raises, stepping, etc. Each exercise performed for three sets of 4–10 repetitions with about 30 s rest between each set. Total duration of each training session was approximately 60 minutes. | Progressive, RPE scale 12–13 | 2 | 12 | 60 | Body composition (%), Muscular strength (kg), Functional Independence Measure (FIM) scores | 14 | 22 | 79.64 ± 7.36 | 25.15 ± 3.75 |
|  |  |  |  | C: usual care. | / |  |  |  |  | 21 | 13 | 80.15 ± 8.26 | 24.85 ± 3.01 |
| (He et al, 2024) | China | Sarcopenia | AWGS | T-1: Self-determined sequence exercise program (strength training, Yijinjing, hybrid). | / |  |  |  | SMA (cm²), SMD (HU), RSMI (kg/m²), MFI (kg), GS (kg) | 19 | 15 | 67.76 ± 5.47 | 21.96 ± 2.28 |
|  |  |  |  | T-2: Strength training. | / | 3 | 24 | / |  | 13 | 17 | 66.87 ± 3.84 | 22.8 ± 3.18 |
|  |  |  |  | C: No intervention | / |  |  |  |  | 16 | 14 | 65.42 ± 3.97 | 21.93 ± 2.86 |
| (Vikberg et al, 2019) | Sweden | Pre-sarcopenia | EWGSOP | T: 10-week instructor-led resistance training, 3 times/week, 60 min/session, targeting whole body muscles | High intensity (specific % or RM not provided) | 3 | 10 | 90 | SPPB (score), TUG (s), Chair sit-stand time (s), Lean body mass (kg), Fat mass (kg), Handgrip strength (kg) | 16 | 20 | 70.9 ± 0.28 | 22.72 ± 2.35 |
|  |  |  |  | C: presumed usual care or no intervention | / |  |  |  |  | 16 | 18 | 70 ± 0.29 | 23.33 ± 3.01 |
| (Bellomo et al, 2013) | Italy | Sarcopenic | EWGSOP | T-1: Global Sensorimotor Training):5-minute bicycle warm-up (60% HR_max), lower limb stretching exercises, 20-minute Imoove system training, 5-minute cool-down. | 60% HR_max_ | 2 | 12 | 30 | Maximum isometric strength (kg); Swing area (mm²); Ellipse area (mm²); Half step length (cm); Step width (cm); Single-leg support time (s) | 10 | | 70.9 ± 5.2 | 28.2 ± 5.9 |
|  |  |  |  | T-2: 10-minute bicycle warm-up (60% HR_max), lower limb stretching exercises, leg press and leg extension exercises (60-85% FMT) | 60-85% FMT | 2 | 12 | 30-40 |  | 10 | | 70.9 ± 5.2 | 28.2 ± 5.9 |
|  |  |  |  | T-3: Vibratory Mechanical-Acoustic Focal Therapy: 15-minute VISS device vibration stimulation (300 Hz, 70 mbar) | 300 Hz, 70 mbar | first 8 weeks 1 time/week, last 4 weeks 3 times/week | 12 | 15 |  | 10 | | 70.9 ± 5.2 | 28.2 ± 5.9 |
|  |  |  |  | C: Maintain daily activities, provide informational bulletins | / |  |  |  |  | 10 | | 70.9 ± 5.2 | 28.2 ± 5.9 |
| (Seo et al, 2021) | Korea | Sarcopenia | FNIH | T: 16 weeks of resistance training, 3 times /week, 60 min /session, using body weight-based and elastic band training | OMNI-RES AM scale from 4 to 8 | 3 | 16 | 60 | functional fitness, muscle quality, muscle growth factors (e.g., grip strength (kg), gait speed (m/s), isometric muscle strength (N·m), thigh muscle volume (cm²), intramuscular fat (cm²)) |  | 12 | 70.3 ± 5.38 | 22.9 ± 2.02 |
|  |  |  |  | C: no exercise intervention | / |  |  |  |  |  | 10 | 72.9 ± 4.75 | 22.4 ± 1.52 |
| (Wei et al, 2022) | China | Sarcopenia | AWGS | T-1: 30 min Yi Jin Jing + 30 min resistance training, 3 times /week, 24 weeks. | / |  |  |  | L3 SMA (cm²), L3 SMD (HU), L3 SMFA (cm²), L3 SMFD (HU), RSMI (kg/m²), MFI (%), HGS (kg) | 14 | 16 | 66.7 ± 4.1 | 22.76 ±2.19 |
|  |  |  |  | T-2: 60 min resistance training, 3 times/ week, 24 weeks. | 40-85% 1RM | 3 | 24 | 90 |  | 13 | 17 | 66.87 ± 3.84 | 22.8 ± 3.18 |
|  |  |  |  | C: Education on sarcopenia and prevention methods. | / |  |  |  |  | 16 | 14 | 65.42 ± 3.97 | 21.93 ± 2.86 |
| (Cebrià I Iranzo et al, 2018) | Spain | Sarcopenia | EWGSOP | T-1: 10 isotonic resistance exercises, 12 repetitions each, using dumbbells and ankle/wrist weights, slow speed, full range of motion, concentric and eccentric phases, 2-minute rest between exercises | 40-60% of maximal isometric muscle strength | 3 | 12 | 20-30 | Skeletal muscle mass (ASM/height², kg/m²; ASM/weight, %; ASM/BMI, m²); Isometric knee extension (kg); Arm flexion (kg); Handgrip strength (kg); Maximal inspiratory pressure (MIP, cmH₂O); Maximal expiratory pressure (MEP, cmH₂O); Gait speed (m/s) | 11 | | 82.6 ± 9.1 | 31.4 ± 7.5 |
|  |  |  |  | T-2: Interval-based inspiratory muscle training using Threshold Inspiratory Muscle Trainer, seven 2-minute cycles with 1-minute rest between cycles | 40-60% of maximal inspiratory pressure (MIP) |  |  |  |  | 9 | | 87.1 ± 3.8 | 32 ± 8.3 |
|  |  |  |  | C: Standard treatment, maintaining usual care and daily activities (e.g., lying down, sitting, short walks). | / |  |  |  |  | 17 | | 81.2 ± 5.4 | 31.2 ± 5.9 |
| (Hamaguchi et al, 2017) | Japan | Sarcopenia | AWGS | T: Low-repetition, light-load power training with weighted vest, 6 weeks, 2 sessions /week. | Light load (progressive with weighted vest) | 2 | 6 | 60 | BMD (g/cm²), Muscle strength (Nm/kg, kg), SMI (kg/m²) |  | 7 | 60.4 ± 2.7 | 19.2 ± 1.2 |
|  |  |  |  | C: No intervention | / |  |  |  |  |  | 8 | 60.6 ± 2.3 | 19.7 ± 1.8 |
| (Huang et al, 2017) | Taiwan | Sarcopenic obesity | author defined cut offs | T: A 12-week progressive elastic band resistance training program, conducted 3 times per week, with each session lasting 55 minutes, including 10 minutes of warm-up, 40 minutes of elastic band resistance training, and 5 minutes of cool-down. | Increase resistance when RPE 13 | 3 | 12 | 55 | Fat percentage (%), bone mineral density (BMD, T-score, Z-score) |  | 18 | 68.89 ± 4.91 | 27.31 ± 3.74 |
|  |  |  |  | C: Attend a 40-minute session on sarcopenic obesity and the concept of home-based exercise. | / |  |  |  |  |  | 17 | 69.53 ± 5.09 | 28.96 ± 3.49 |
| (Guo et al, 2024) | Japan | Sarcopenic | FNIH | T-1: training consists of 24 weeks, 3 sessions per week, each lasting 90 minutes. Tai Chi practice for 30 minutes. | / | 3 | 24 | 90 | Grip strength (kg), RSMI (kg/m²), L3 SMA (cm²), Muscle Fat Infiltration (%) | 14 | 19 | 66.94 ± 4.42 | 23.23 ± 2.06 |
|  |  |  |  | T-2: strength training combining elastic bands and machine or free weight exercises. | Gradually progressing from 40% to 85% 1RM |  |  |  |  | 13 | 17 | 66.87 ± 3.84 | 22.8 ± 3.18 |
|  |  |  |  | C: only receives health information guidance. | / |  |  |  |  | 16 | 14 | 65.42 ± 3.97 | 21.93 ± 2.86 |
| (Mori & Tokuda, 2022) | Japan | Sarcopenia | AWGS | T-1: Resistance training twice weekly for 24 weeks using elastic bands and bodyweight exercises combined with Leucine-enriched whey protein supplement. | Moderate (elastic bands and body resistance | 2 | 24 | 30-40 | ASMI (kg/m²), HGS (kg), KES (kg), UWS (m/sec) | 23 | | 77.7 ± 3.3 | 20.3 ± 2.4 |
|  |  |  |  | T-2: Resistance training twice weekly for 24 weeks using elastic bands and bodyweight exercises. | Moderate (elastic bands and body resistance |  |  |  |  | 23 | | 77.6 ± 5.2 | 20.3 ± 2.9 |
|  |  |  |  | C: No resistance training or supplementation during detraining. | / |  |  |  |  | 24 | | 77.8 ± 4.5 | 20.1 ± 3.1 |
| (Gadelha et al, 2021) | Brazil | Sarcopenia | EWGSOP | T: 24-week resistance training immediately before dialysis. | Initial RPE 5–6 (moderate); Weeks 13–24 RPE 7–8 (vigorous) | 3 | 24 | 40 | ASM (kg) / height^2 (m²), Handgrip strength(kg), TUG(s), TNF-α(pg/mL), IL-6(pg/mL), IL-10(pg/mL), Serum ferritin (Ferritin: ng/mL),Serum hepcidin (ng/mL), Serum iron levels(µg/dL), 5-year all-cause mortality rate | 37 | | 65 ± 3.6 | 23.1 ± 2.2 |
|  |  |  |  | C: Usual care with multidisciplinary recommendations. | / |  |  |  |  | 28 | | 63.8 ± 4.1 | 25.1 ± 3 |

Note: C, Control group; T, Experimental group; European Working Group on Sarcopenia in Older People [EWGSOP]; Asian Working Group for Sarcopenia [AWGS]; Foundation for the National Institutes of Health [FNIH].

**S3 Meta-analysis of forest plots on body composition.**

| 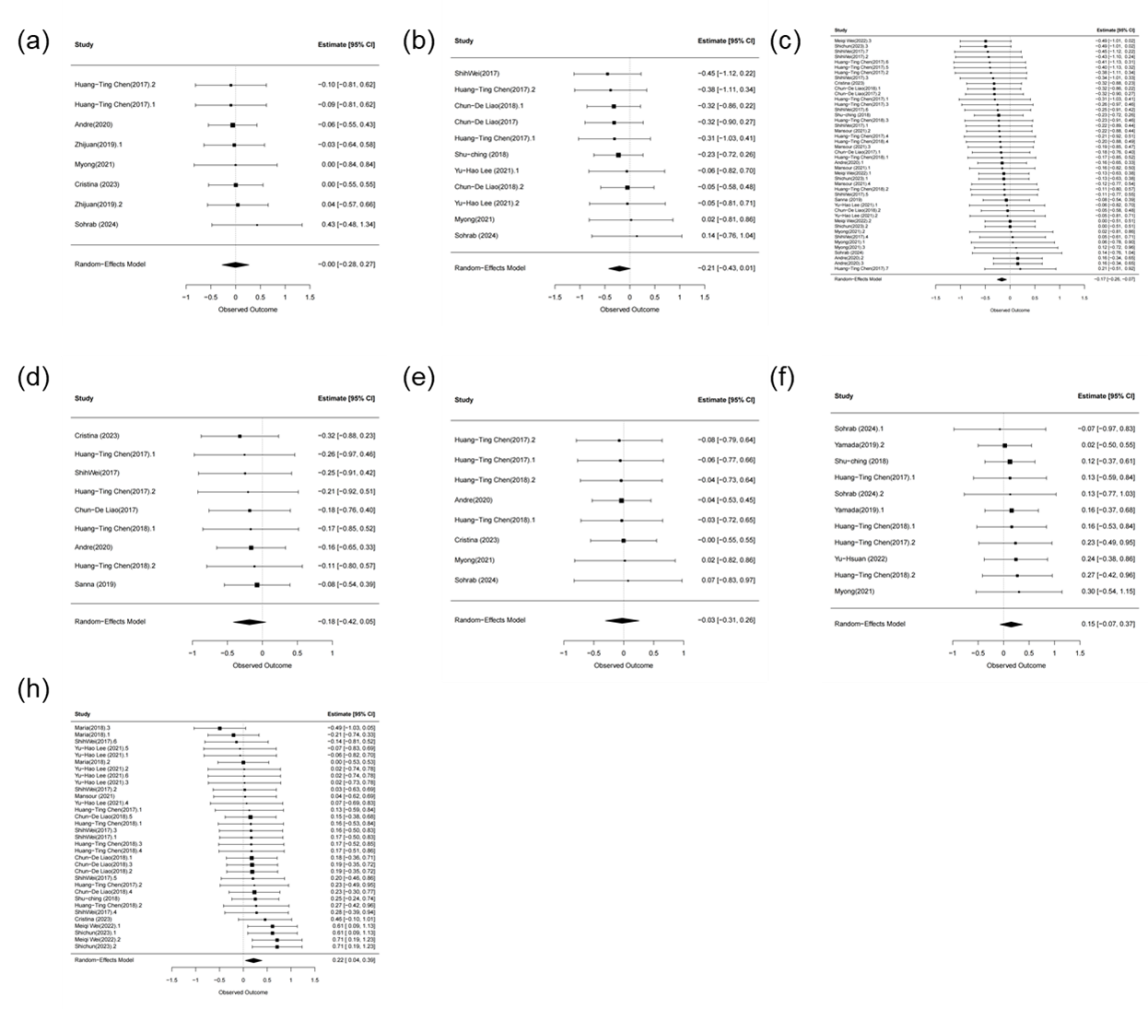 |
| --- |
| Note: a - h outcomes: BMI, Percent Body Fat, Fat Mass, Body Fat Mass, Body Weight, Skeletal Muscle Mass, Muscle Mass. |

**S4 Meta-analysis of forest plots on muscle quantity index.**

| 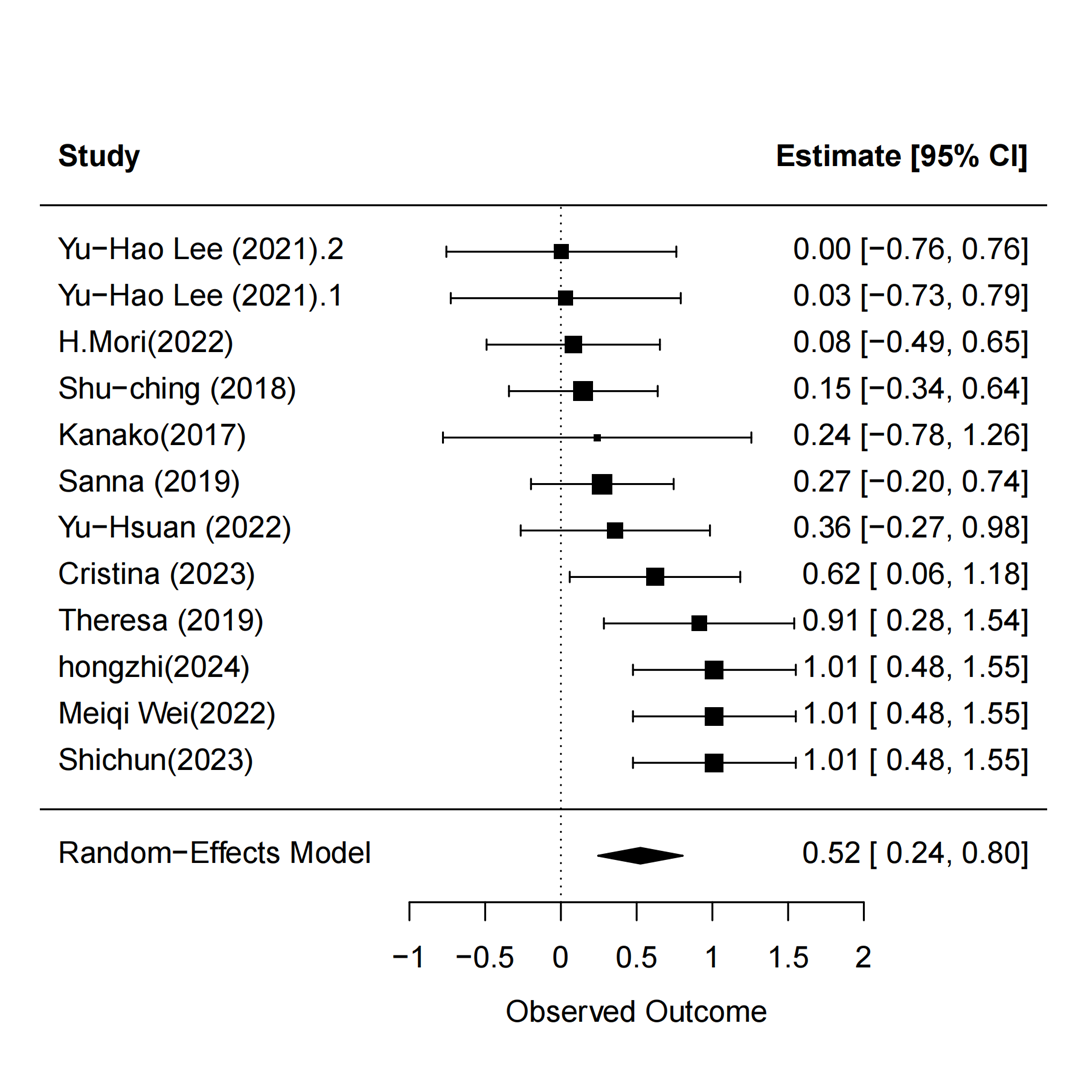 |
| --- |
| Note: outcomes: Skeletal Muscle Index. |

**S5 Meta-analysis of forest plots on muscle function metrics.**

| 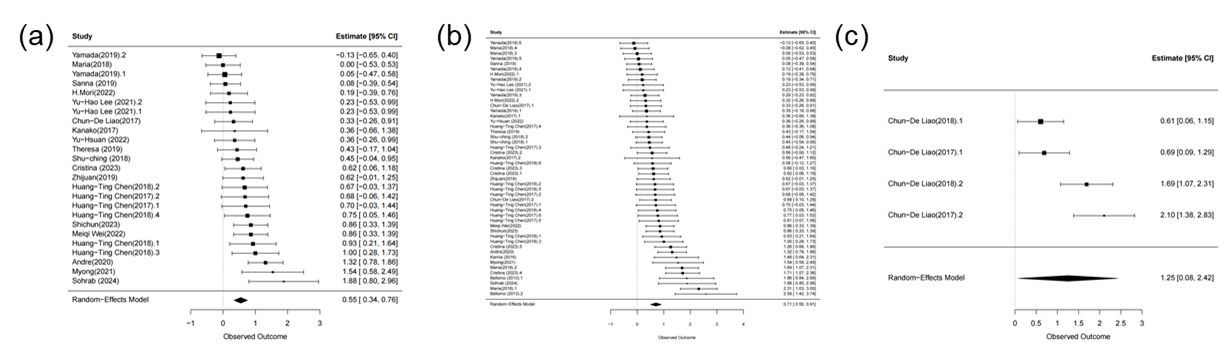 |
| --- |
| Note: a - c outcomes: Grip strength, Muscle strength, Muscle quality. |

**S6 Meta-analysis of forest plots on physical performance metric.**

| 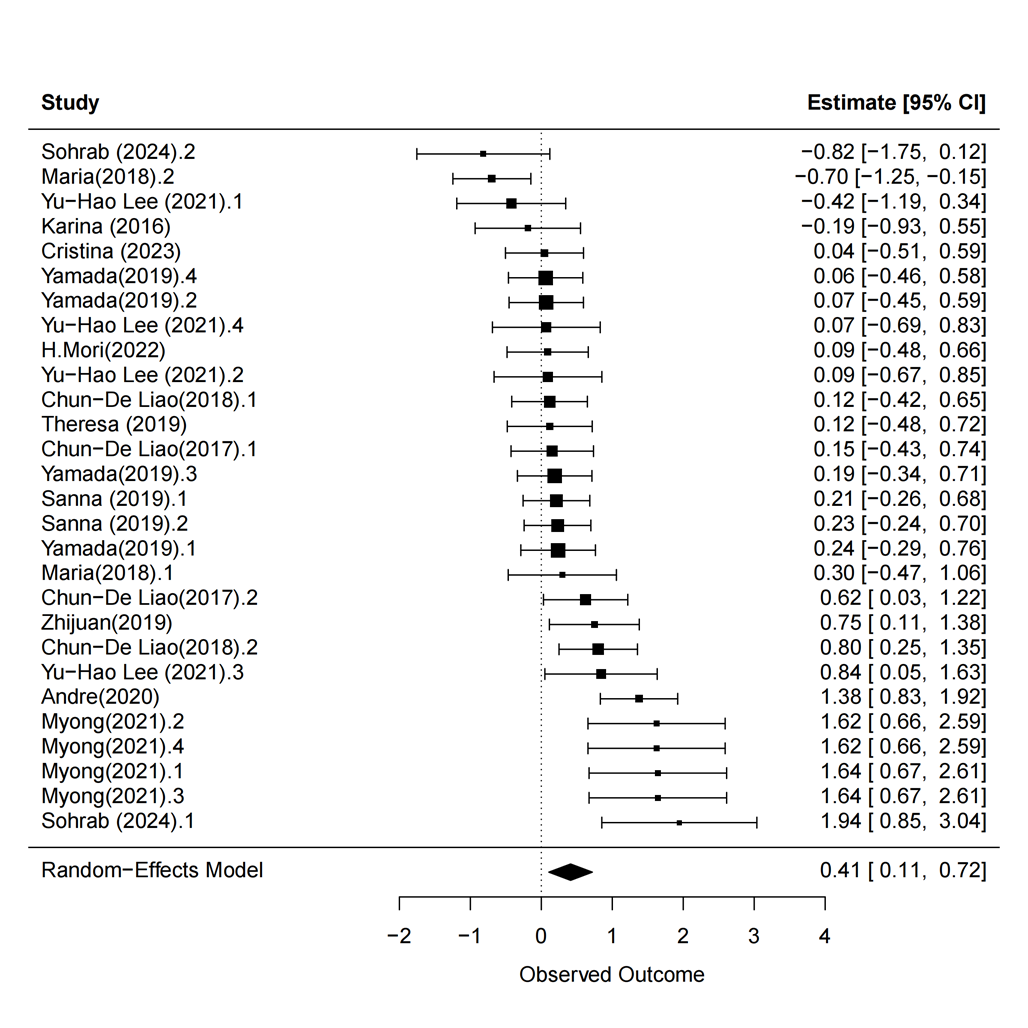 |
| --- |
| Note: outcomes: Walking ability. |

**S7. Publish the funnel plots of bias test on body composition.**

| 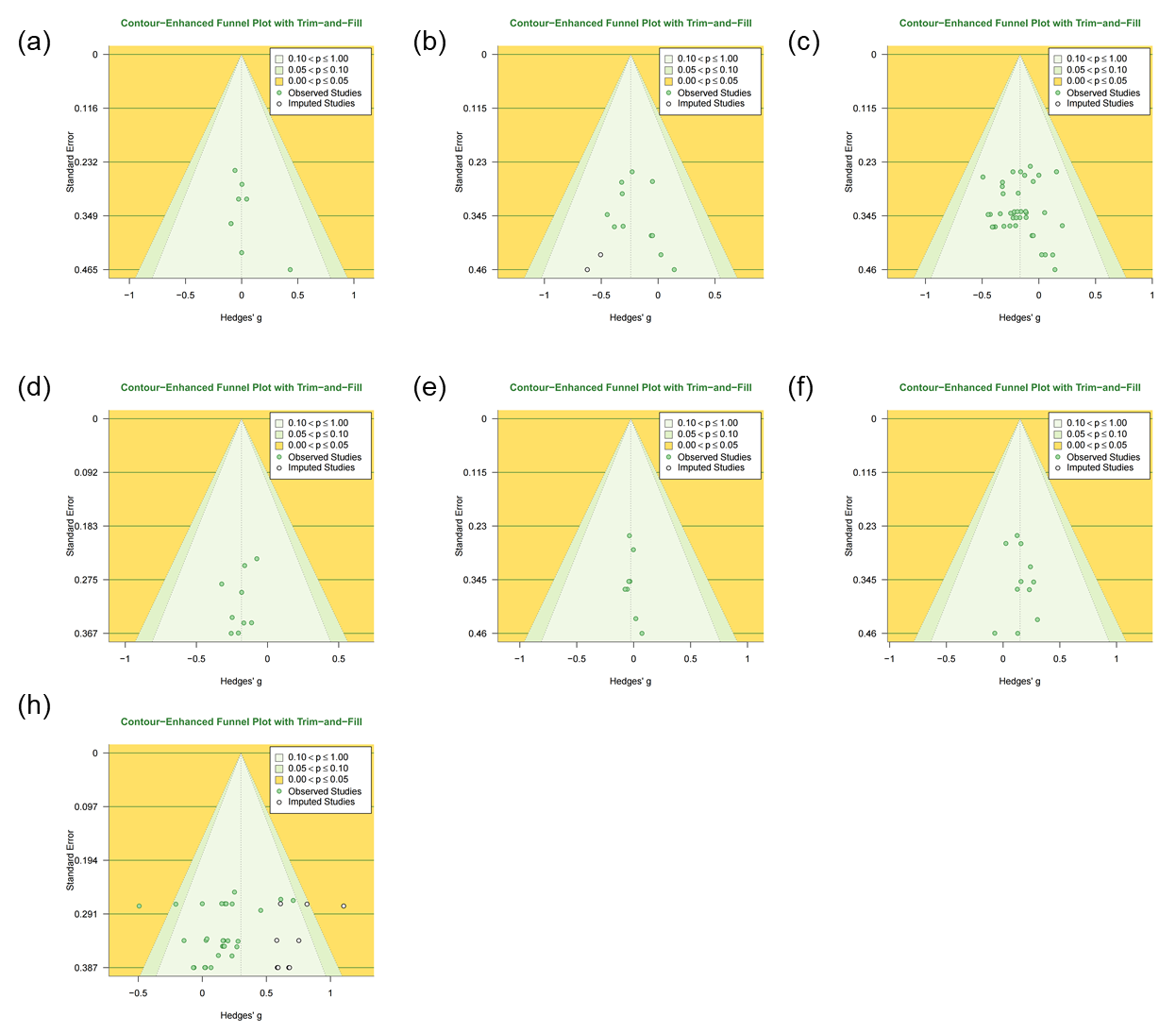 |
| --- |
| Note: a - h outcomes: BMI, Percent Body Fat, Fat Mass, Body Fat Mass, Body Weight, Skeletal Muscle Mass, Muscle Mass. |

**S8. Publish the funnel plots of bias test on muscle quantity index.**

| 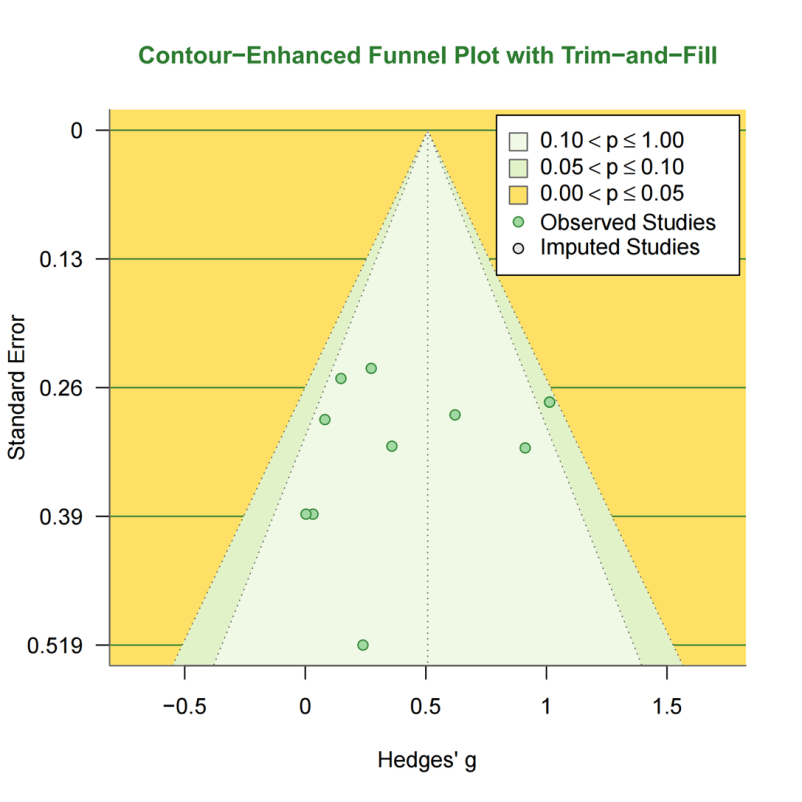 |
| --- |
| Note: outcomes: Skeletal Muscle Index. |

**S9. Publish the funnel plots of bias test on muscle function metrics.**

| 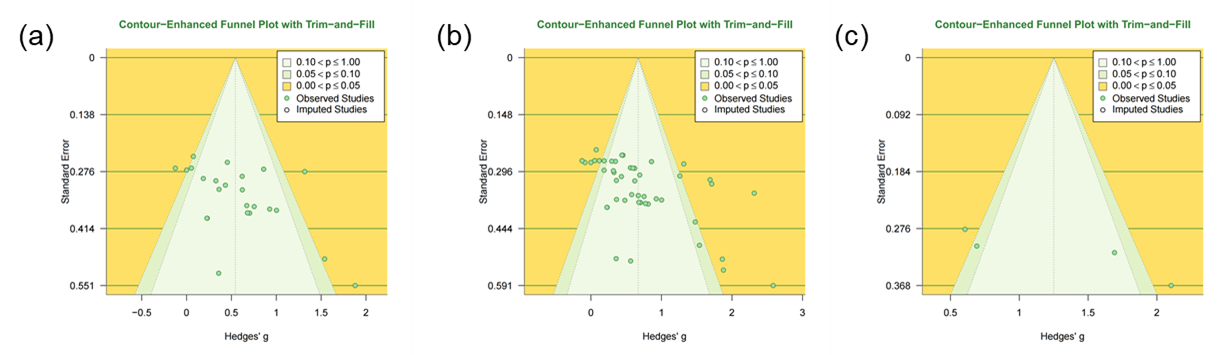 |
| --- |
| Note: a - c outcomes: Grip strength, Muscle strength, Muscle quality. |

**S10. Publish the funnel plots of bias test on physical performance metric.**

| 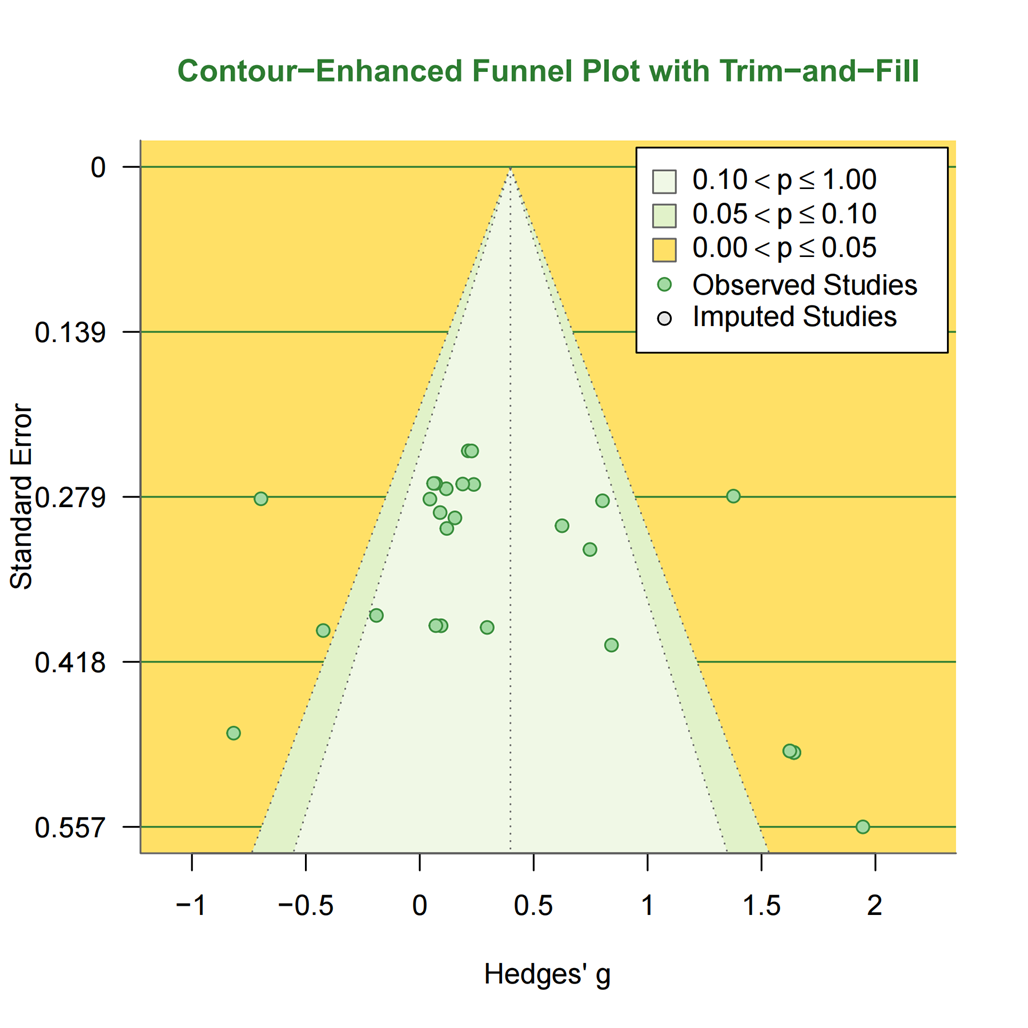 |
| --- |
| Note: outcomes: Walking ability. |

**S11. Results of sensitivity analysis on body composition**

| 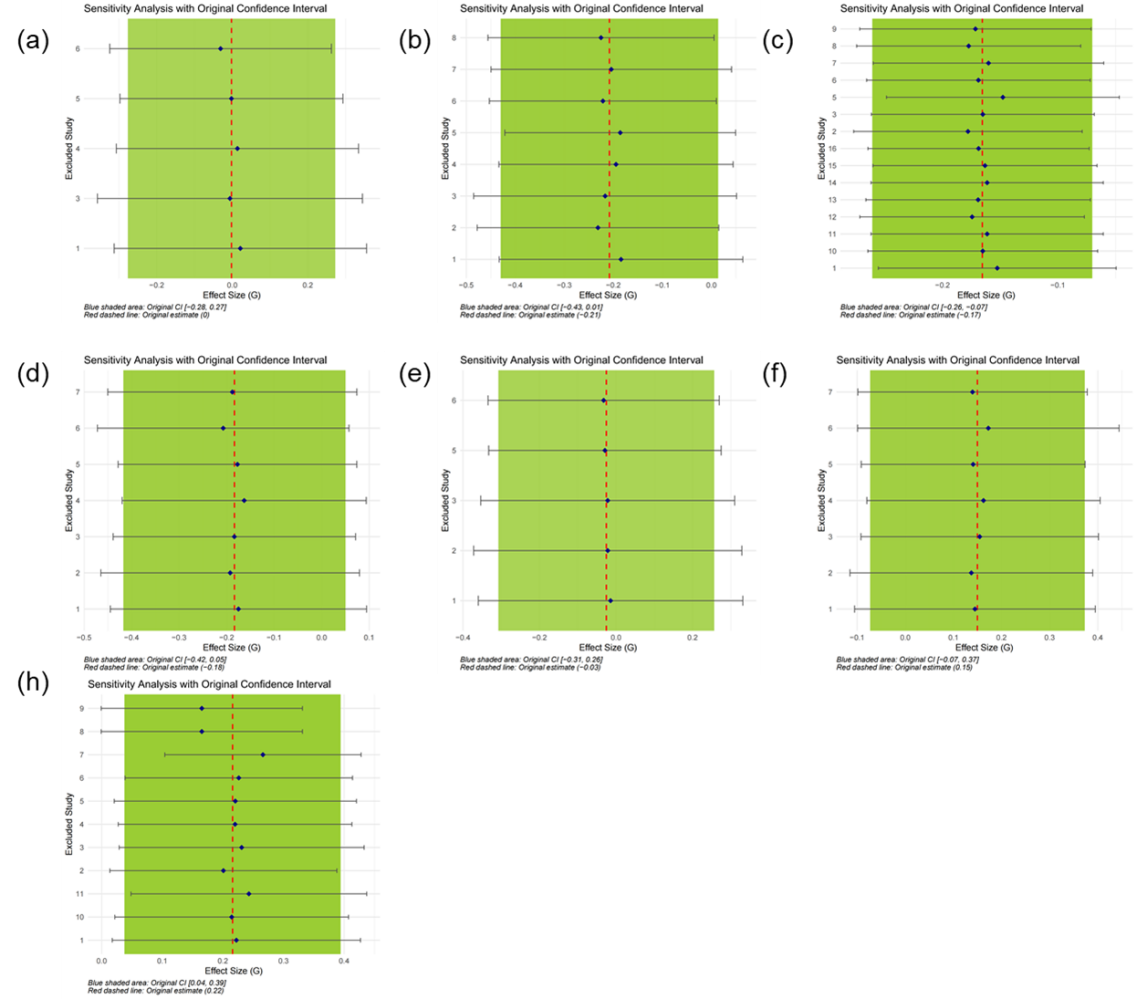 |
| --- |
| Note: a - h outcomes: BMI, Percent Body Fat, Fat Mass, Body Fat Mass, Body Weight, Skeletal Muscle Mass, Muscle Mass. |

**S12. Results of sensitivity analysis on muscle quantity index**

| 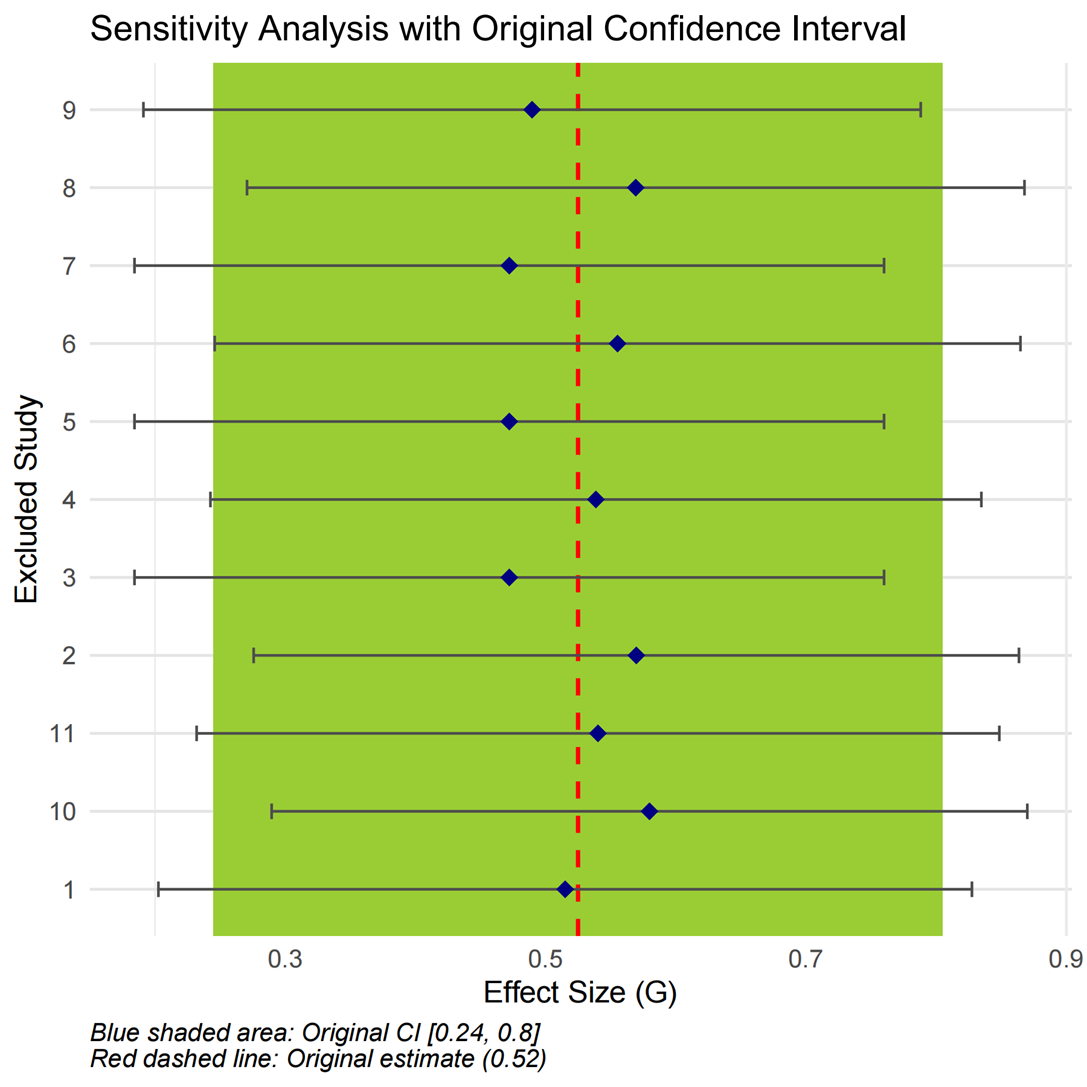 |
| --- |
| Note: outcomes: Skeletal Muscle Index. |

**S13. Results of sensitivity analysis on muscle function metrics**

| 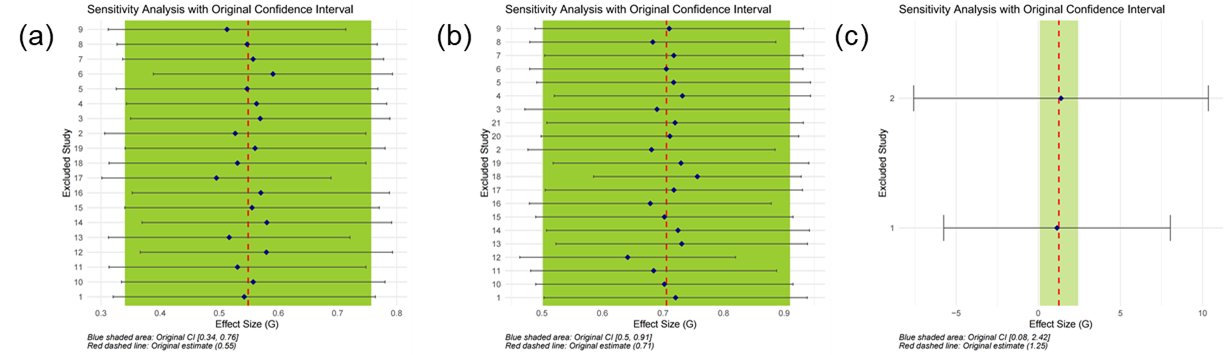 |
| --- |
| Note: a - c outcomes: Grip strength, Muscle strength, Muscle quality. |

**S14. Results of sensitivity physical performance metric**

| 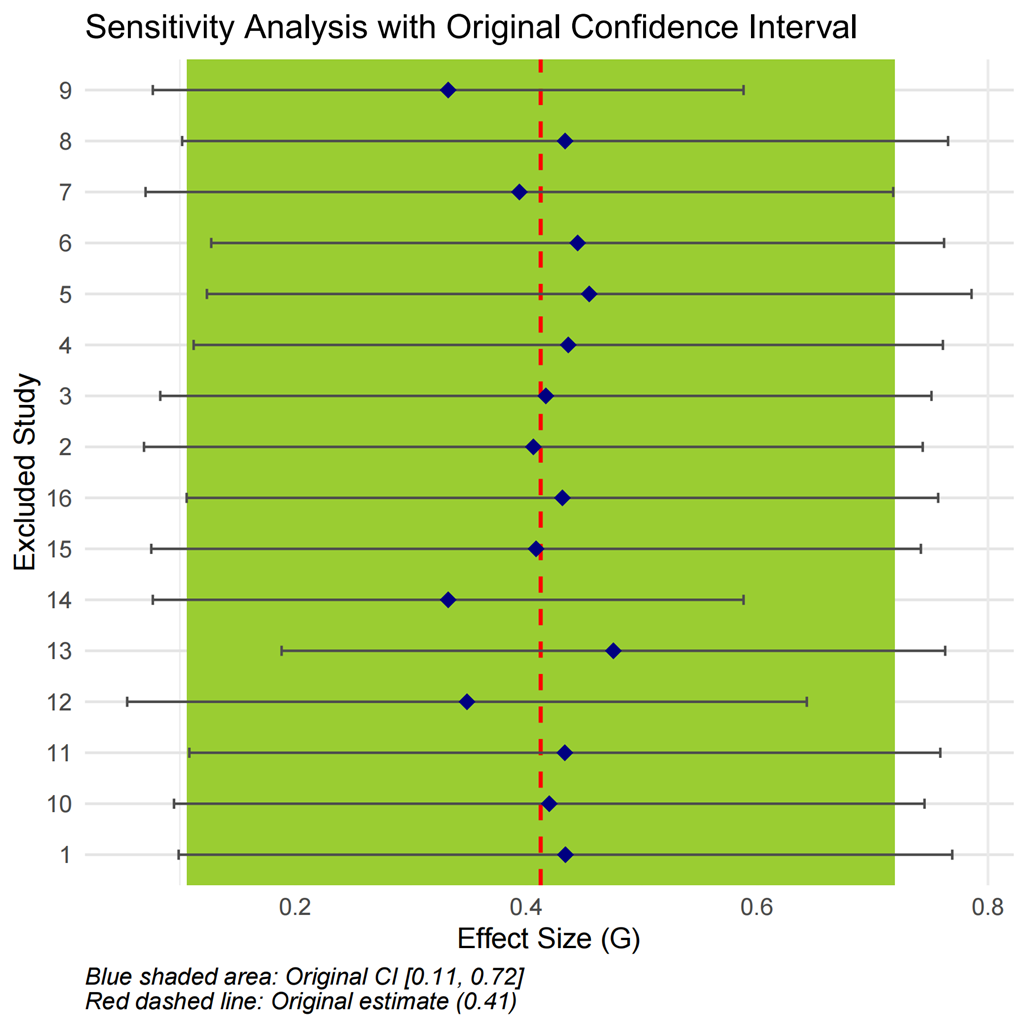 |
| --- |
| Note: outcomes: Walking ability. |

**S15. Results of regression analysis on the age of the population in body composition indicators.**

| 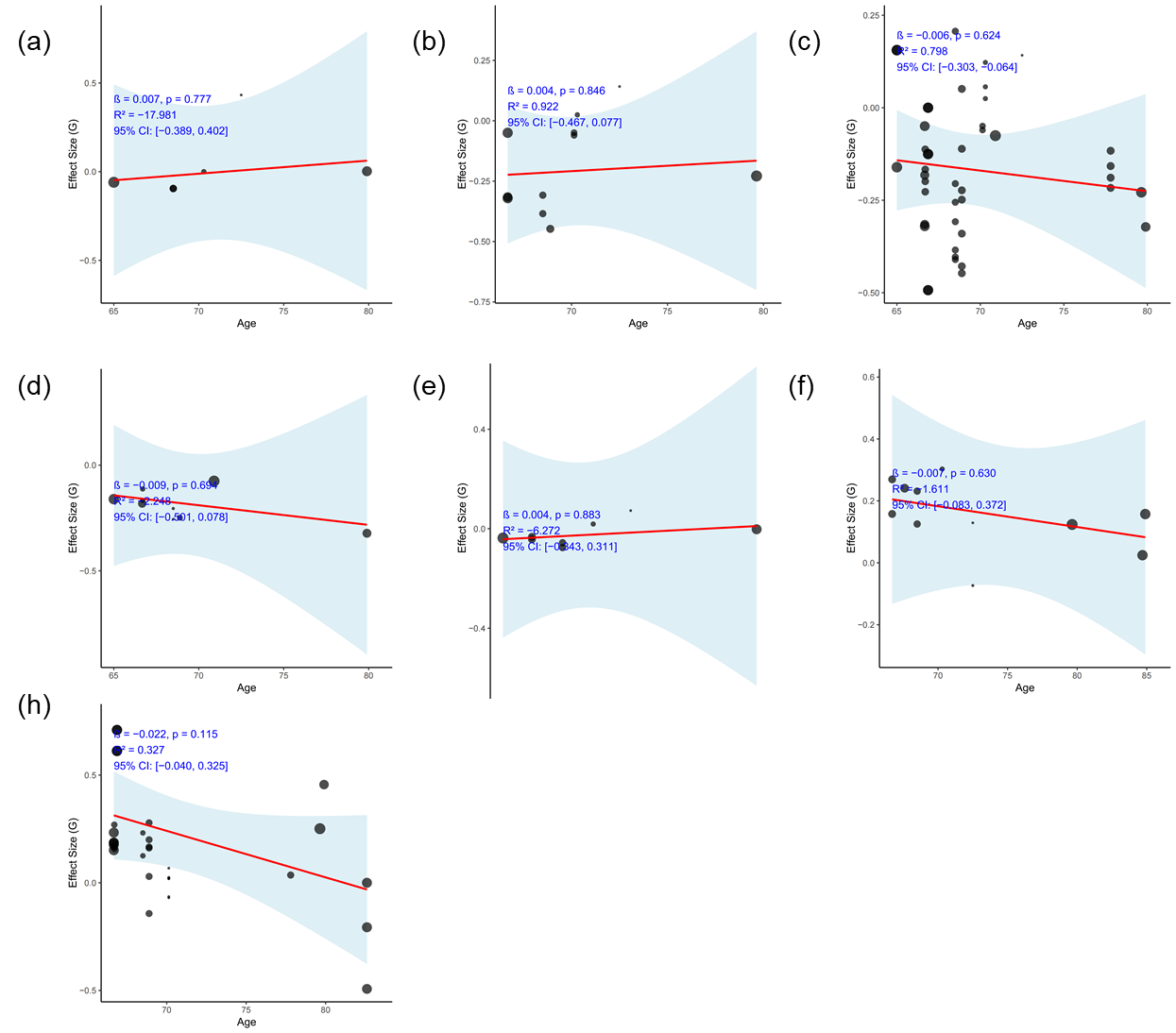 |
| --- |
| Note: a - h outcomes: BMI, Percent Body Fat, Fat Mass, Body Fat Mass, Body Weight, Skeletal Muscle Mass, Muscle Mass. |

**S16. Results of regression analysis on the age of the population in muscle quantity index**

| 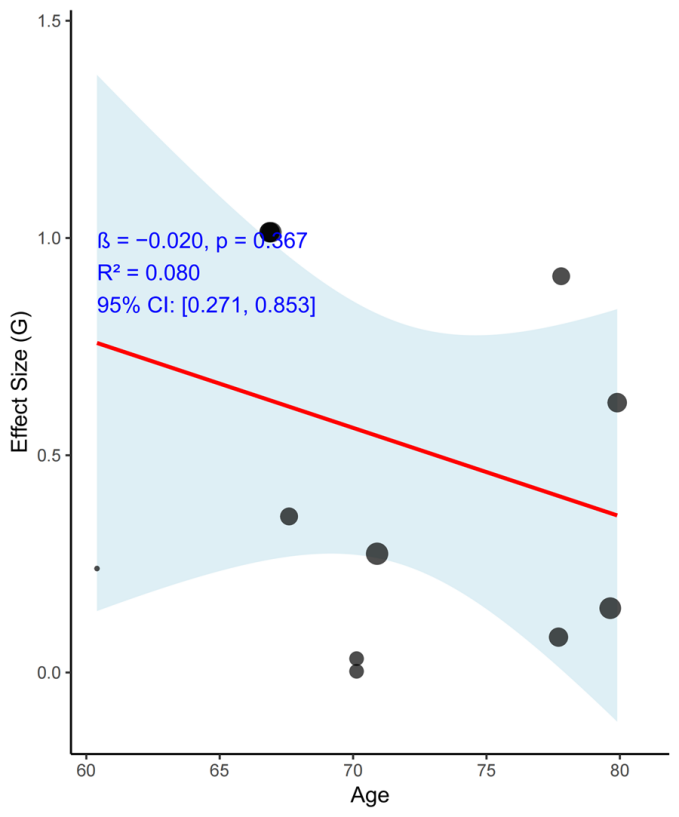 |
| --- |
| Note: outcomes: Skeletal Muscle Index |

**S17. Results of regression analysis on the age of the population in muscle function metrics**

| 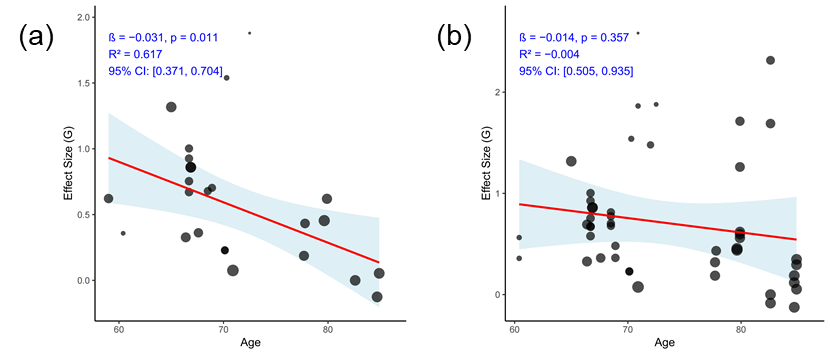 |
| --- |
| Note: a - c outcomes: Grip strength, Muscle strength. |

**S18. Results of regression analysis on the age of the population in physical performance metric**

| 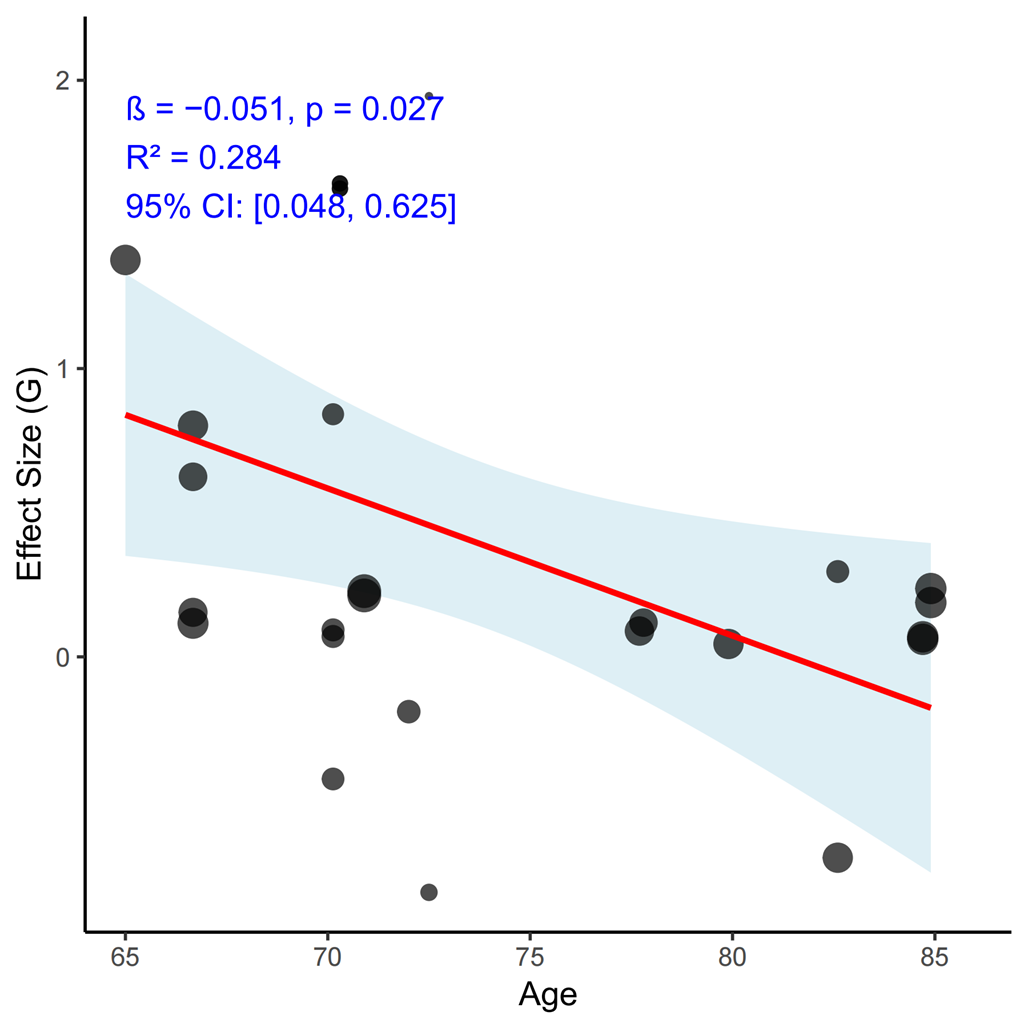 |
| --- |
| Note: outcomes: Walking ability. |

**S19. Results of regression analysis on the BMI of the population in body composition indicators.**

| 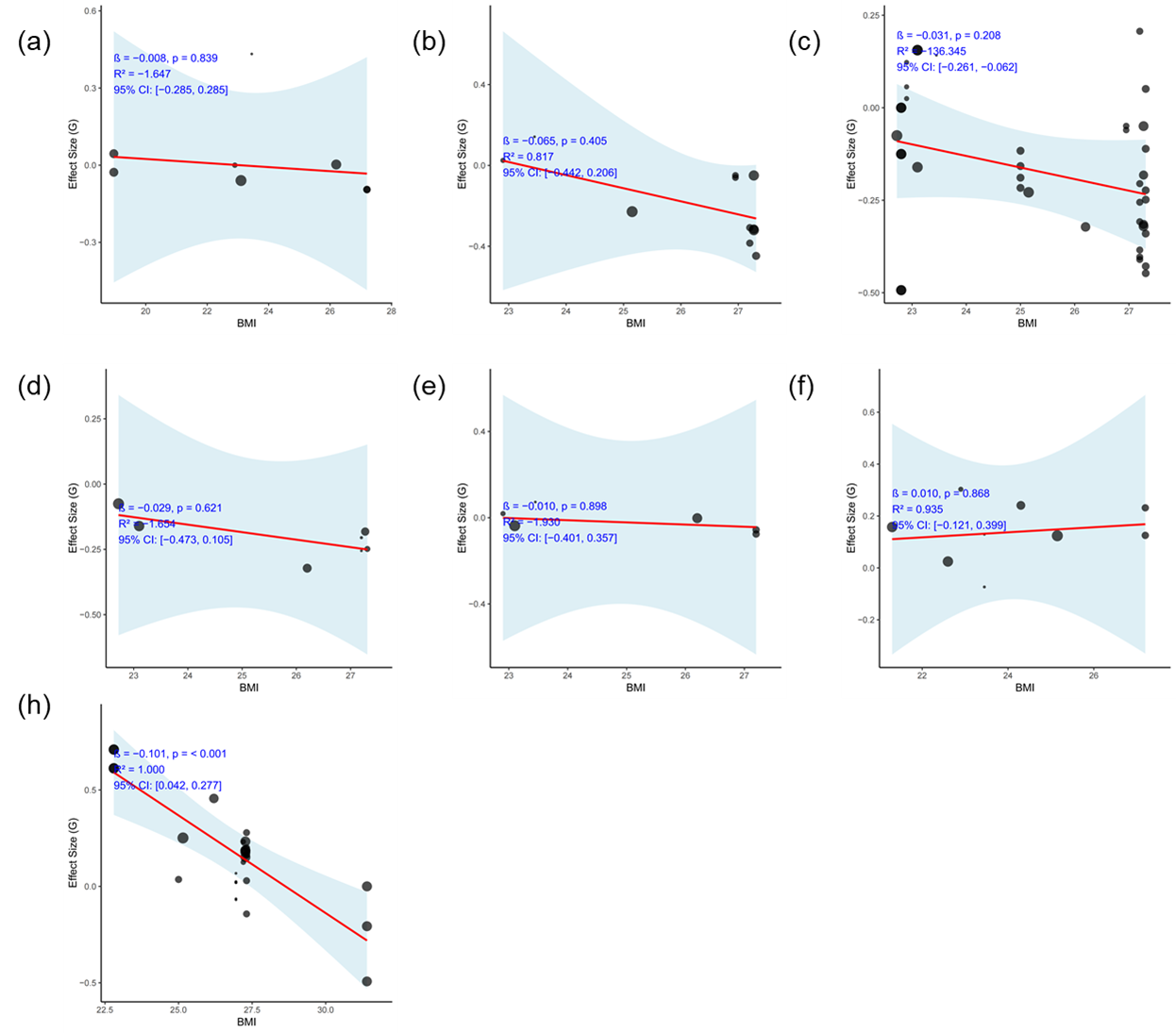 |
| --- |
| Note: a - h outcomes: BMI, Percent Body Fat, Fat Mass, Body Fat Mass, Body Weight, Skeletal Muscle Mass, Muscle Mass. |

**S20. Results of regression analysis on the BMI of the population in muscle quantity index**

| 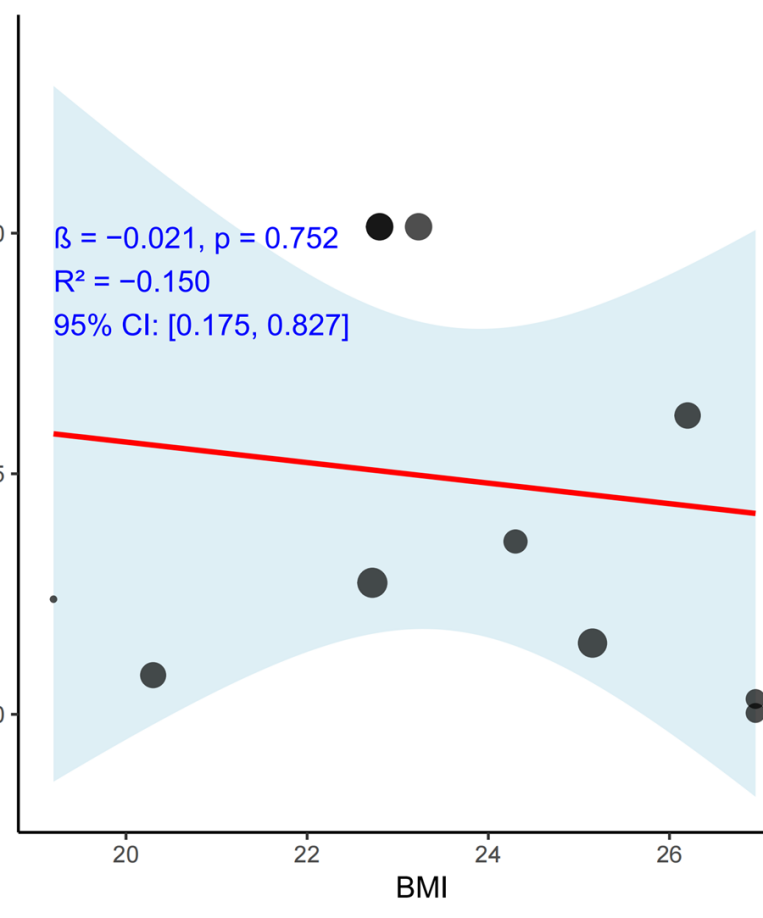 |
| --- |
| Note: outcomes: Skeletal Muscle Index |

**S21. Results of regression analysis on the BMI of the population in muscle function metrics.**

| 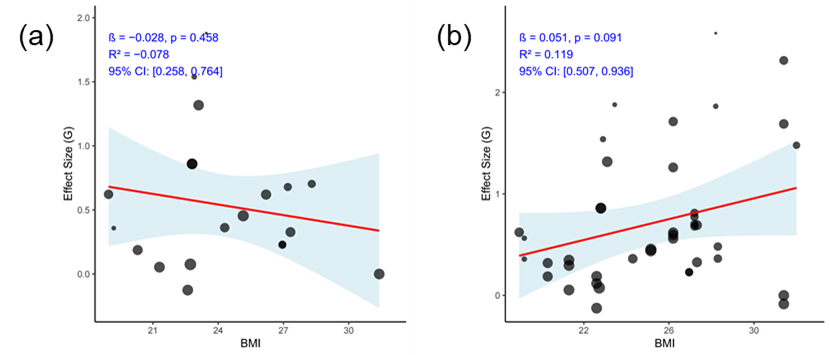 |
| --- |
| Note: a - c outcomes: Grip strength, Muscle strength. |

**S22. Results of regression analysis on the BMI of the population in physical performance metric.**

| 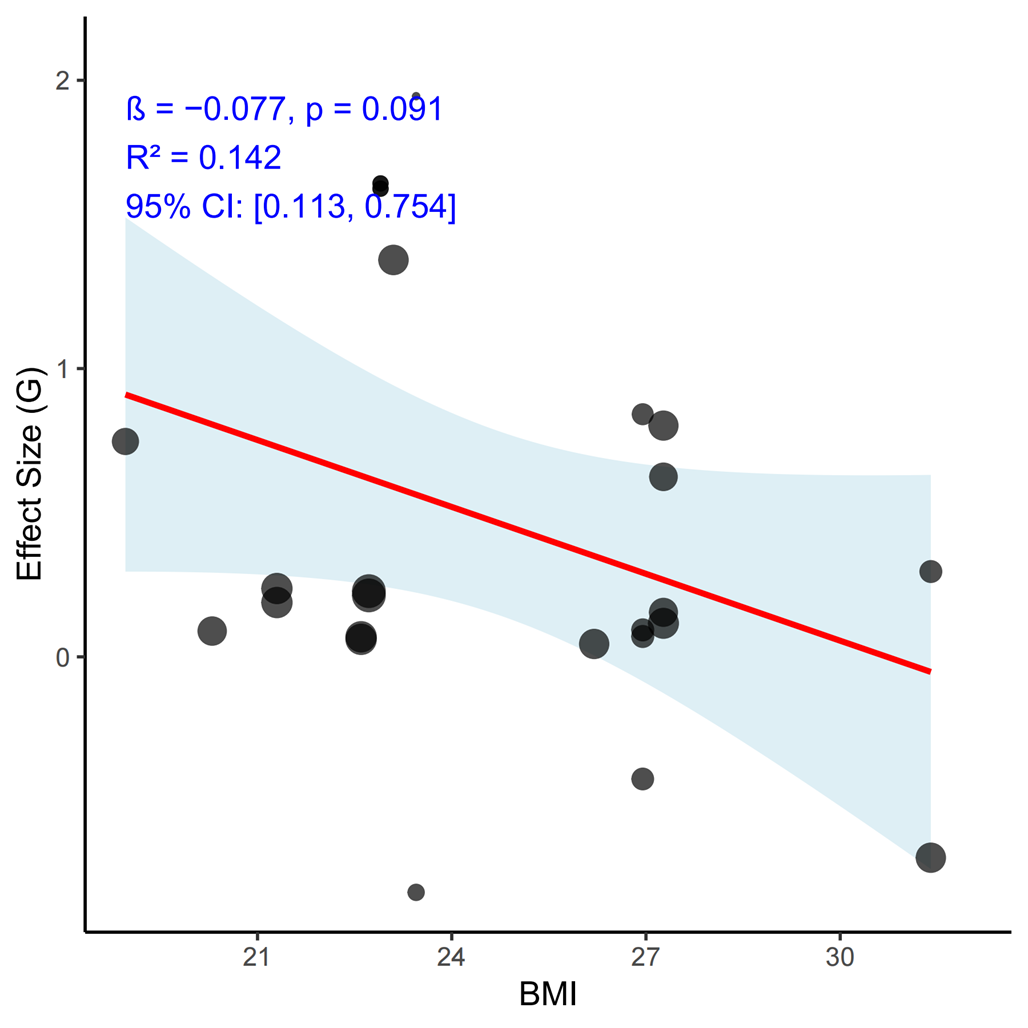 |
| --- |
| Note: outcomes: Walking ability. |

**S23 TESTEX scale result.**

| Study | Study Quality | | | | | Study Reporting | | | | | | | Overall |
| --- | --- | --- | --- | --- | --- | --- | --- | --- | --- | --- | --- | --- | --- |
|  | Eligibility criteria specified | Randomization specified | Allocation concealment | Groups similar at baseline | Blinding of assessor | Outcome measures assessed in 85% of patients | Intention-to-treat analysis | Between-group statistical comparisons reported | Point measures and measures of variability for all reported outcome measures | Activity monitoring in control groups | Relative exercise intensity remained constant | Exercise volume and energy expenditure |  |
| (H. Chen et al, 2017) | 1 | 1 | 1 | 1 | 1 | 0 | 0 | 1 | 1 | 0 | 0 | 1 | 8 |
| (H.-T. Chen et al, 2018) | 1 | 1 | 0 | 1 | 1 | 1 | 1 | 1 | 1 | 0 | 0 | 1 | 9 |
| (Lee et al, 2021) | 1 | 1 | 0 | 1 | 0 | 0 | 0 | 0 | 1 | 0 | 0 | 1 | 5 |
| (Liao et al, 2018) | 1 | 1 | 1 | 1 | 1 | 1 | 1 | 1 | 1 | 0 | 0 | 1 | 10 |
| (Liao et al, 2017) | 1 | 1 | 1 | 1 | 0 | 1 | 1 | 1 | 1 | 0 | 0 | 1 | 9 |
| (Flor-Rufino et al, 2023) | 1 | 1 | 0 | 1 | 1 | 0 | 0 | 1 | 1 | 0 | 0 | 1 | 7 |
| (Ghasemikaram et al, 2021) | 1 | 1 | 0 | 1 | 0 | 0 | 1 | 0 | 1 | 0 | 0 | 1 | 6 |
| (Yamada et al, 2019) | 1 | 1 | 1 | 1 | 1 | 0 | 0 | 0 | 1 | 0 | 0 | 1 | 7 |
| (Chien et al, 2022) | 1 | 1 | 0 | 1 | 0 | 1 | 1 | 0 | 1 | 0 | 0 | 1 | 7 |
| (Lichtenberg et al, 2019) | 1 | 1 | 0 | 1 | 1 | 0 | 1 | 1 | 1 | 1 | 0 | 1 | 9 |
| (Vasconcelos et al, 2016) | 1 | 1 | 1 | 1 | 0 | 0 | 0 | 1 | 1 | 0 | 0 | 1 | 7 |
| (Dong et al, 2019) | 1 | 1 | 0 | 1 | 0 | 1 | 0 | 1 | 1 | 0 | 0 | 1 | 7 |
| (Rezaei et al, 2024) | 1 | 1 | 0 | 1 | 0 | 0 | 0 | 1 | 1 | 1 | 0 | 1 | 7 |
| (Chiu et al, 2018) | 1 | 1 | 0 | 1 | 0 | 0 | 0 | 0 | 1 | 0 | 0 | 1 | 5 |
| (He et al, 2024) | 1 | 1 | 1 | 1 | 0 | 0 | 0 | 0 | 1 | 0 | 0 | 1 | 6 |
| (Vikberg et al, 2019) | 1 | 1 | 0 | 1 | 0 | 1 | 0 | 0 | 1 | 0 | 0 | 1 | 7 |
| (Bellomo et al, 2013) | 1 | 1 | 0 | 1 | 0 | 1 | 0 | 0 | 1 | 1 | 0 | 1 | 7 |
| (Seo et al, 2021) | 1 | 1 | 0 | 1 | 0 | 0 | 0 | 1 | 1 | 0 | 0 | 1 | 6 |
| (Wei et al, 2022) | 1 | 1 | 1 | 1 | 1 | 1 | 0 | 0 | 1 | 0 | 0 | 1 | 8 |
| (Cebrià I Iranzo et al, 2018) | 1 | 1 | 0 | 1 | 0 | 0 | 0 | 0 | 0 | 1 | 0 | 1 | 5 |
| (Hamaguchi et al, 2017) | 1 | 1 | 0 | 1 | 0 | 1 | 0 | 1 | 1 | 0 | 0 | 1 | 7 |
| (Huang et al, 2017) | 1 | 1 | 0 | 1 | 0 | 1 | 0 | 0 | 1 | 0 | 0 | 1 | 6 |
| (Guo et al, 2024) | 1 | 1 | 1 | 1 | 1 | 1 | 0 | 0 | 1 | 0 | 0 | 1 | 8 |
| (Mori & Tokuda, 2022) | 1 | 1 | 1 | 1 | 1 | 1 | 0 | 0 | 1 | 0 | 0 | 1 | 8 |
| (Gadelha et al, 2021) | 1 | 1 | 0 | 1 | 0 | 0 | 0 | 1 | 1 | 0 | 0 | 1 | 6 |

**S24 RoB2.0 result.**

| Study | Randomization process | Deviations from intended interventions | Measurement of the outcome | Missing outcome data | Selection of the reported result | Overall bias |
| --- | --- | --- | --- | --- | --- | --- |
| (H. Chen et al, 2017) | Low | Low | Low | Low | Low | Low |
| (H.-T. Chen et al, 2018) | Low | Low | Low | Low | Low | Low |
| (Lee et al, 2021) | Low | Unclear | Low | Low | Low | Unclear |
| (Liao et al, 2018) | Low | Low | Low | Low | Low | Low |
| (Liao et al, 2017) | Low | Low | Low | Low | Low | Low |
| (Flor-Rufino et al, 2023) | Low | Low | Low | Low | Low | Low |
| (Ghasemikaram et al, 2021) | Low | Low | Low | Low | Low | Low |
| (Yamada et al, 2019) | Low | Low | Low | Low | Low | Low |
| (Chien et al, 2022) | Low | Low | Low | Low | Low | Low |
| (Lichtenberg et al, 2019) | Low | Low | Low | Low | Low | Low |
| (Vasconcelos et al, 2016) | Low | Low | Low | Low | Low | Low |
| (Dong et al, 2019) | Low | Low | Low | Low | Low | Low |
| (Rezaei et al, 2024) | Low | Low | Low | Low | Low | Low |
| (Chiu et al, 2018) | Low | Unclear | Low | Low | Low | Unclear |
| (He et al, 2024) | Low | Low | Low | Low | Low | Low |
| (Vikberg et al, 2019) | Low | Low | Low | Low | Low | Low |
| (Bellomo et al, 2013) | Low | Low | Low | Low | Low | Low |
| (Seo et al, 2021) | Low | Low | Low | Low | Low | Low |
| (Wei et al, 2022) | Low | Low | Low | Low | Low | Low |
| (Cebrià I Iranzo et al, 2018) | Low | Unclear | Low | Low | Low | Unclear |
| (Hamaguchi et al, 2017) | Low | Low | Low | Low | Low | Low |
| (Huang et al, 2017) | Low | Low | Low | Low | Low | Low |
| (Guo et al, 2024) | Low | Low | Low | Low | Low | Low |
| (Mori & Tokuda, 2022) | Low | Low | Low | Low | Low | Low |
| (Gadelha et al, 2021) | Low | Low | Low | Low | Low | Low |

|  |
| --- |

**S25. Subgroup analysis results**

| **Grip strength** | | | | | | | | | | | | | | | | | | | | | | | | | | | | | | | | | | | | | | | | | | | | | | | | | | | | | | | | | | | | | | | | | | | | | | | | | |  |  |  |  |  |
| --- | --- | --- | --- | --- | --- | --- | --- | --- | --- | --- | --- | --- | --- | --- | --- | --- | --- | --- | --- | --- | --- | --- | --- | --- | --- | --- | --- | --- | --- | --- | --- | --- | --- | --- | --- | --- | --- | --- | --- | --- | --- | --- | --- | --- | --- | --- | --- | --- | --- | --- | --- | --- | --- | --- | --- | --- | --- | --- | --- | --- | --- | --- | --- | --- | --- | --- | --- | --- | --- | --- | --- | --- | --- | --- | --- | --- | --- | --- |
| Subgroup | | | | | | | K | | | | | | | | N | | | | | | | | | | | | | Effect size | | | | | | | | | 95% LCI | | | | | | | 95% UCI | | | | | | | | | | t-value | | | | | pd | | | | | | | pb | | | | | | | |  |  |  |  |  |
| Intensity | | | | | | |  | | | | | | | |  | | | | | | | | | | | | |  | | | | | | | | |  | | | | | | |  | | | | | | | | | |  | | | | |  | | | | | | | 0.27 | | | | | | | |  |  |  |  |  |
| High | | | | | | | 3 | | | | | | | | 81 | | | | | | | | | | | | | 0.3583 | | | | | | | | | -0.1407 | | | | | | | 0.8574 | | | | | | | | | | 1.5086 | | | | | 0.1487 | | | | | | |  | | | | | | | |  |  |  |  |  |
| Moderate | | | | | | | 14 | | | | | | | | 204 | | | | | | | | | | | | | 0.3502 | | | | | | | | | -0.1522 | | | | | | | 0.8525 | | | | | | | | | | 1.4644 | | | | | 0.1603 | | | | | | |  | | | | | | | |  |  |  |  |  |
| Low | | | | | | | 4 | | | | | | | | 68 | | | | | | | | | | | | | 0.7211 | | | | | | | | | 0.4284 | | | | | | | 1.0138 | | | | | | | | | | 5.1758 | | | | | <0.01 | | | | | | |  | | | | | | | |  |  |  |  |  |
| Session | | | | | | |  | | | | | | | |  | | | | | | | | | | | | |  | | | | | | | | |  | | | | | | |  | | | | | | | | | |  | | | | |  | | | | | | | 0.38 | | | | | | | |  |  |  |  |  |
| 2 | | | | | | | 13 | | | | | | | | 169 | | | | | | | | | | | | | 0.4483 | | | | | | | | | 0.1287 | | | | | | | 0.7678 | | | | | | | | | | 2.902 | | | | | <0.01 | | | | | | |  | | | | | | | |  |  |  |  |  |
| 3 | | | | | | | 12 | | | | | | | | 263 | | | | | | | | | | | | | 0.6333 | | | | | | | | | 0.3457 | | | | | | | 0.9208 | | | | | | | | | | 4.5559 | | | | | <0.01 | | | | | | |  | | | | | | | |  |  |  |  |  |
| Individual | | | | | | |  | | | | | | | |  | | | | | | | | | | | | |  | | | | | | | | |  | | | | | | |  | | | | | | | | | |  | | | | |  | | | | | | | 0.81 | | | | | | | |  |  |  |  |  |
| Obesity | | | | | | | 21 | | | | | | | | 703 | | | | | | | | | | | | | 0.4952 | | | | | | | | | -0.0287 | | | | | | | 1.0192 | | | | | | | | | | 1.9552 | | | | | 0.0628 | | | | | | |  | | | | | | | |  |  |  |  |  |
| Non-obesity | | | | | | | 4 | | | | | | | | 140 | | | | | | | | | | | | | 0.5627 | | | | | | | | | 0.3256 | | | | | | | 0.7999 | | | | | | | | | | 4.9086 | | | | | <0.01 | | | | | | |  | | | | | | | |  |  |  |  |  |
| **Muscle strength** | | | | | | | | | | | | | | | | | | | | | | | | | | | | | | | | | | | | | | | | | | | | | | | | | | | | | | | | | | | | | | | | | | | | | | | | | |  |  |  |  |  |
| Subgroup | | | | | | | K | | | | | | | | N | | | | | | | | | | | | | Effect size | | | | | | | | | 95% LCI | | | | | | | 95% UCI | | | | | | | | | | t-value | | | | | pd | | | | | | | pb | | | | | | | |  |  |  |  |  |
| Intensity | | | | | | |  | | | | | | | |  | | | | | | | | | | | | |  | | | | | | | | |  | | | | | | |  | | | | | | | | | |  | | | | |  | | | | | | | 0.14 | | | | | | | |  |  |  |  |  |
| High | | | | | | | 7 | | | | | | | | 81 | | | | | | | | | | | | | 0.6638 | | | | | | | | | 0.1812 | | | | | | | 1.1464 | | | | | | | | | | 1.5086 | | | | | <0.01 | | | | | | |  | | | | | | | |  |  |  |  |  |
| Moderate | | | | | | | 27 | | | | | | | | 222 | | | | | | | | | | | | | 0.3755 | | | | | | | | | -0.1011 | | | | | | | 0.852 | | | | | | | | | | 1.4644 | | | | | 0.11 | | | | | | |  | | | | | | | |  |  |  |  |  |
| Low | | | | | | | 7 | | | | | | | | 75 | | | | | | | | | | | | | 0.9044 | | | | | | | | | 0.6425 | | | | | | | 1.1664 | | | | | | | | | | 5.1758 | | | | | <0.01 | | | | | | |  | | | | | | | |  |  |  |  |  |
| Session | | | | | | |  | | | | | | | |  | | | | | | | | | | | | |  | | | | | | | | |  | | | | | | |  | | | | | | | | | |  | | | | |  | | | | | | | 0.73 | | | | | | | |  |  |  |  |  |
| 2 | | | | | | | 33 | | | | | | | | 194 | | | | | | | | | | | | | 0.6773 | | | | | | | | | 0.3984 | | | | | | | 0.9561 | | | | | | | | | | 4.8856 | | | | | <0.01 | | | | | | |  | | | | | | | |  |  |  |  |  |
| 3 | | | | | | | 16 | | | | | | | | 263 | | | | | | | | | | | | | 0.7499 | | | | | | | | | 0.4313 | | | | | | | 1.0684 | | | | | | | | | | 4.7356 | | | | | <0.01 | | | | | | |  | | | | | | | |  |  |  |  |  |
| Individual | | | | | | |  | | | | | | | |  | | | | | | | | | | | | |  | | | | | | | | |  | | | | | | |  | | | | | | | | | |  | | | | |  | | | | | | | 0.80 | | | | | | | |  |  |  |  |  |
| Obesity | | | | | | | 11 | | | | | | | | 168 | | | | | | | | | | | | | 0.6600 | | | | | | | | | 0.2030 | | | | | | | 1.1170 | | | | | | | | | | 2.9053 | | | | | <0.01 | | | | | | |  | | | | | | | |  |  |  |  |  |
| Non-obesity | | | | | | | 38 | | | | | | | | 724 | | | | | | | | | | | | | 0.7239 | | | | | | | | | 0.4844 | | | | | | | 0.9634 | | | | | | | | | | 6.0801 | | | | | <0.01 | | | | | | |  | | | | | | | |  |  |  |  |  |
| **Lean mass** | | | | | | | | | | | | | | | | | | | | | | | | | | | | | | | | | | | | | | | | | | | | | | | | | | | | | | | | | | | | | | | | | | | | | | | | | | |  |  |  |  |
| Subgroup | | | | | | K | | | | | | | | N | | | | | | | | | | | | Effect size | | | | | | | | | | | 95% LCI | | | | | | | | 95% UCI | | | | | | | | | | t-value | | | | | | pd | | | | | | pb | | | | | | | |  |  |  |  |
| Intensity | | | | | |  | | | | | | | |  | | | | | | | | | | | |  | | | | | | | | | | |  | | | | | | | |  | | | | | | | | | |  | | | | | |  | | | | | | 0.86 | | | | | | | |  |  |  |  |
| High | | | | | | 2 | | | | | | | | 40 | | | | | | | | | | | | 0.2704 | | | | | | | | | | | -0.2604 | | | | | | | | 0.8012 | | | | | | | | | | 1.0436 | | | | | | 0.3056 | | | | | |  | | | | | | | |  |  |  |  |
| Moderate | | | | | | 22 | | | | | | | | 140 | | | | | | | | | | | | 0.0968 | | | | | | | | | | | -0.3108 | | | | | | | | 0.5044 | | | | | | | | | | 0.4864 | | | | | | 0.6305 | | | | | |  | | | | | | | |  |  |  |  |
| Low | | | | | | 7 | | | | | | | | 48 | | | | | | | | | | | | 0.1712 | | | | | | | | | | | -0.0471 | | | | | | | | 0.3895 | | | | | | | | | | 1.6067 | | | | | | 0.1193 | | | | | |  | | | | | | | |  |  |  |  |
| Session | | | | | |  | | | | | | | |  | | | | | | | | | | | |  | | | | | | | | | | |  | | | | | | | |  | | | | | | | | | |  | | | | | |  | | | | | | 0.73 | | | | | | | |  |  |  |  |
| 2 | | | | | | 9 | | | | | | | | 105 | | | | | | | | | | | | 0.2254 | | | | | | | | | | | -0.0882 | | | | | | | | 0.539 | | | | | | | | | | 1.4661 | | | | | | 0.1527 | | | | | |  | | | | | | | |  |  |  |  |
| 3 | | | | | | 24 | | | | | | | | 153 | | | | | | | | | | | | 0.2128 | | | | | | | | | | | -0.0203 | | | | | | | | 0.4459 | | | | | | | | | | 1.8619 | | | | | | 0.0721 | | | | | |  | | | | | | | |  |  |  |  |
| Individual | | | | | |  | | | | | | | |  | | | | | | | | | | | |  | | | | | | | | | | |  | | | | | | | |  | | | | | | | | | |  | | | | | |  | | | | | | 0.72 | | | | | | | |  |  |  |  |
| Obesity | | | | | | 14 | | | | | | | | 321 | | | | | | | | | | | | 0.1756 | | | | | | | | | | | -0.1281 | | | | | | | | 0.4792 | | | | | | | | | | 1.1794 | | | | | | 0.2472 | | | | | |  | | | | | | | |  |  |  |  |
| Non-obesity | | | | | | 19 | | | | | | | | 185 | | | | | | | | | | | | 0.2431 | | | | | | | | | | | 0.0047 | | | | | | | | 0.4816 | | | | | | | | | | 2.0799 | | | | | | 0.0459 | | | | | |  | | | | | | | |  |  |  |  |
| **Skeletal muscle index** | | | | | | | | | | | | | | | | | | | | | | | | | | | | | | | | | | | | | | | | | | | | | | | | | | | | | | | | | | | | | | | | | | | | | | | | | | | |  |  |  |
| Subgroup | | | | | | | | | | | | | | | | | | K | | | | | | | N | | | | | Effect size | | | | | | | | | 95% LCI | | | | | | | | 95% UCI | | | | | | | | | | t-value | | | | | | | | pd | | | | | | pb | | | | |  |  |  |
| Intensity | | | | | | | | | | | | | | | | | |  | | | | | | |  | | | | |  | | | | | | | | |  | | | | | | | |  | | | | | | | | | |  | | | | | | | |  | | | | | | 0.19 | | | | |  |  |  |
| High | | | | | | | | | | | | | | | | | | 3 | | | | | | | 81 | | | | | 0.5636 | | | | | | | | | 0.0922 | | | | | | | | 1.0351 | | | | | | | | | | 1.5086 | | | | | | | | <0.05 | | | | | |  | | | | |  |  |  |
| Moderate | | | | | | | | | | | | | | | | | | 3 | | | | | | | 84 | | | | | 0.1715 | | | | | | | | | -0.2709 | | | | | | | | 0.6139 | | | | | | | | | | 1.4644 | | | | | | | | 0.39 | | | | | |  | | | | |  |  |  |
| Low | | | | | | | | | | | | | | | | | | 5 | | | | | | | 75 | | | | | 0.7187 | | | | | | | | | 0.247 | | | | | | | | 1.1904 | | | | | | | | | | 5.1758 | | | | | | | | <0.01 | | | | | |  | | | | |  |  |  |
| Session | | | | | | | | | | | | | | | | | |  | | | | | | |  | | | | |  | | | | | | | | |  | | | | | | | |  | | | | | | | | | |  | | | | | | | |  | | | | | | 0.43 | | | | |  |  |  |
| 2 | | | | | | | | | | | | | | | | | | 5 | | | | | | | 109 | | | | | 0.4012 | | | | | | | | | -0.0432 | | | | | | | | 0.8456 | | | | | | | | | | 2.0117 | | | | | | | | 0.072 | | | | | |  | | | | |  |  |  |
| 3 | | | | | | | | | | | | | | | | | | 7 | | | | | | | 161 | | | | | 0.6141 | | | | | | | | | 0.2367 | | | | | | | | 0.9914 | | | | | | | | | | 3.6258 | | | | | | | | <0.01 | | | | | |  | | | | |  |  |  |
| Individual | | | | | | | | | | | | | | | | | |  | | | | | | |  | | | | |  | | | | | | | | |  | | | | | | | |  | | | | | | | | | |  | | | | | | | |  | | | | | | 0.33 | | | | |  |  |  |
| Obesity | | | | | | | | | | | | | | | | | | 1 | | | | | | | 64 | | | | | 0.1480 | | | | | | | | | -0.7227 | | | | | | | | 1.0187 | | | | | | | | | | 0.3788 | | | | | | | | 0.7127 | | | | | |  | | | | |  |  |  |
| Non-obesity | | | | | | | | | | | | | | | | | | 11 | | | | | | | 473 | | | | | 0.5692 | | | | | | | | | 0.2706 | | | | | | | | 0.8678 | | | | | | | | | | 4.2476 | | | | | | | | <0.01 | | | | | |  | | | | |  |  |  |
| **Skeletal muscle mass** | | | | | | | | | | | | | | | | | | | | | | | | | | | | | | | | | | | | | | | | | | | | | | | | | | | | | | | | | | | | | | | | | | | | | | | | | | | | |  |  |
| Subgroup | | | | | | | | | | | | | | | | | K | | | | | | | N | | | | | Effect size | | | | | | | | | 95% LCI | | | | | | | | 95% UCI | | | | | | | | | | t-value | | | | | | | pd | | | | | | | | pb | | | | | |  |  |
| Intensity | | | | | | | | | | | | | | | | |  | | | | | | |  | | | | |  | | | | | | | | |  | | | | | | | |  | | | | | | | | | |  | | | | | | |  | | | | | | | | 0.98 | | | | | |  |  |
| Moderate | | | | | | | | | | | | | | | | | 7 | | | | | | | 56 | | | | | 0.1745 | | | | | | | | | -0.1734 | | | | | | | | 0.5225 | | | | | | | | | | 0.8586 | | | | | | | 0.419 | | | | | | | |  | | | | | |  |  |
| Low | | | | | | | | | | | | | | | | | 2 | | | | | | | 53 | | | | | 0.1688 | | | | | | | | | -0.296 | | | | | | | | 0.6336 | | | | | | | | | | 1.186 | | | | | | | 0.2743 | | | | | | | |  | | | | | |  |  |
| Session | | | | | | | | | | | | | | | | |  | | | | | | |  | | | | |  | | | | | | | | |  | | | | | | | |  | | | | | | | | | |  | | | | | | |  | | | | | | | | 0.89 | | | | | |  |  |
| 2 | | | | | | | | | | | | | | | | | 7 | | | | | | | 93 | | | | | 0.142 | | | | | | | | | -0.1188 | | | | | | | | 0.4028 | | | | | | | | | | 1.2314 | | | | | | | 0.2494 | | | | | | | |  | | | | | |  |  |
| 3 | | | | | | | | | | | | | | | | | 4 | | | | | | | 42 | | | | | 0.1731 | | | | | | | | | -0.2813 | | | | | | | | 0.6275 | | | | | | | | | | 0.8616 | | | | | | | 0.4113 | | | | | | | |  | | | | | |  |  |
| Individual | | | | | | | | | | | | | | | | |  | | | | | | |  | | | | |  | | | | | | | | |  | | | | | | | |  | | | | | | | | | |  | | | | | | |  | | | | | | | | 0.99 | | | | | |  |  |
| Obesity | | | | | | | | | | | | | | | | | 3 | | | | | | | 94 | | | | | 0.1502 | | | | | | | | | -0.2568 | | | | | | | | 0.5572 | | | | | | | | | | 0.8347 | | | | | | | 0.4255 | | | | | | | |  | | | | | |  |  |
| Non-obesity | | | | | | | | | | | | | | | | | 8 | | | | | | | 170 | | | | | 0.1495 | | | | | | | | | -0.1226 | | | | | | | | 0.4216 | | | | | | | | | | 1.2426 | | | | | | | 0.2454 | | | | | | | |  | | | | | |  |  |
| **Walking ability** | | | | | | | | | | | | | | | | | | | | | | | | | | | | | | | | | | | | | | | | | | | | | | | | | | | | | | | | | | | | | | | | | | | | | | | | | | | | | |  |
| Subgroup | | | | | | | | K | | | | | | | | N | | | | | | | | | | | Effect size | | | | | | | | | 95% LCI | | | | | | | | | | | | 95% UCI | | | | | | | | | t-value | | | | | | | pd | | | | | | | | | pb | | | | |  |
| Intensity | | | | | | | |  | | | | | | | |  | | | | | | | | | | |  | | | | | | | | |  | | | | | | | | | | | |  | | | | | | | | |  | | | | | | |  | | | | | | | | | 0.52 | | | | |  |
| High | | | | | | | | 4 | | | | | | | | 81 | | | | | | | | | | | 0.1415 | | | | | | | | | -0.6585 | | | | | | | | | | | | 0.9416 | | | | | | | | | 0.3691 | | | | | | | 0.716 | | | | | | | | |  | | | | |  |
| Moderate | | | | | | | | 15 | | | | | | | | 205 | | | | | | | | | | | 0.5795 | | | | | | | | | 0.1331 | | | | | | | | | | | | 1.0259 | | | | | | | | | 2.7082 | | | | | | | <0.05 | | | | | | | | |  | | | | |  |
| Low | | | | | | | | 4 | | | | | | | | 15 | | | | | | | | | | | 0.1411 | | | | | | | | | -0.978 | | | | | | | | | | | | 1.2602 | | | | | | | | | 0.263 | | | | | | | 0.7953 | | | | | | | | |  | | | | |  |
| Session | | | | | | | |  | | | | | | | |  | | | | | | | | | | |  | | | | | | | | |  | | | | | | | | | | | |  | | | | | | | | |  | | | | | | |  | | | | | | | | | 0.11 | | | | |  |
| 2 | | | | | | | | 8 | | | | | | | | 111 | | | | | | | | | | | 0.0562 | | | | | | | | | -0.4730 | | | | | | | | | | | | 0.8456 | | | | | | | | | 0.2182 | | | | | | | 0.8289 | | | | | | | | |  | | | | |  |
| 3 | | | | | | | | 20 | | | | | | | | 239 | | | | | | | | | | | 0.5675 | | | | | | | | | 0.2159 | | | | | | | | | | | | 0.9914 | | | | | | | | | 3.3175 | | | | | | | <0.01 | | | | | | | | |  | | | | |  |
| Individual | | | | | | | |  | | | | | | | |  | | | | | | | | | | |  | | | | | | | | |  | | | | | | | | | | | |  | | | | | | | | |  | | | | | | |  | | | | | | | | | 0.63 | | | | |  |
| Obesity | | | | | | | | 5 | | | | | | | | 130 | | | | | | | | | | | 0.2612 | | | | | | | | | -0.4629 | | | | | | | | | | | | 0.9853 | | | | | | | | | 0.7414 | | | | | | | 0.4651 | | | | | | | | |  | | | | |  |
| Non-obesity | | | | | | | | 23 | | | | | | | | 545 | | | | | | | | | | | 0.4503 | | | | | | | | | 0.0939 | | | | | | | | | | | | 0.8068 | | | | | | | | | 2.5968 | | | | | | | 0.0153 | | | | | | | | |  | | | | |  |
| **Weight** | | | | | | | | | | | | | | | | | | | | | | | | | | | | | | | | | | | | | | | | | | | | | | | | | | | | | | | | | | | | | | | | | | | | | | | | | | | | | |  |
| Subgroup | K | | | | | | | | | | | N | | | | | | | | | | Effect size | | | | | | | | | | | | 95% LCI | | | | | | | | 95% UCI | | | | | | | | | t-value | | | | | | | | pd | | | | | | | | | pb | | | | | | | | | |  |
| Intensity |  | | | | | | | | | | |  | | | | | | | | | |  | | | | | | | | | | | |  | | | | | | | |  | | | | | | | | |  | | | | | | | |  | | | | | | | | | 0.93 | | | | | | | | | |  |
| High | 1 | | | | | | | | | | | 24 | | | | | | | | | | -0.0022 | | | | | | | | | | | | -0.6886 | | | | | | | | 0.6843 | | | | | | | | | -0.0077 | | | | | | | | 0.9941 | | | | | | | | |  | | | | | | | | | |  |
| Moderate | 7 | | | | | | | | | | | 91 | | | | | | | | | | -0.0304 | | | | | | | | | | | | -0.3528 | | | | | | | | 0.292 | | | | | | | | | -0.2308 | | | | | | | | 0.8252 | | | | | | | | |  | | | | | | | | | |  |
| Session |  | | | | | | | | | | |  | | | | | | | | | |  | | | | | | | | | | | |  | | | | | | | |  | | | | | | | | |  | | | | | | | |  | | | | | | | | | 0.11 | | | | | | | | | |  |
| 2 | 8 | | | | | | | | | | | 111 | | | | | | | | | | 0.0562 | | | | | | | | | | | | -0.473 | | | | | | | | 0.5854 | | | | | | | | | 0.2182 | | | | | | | | 0.8289 | | | | | | | | |  | | | | | | | | | |  |
| 3 | 20 | | | | | | | | | | | 239 | | | | | | | | | | 0.5675 | | | | | | | | | | | | 0.2159 | | | | | | | | 0.9191 | | | | | | | | | 3.3175 | | | | | | | | <0.01 | | | | | | | | |  | | | | | | | | | |  |
| Individual |  | | | | | | | | | | |  | | | | | | | | | |  | | | | | | | | | | | |  | | | | | | | |  | | | | | | | | |  | | | | | | | |  | | | | | | | | | 0.86 | | | | | | | | | |  |
| Obesity | 2 | | | | | | | | | | | 30 | | | | | | | | | | -0.0660 | | | | | | | | | | | | -0.6980 | | | | | | | | 0.5660 | | | | | | | | | -0.2556 | | | | | | | | 0.8068 | | | | | | | | |  | | | | | | | | | |  |
| Non-obesity | 6 | | | | | | | | | | | 190 | | | | | | | | | | -0.0143 | | | | | | | | | | | | -0.3433 | | | | | | | | 0.3147 | | | | | | | | | -0.1061 | | | | | | | | 0.9189 | | | | | | | | |  | | | | | | | | | |  |
| **BFM** | | | | | | | | | | | | | | | | | | | | | | | | | | | | | | | | | | | | | | | | | | | | | | | | | | | | | | | | | | | | | | | | | | | | | | | | | | | | | |  |
| Subgroup | | | K | | | | | | N | | | | | | | | | | | Effect size | | | | | | | | | | | | 95% LCI | | | | | | | | | 95% UCI | | | | | | | | | t-value | | | | | | | | | | pd | | | | | | | | | pb | | | | | | | | |  |
| Intensity | | |  | | | | | |  | | | | | | | | | | |  | | | | | | | | | | | |  | | | | | | | | |  | | | | | | | | |  | | | | | | | | | |  | | | | | | | | | 0.97 | | | | | | | | |  |
| High | | | 2 | | | | | | 60 | | | | | | | | | | | -0.1785 | | | | | | | | | | | | -0.6101 | | | | | | | | | 0.2531 | | | | | | | | | -0.978 | | | | | | | | | | 0.3606 | | | | | | | | |  | | | | | | | | |  |
| Moderate | | | 7 | | | | | | 112 | | | | | | | | | | | -0.1858 | | | | | | | | | | | | -0.4741 | | | | | | | | | 0.1025 | | | | | | | | | -1.5242 | | | | | | | | | | 0.1713 | | | | | | | | |  | | | | | | | | |  |
| Session | | |  | | | | | |  | | | | | | | | | | |  | | | | | | | | | | | |  | | | | | | | | |  | | | | | | | | |  | | | | | | | | | |  | | | | | | | | | 0.72 | | | | | | | | |  |
| 2 | | | 5 | | | | | | 56 | | | | | | | | | | | -0.2225 | | | | | | | | | | | | -0.5184 | | | | | | | | | 0.0734 | | | | | | | | | -1.474 | | | | | | | | | | 0.1405 | | | | | | | | |  | | | | | | | | |  |
| 3 | | | 4 | | | | | | 116 | | | | | | | | | | | -0.1516 | | | | | | | | | | | | -0.4198 | | | | | | | | | 0.1166 | | | | | | | | | -1.1077 | | | | | | | | | | 0.268 | | | | | | | | |  | | | | | | | | |  |
| Individual | | |  | | | | | |  | | | | | | | | | | |  | | | | | | | | | | | |  | | | | | | | | |  | | | | | | | | |  | | | | | | | | | |  | | | | | | | | | 0.80 | | | | | | | | |  |
| Obesity | | | 4 | | | | | | 111 | | | | | | | | | | | -0.2191 | | | | | | | | | | | | -0.6191 | | | | | | | | | 0.1810 | | | | | | | | | 0.2364 | | | | | | | | | | 0.2364 | | | | | | | | |  | | | | | | | | |  |
| Non-obesity | | | 5 | | | | | | 219 | | | | | | | | | | | -0.1637 | | | | | | | | | | | | -0.4631 | | | | | | | | | 0.1358 | | | | | | | | | -1.2924 | | | | | | | | | | 0.2372 | | | | | | | | |  | | | | | | | | |  |
| **BMI** | | | | | | | | | | | | | | | | | | | | | | | | | | | | | | | | | | | | | | | | | | | | | | | | | | | | | | | | | | | | | | | | | | | | | | | | | | | | | |  |
| Subgroup | | | | | K | | | | | | N | | | | | | | | Effect size | | | | | | | | | | | | 95% LCI | | | | | | | | | 95% UCI | | | | | | | | | t-value | | | | | | | | | pd | | | | | | | | | | | pb | | | | | | | | |  |
| Intensity | | | | |  | | | | | |  | | | | | | | |  | | | | | | | | | | | |  | | | | | | | | |  | | | | | | | | |  | | | | | | | | |  | | | | | | | | | | | 0.97 | | | | | | | | |  |
| High | | | | | 1 | | | | | | 24 | | | | | | | | 0.0027 | | | | | | | | | | | | -0.7763 | | | | | | | | | 0.7816 | | | | | | | | | 0.0095 | | | | | | | | | 0.9929 | | | | | | | | | | |  | | | | | | | | |  |
| Moderate | | | | | 5 | | | | | | 74 | | | | | | | | -0.009 | | | | | | | | | | | | -0.4426 | | | | | | | | | 0.4247 | | | | | | | | | -0.0574 | | | | | | | | | 0.957 | | | | | | | | | | |  | | | | | | | | |  |
| Session | | | | |  | | | | | |  | | | | | | | |  | | | | | | | | | | | |  | | | | | | | | |  | | | | | | | | |  | | | | | | | | |  | | | | | | | | | | | 0.76 | | | | | | | | |  |
| 2 | | | | | 3 | | | | | | 39 | | | | | | | | -0.05 | | | | | | | | | | | | -0.515 | | | | | | | | | 0.415 | | | | | | | | | -0.2633 | | | | | | | | | 0.8011 | | | | | | | | | | |  | | | | | | | | |  |
| 3 | | | | | 5 | | | | | | 80 | | | | | | | | 0.0264 | | | | | | | | | | | | -0.3324 | | | | | | | | | 0.3852 | | | | | | | | | 0.18 | | | | | | | | | 0.8631 | | | | | | | | | | |  | | | | | | | | |  |
| Individual | | | | |  | | | | | |  | | | | | | | |  | | | | | | | | | | | |  | | | | | | | | |  | | | | | | | | |  | | | | | | | | |  | | | | | | | | | | | 0.70 | | | | | | | | |  |
| Obesity | | | | | 2 | | | | | | 30 | | | | | | | | -0.0947 | | | | | | | | | | | | -0.7269 | | | | | | | | | 0.5374 | | | | | | | | | -0.3666 | | | | | | | | | 0.7265 | | | | | | | | | | |  | | | | | | | | |  |
| Non-obesity | | | | | 6 | | | | | | 198 | | | | | | | | 0.0213 | | | | | | | | | | | | -0.2967 | | | | | | | | | 0.3393 | | | | | | | | | 0.1639 | | | | | | | | | 0.8752 | | | | | | | | | | |  | | | | | | | | |  |
| **Fat%** | | | | | | | | | | | | | | | | | | | | | | | | | | | | | | | | | | | | | | | | | | | | | | | | | | | | | | | | | | | | | | | | | | | | | | | | | | | | | |  |
| Subgroup | | | | K | | | | | | N | | | | | | | | | | | Effect size | | | | | | | | | | | | 95% LCI | | | | | | | | | 95% UCI | | | | | | | | | | t-value | | | | | | | | | pd | | | | | | | | | pb | | | | | | | |  |
| Intensity | | | |  | | | | | |  | | | | | | | | | | |  | | | | | | | | | | | |  | | | | | | | | |  | | | | | | | | | |  | | | | | | | | |  | | | | | | | | | 0.71 | | | | | | | |  |
| Low | | | | 3 | | | | | | 48 | | | | | | | | | | | -0.1493 | | | | | | | | | | | | -0.5679 | | | | | | | | | 0.2693 | | | | | | | | | | -0.8069 | | | | | | | | | 0.4405 | | | | | | | | |  | | | | | | | |  |
| Moderate | | | | 8 | | | | | | 113 | | | | | | | | | | | -0.2321 | | | | | | | | | | | | -0.4993 | | | | | | | | | 0.035 | | | | | | | | | | -1.9657 | | | | | | | | | 0.0809 | | | | | | | | |  | | | | | | | |  |
| Session | | | |  | | | | | |  | | | | | | | | | | |  | | | | | | | | | | | |  | | | | | | | | |  | | | | | | | | | |  | | | | | | | | |  | | | | | | | | | 0.76 | | | | | | | |  |
| 2 | | | | 3 | | | | | | 48 | | | | | | | | | | | -0.2852 | | | | | | | | | | | | -0.6938 | | | | | | | | | 0.1233 | | | | | | | | | | -1.5793 | | | | | | | | | 0.1487 | | | | | | | | |  | | | | | | | |  |
| 3 | | | | 8 | | | | | | 113 | | | | | | | | | | | -0.1745 | | | | | | | | | | | | -0.4444 | | | | | | | | | 0.0954 | | | | | | | | | | -1.4628 | | | | | | | | | 0.1775 | | | | | | | | |  | | | | | | | |  |
| Individual | | | |  | | | | | |  | | | | | | | | | | |  | | | | | | | | | | | |  | | | | | | | | |  | | | | | | | | | |  | | | | | | | | |  | | | | | | | | | 0.27 | | | | | | | |  |
| Obesity | | | | 7 | | | | | | 231 | | | | | | | | | | | -0.2724 | | | | | | | | | | | | -0.5295 | | | | | | | | | -0.0152 | | | | | | | | | | -2.3960 | | | | | | | | | 0.0402 | | | | | | | | |  | | | | | | | |  |
| Non-obesity | | | | 4 | | | | | | 68 | | | | | | | | | | | 0.0031 | | | | | | | | | | | | -0.4634 | | | | | | | | | 0.4697 | | | | | | | | | | 0.0152 | | | | | | | | | 0.9882 | | | | | | | | |  | | | | | | | |  |
| **Fat mass** | | | | | | | | | | | | | | | | | | | | | | | | | | | | | | | | | | | | | | | | | | | | | | | | | | | | | | | | | | | | | | | | | | | | | | | | | | | | | | |
| Subgroup | | K | | | | | | | | | | | N | | | | | | | | | | Effect size | | | | | | | | | | | | 95% LCI | | | | | | | | 95% UCI | | | | | | | | | | t-value | | | | | | | | | pd | | | | | | | | | | pb | | | | | | |
| Intensity | |  | | | | | | | | | | |  | | | | | | | | | |  | | | | | | | | | | | |  | | | | | | | |  | | | | | | | | | |  | | | | | | | | |  | | | | | | | | | | 0.99 | | | | | | |
| High | | 6 | | | | | | | | | | | 76 | | | | | | | | | | -0.1738 | | | | | | | | | | | | -0.4241 | | | | | | | | 0.0764 | | | | | | | | | | -1.4062 | | | | | | | | | 0.1678 | | | | | | | | | |  | | | | | | |
| Moderate | | 32 | | | | | | | | | | | 232 | | | | | | | | | | -0.1599 | | | | | | | | | | | | -0.5239 | | | | | | | | 0.2253 | | | | | | | | | | -0.8069 | | | | | | | | | 0.4247 | | | | | | | | | |  | | | | | | |
| Low | | 3 | | | | | | | | | | | 48 | | | | | | | | | | -0.1493 | | | | | | | | | | | | -0.5239 | | | | | | | | 0.2253 | | | | | | | | | | -0.8069 | | | | | | | | | 0.4247 | | | | | | | | | |  | | | | | | |
| Session | |  | | | | | | | | | | |  | | | | | | | | | |  | | | | | | | | | | | |  | | | | | | | |  | | | | | | | | | |  | | | | | | | | |  | | | | | | | | | | 0.38 | | | | | | |
| 2 | | 17 | | | | | | | | | | | 103 | | | | | | | | | | -0.2185 | | | | | | | | | | | | -0.3794 | | | | | | | | -0.0577 | | | | | | | | | | -2.6632 | | | | | | | | | <0.01 | | | | | | | | | |  | | | | | | |
| 3 | | 27 | | | | | | | | | | | 183 | | | | | | | | | | -0.1392 | | | | | | | | | | | | -0.2526 | | | | | | | | -0.0258 | | | | | | | | | | -2.4066 | | | | | | | | | 0.0161 | | | | | | | | | |  | | | | | | |
| Individual | |  | | | | | | | | | | |  | | | | | | | | | |  | | | | | | | | | | | |  | | | | | | | |  | | | | | | | | | |  | | | | | | | | |  | | | | | | | | | | 0.22 | | | | | | |
| Obesity | | 19 | | | | | | | | | | | 261 | | | | | | | | | | -0.2375 | | | | | | | | | | | | -0.3888 | | | | | | | | -0.0861 | | | | | | | | | | -3.1666 | | | | | | | | | <0.01 | | | | | | | | | |  | | | | | | |
| Non-obesity | | 25 | | | | | | | | | | | 508 | | | | | | | | | | -0.1181 | | | | | | | | | | | | -0.2411 | | | | | | | | 0.0048 | | | | | | | | | | -1.9388 | | | | | | | | | 0.0593 | | | | | | | | | |  | | | | | | |

**S26 GRADE result.**

| Outcomes | Assessment of evidence quality | | | | | Level of evidence* |
| --- | --- | --- | --- | --- | --- | --- |
|  | Risk of bias | Inconsistency | Indirectness | Imprecision | Others |  |
| Grip strength | Not serious | Not serious | Not serious | Serious | None | Low |
| Muscle strength | Not serious | Not serious | Not serious | Serious | None | Very low |
| Lean mass | Not serious | Not serious | Not serious | Not serious | None | Moderate |
| MQ | Not serious | Not serious | Not serious | Serious | None | Very low |
| Skeletal muscle index | Not serious | Not serious | Not serious | Serious | None | Low |
| Skeletal muscle mass | Not serious | Not serious | Not serious | Not serious | None | Moderate |
| Walking ability | Not serious | Not serious | Not serious | Serious | None | Very low |
| Weight | Not serious | Not serious | Not serious | Not serious | None | Moderate |
| BFM | Not serious | Not serious | Not serious | Not serious | None | Moderate |
| BMI | Not serious | Not serious | Not serious | Not serious | None | Moderate |
| Fat% | Not serious | Not serious | Not serious | Not serious | None | Moderate |
| Fat mass | Not serious | Not serious | Not serious | Not serious | None | Moderate |

**S27 Egger’s Test for Publication Bias in Primary Outcomes**

| Outcomes | k (studies) | Egger’s Intercept (SE) | p‑value |
| --- | --- | --- | --- |
| Grip strength | 25 | -0.3580112 | 0.0406 |
| Muscle strength | 49 | -0.8266843 | <0.01 |
| Lean mass | 33 | 0.946406 | 0.212 |
| MQ | 4 | -5.760194 | 0.0636 |
| Skeletal muscle index | 12 | 1.041721 | 0.426 |
| Skeletal muscle mass | 11 | 0.08125614 | 0.891 |
| Walking ability | 28 | -1.084394 | 0.011 |
| Weight | 8 | -0.09359265 | 0.916 |
| BFM | 9 | -0.003086767 | 0.784 |
| BMI | 8 | -0.3246455 | 0.61 |
| Fat% | 11 | -0.5184062 | 0.562 |
| Fat mass | 44 | -0.14373990 | 0.936 |

# S28. Literature search strategy

## Literature Search Strategy for PubMed

| **Search Component** | **Search Terms** |
| --- | --- |
| Population | “aged”[MeSH] OR “aged”[title/abstract] OR “elderly"[title/abstract] OR “older adults”[title/abstract] OR “seniors”[title/abstract] OR “geriatric”[title/abstract] |
| Condition (Sarcopenia-related terms) | “sarcopenia”[MeSH] OR “sarcopenia"[title/abstract] OR “muscle loss”[title/abstract] OR “muscle atrophy”[title/abstract] OR “muscle weakness”[title/abstract] |
| Intervention (Resistance training-related terms) | “resistance training”[MeSH] OR “resistance exercise*”[title/abstract] OR “strength training”[title/abstract] OR “strength exercise*”[title/abstract] OR “weight training”[title/abstract] OR “weight exercise*”[title/abstract] OR “elastic band”[title/abstract] OR “progressive resistance”[title/abstract] OR “grip strengthener”[title/abstract] OR “1RM"[title/abstract] OR “TRX training”[title/abstract] |

**Literature Search Strategy for Web of Science**

| **Search Component** | **Search Terms** |
| --- | --- |
| Condition (Sarcopenia-related terms) | TS=(“sarcopenia” OR “muscle loss” OR “muscle atrophy” OR “muscle weakness”) |
| Intervention (Resistance training-related terms) | TS=(“resistance training” OR “resistance exercise” OR “strength training” OR “weight training” OR “weight exercise” OR “elastic band” OR “progressive resistance” OR “grip strengthener” OR “1RM” OR “TRX training”) |
| Population (Older adults-related terms) | TS=(“aged” OR “elderly” OR “older adults” OR “seniors” OR “geriatric”) |
| Exclusion (Animal studies) | NOT TS=(“animal*” OR “rat” OR “rats” OR “mouse” OR “mice”) |

**Literature Search Strategy for Cochrane Library**

| **Search Component** | **Search Terms** |
| --- | --- |
| Condition (Sarcopenia-related terms) | (sarcopenia* OR “muscle loss” OR “muscle atrophy” OR “muscle weakness”) |
| Intervention (Resistance training-related terms) | (“resistance training” OR “resistance exercise” OR “strength training” OR “weight training” OR “weight exercise” OR “elastic band” OR “progressive resistance” OR “grip strengthener” OR “1RM” OR “TRX training”) |
| Population (Older adults-related terms) | (aged OR elderly OR “older adult*” OR seniors OR geriatric) |
| Exclusion (Animal studies) | NOT (animal* OR rat OR rats OR mouse OR mice) |

**Literature Search Strategy for Embase**

| **Search Component** | **Search Terms** |
| --- | --- |
| Condition (Sarcopenia-related terms) | (‘sarcopenia’/exp OR ‘sarcopenia’:ab,ti OR ‘muscle loss’:ab,ti OR ‘muscle atrophy’:ab,ti OR ‘muscle weakness’:ab,ti) |
| Intervention (Resistance training-related terms) | (‘resistance training’/exp OR ‘resistance exercise’/exp OR ‘strength training’/exp OR ‘weight training’/exp OR ‘weight exercise’/exp OR ‘elastic band’/exp OR ‘progressive resistance’/exp OR ‘grip strengthener’/exp OR ‘1 RM’/exp OR ‘TRX training’/exp) |
| Population (Older adults-related terms) | (‘aged’/exp OR ‘elderly’:ab,ti OR ‘older adult*’:ab,ti OR ‘seniors’:ab,ti OR ‘geriatric’:ab,ti) |
| Exclusion (Animal studies) | NOT (‘animal’/exp NOT ‘human’/exp) NOT (animal*:ab,ti OR rat:ab,ti OR rats:ab,ti OR mouse:ab,ti OR mice:ab,ti) |

**Literature Search Strategy for Scopus**

| **Search Component** | **Search Terms** |
| --- | --- |
| Condition (Sarcopenia-related terms) | TITLE-ABS-KEY((“sarcopenia” OR “muscle loss” OR “muscle atrophy” OR “muscle weakness”)) |
| Intervention (Resistance training-related terms) | TITLE-ABS-KEY((“resistance training” OR “resistance exercise” OR “strength training” OR “weight training” OR “weight exercise” OR “elastic band” OR “progressive resistance” OR “grip strengthener” OR “1RM”)) |
| Population (Older adults-related terms) | TITLE-ABS-KEY((“aged” OR “elderly” OR “older adults” OR “seniors” OR “geriatric”)) |
| Exclusion (Animal studies) | AND NOT TITLE-ABS-KEY((“animal*” OR “rat” OR “rats” OR “mouse” OR “mice”)) |

**S29. Adherence, Dropout Rates, and Safety Monitoring in Included Resistance Training Trials**

| **Study** | **RT Modality** | **Adherence(%)** | **Dropout Rate(%)** | **Reported Adverse Events** | **Notes(on safety monitoring)** |
| --- | --- | --- | --- | --- | --- |
| (Gadelha et al, 2021) | Pre-dialysis resistance training (elastic band, dumbbells, weighted cuffs, bodyweight) | 79.2 | 20.8 | None | Supervised by experienced professionals; adjusted via OMNI RPE scale; prioritized unilateral upper-limb exercises to protect arteriovenous fistula |
| (Liao, Tsauo et al. 2017) | Elastic band resistance training (Theraband) | 97.6 | 8.7 | None | Supervised by licensed senior physical therapists; intensity adjusted via 15-point Borg RPE scale; progressive load advancement (band color or additional sets) |
| (Liao, Tsauo et al. 2018) | Elastic band resistance training (Theraband) | 97.6 | 12.5 | None | Supervised by licensed senior physical therapists; intensity adjusted via Borg RPE scale (13-grade = moderate intensity); progressive load advancement (band color or additional sets) |
| (Flor-Rufino et al, 2023) | High-intensity resistance training (HIRT, ≥70% 1RM) | 95 | 25.5 | 3 cases of muscle discomfort | Supervised by senior researchers(physical education graduate + physical therapist);real-time posture/breathing/technique correction; load adaptation for discomfort; daily attendance recording |
| (Mori & Tokuda, 2022) | Resistance training (elastic band + bodyweight, 50-70% 1RM) | 72.8 | 13.6 | None | Standardized load progression; 2-3 sets per exercise; session duration 30-40 minutes; nutrition controlled (≥30 kcal/kg IBW/day + ≥1.2g/kg IBW/day protein); daily training/supplementation recording |
| (Guo et al, 2024) | Strength training (elastic band + equipment, 40%-85% 1RM) + tai chi hybrid program | 87.7 | 12.3 | None | Progressive load progression (3 cycles); 2-4 sets per exercise; 90-minute sessions (20min warm-up + core training + 10min cool-down); regular CT/QCT imaging monitoring; adherence tracked via full study completion |
| (Huang et al, 2017) | Progressive elastic band resistance training (Thera-Band, 20% intensity increase per progression) | 100 | 0 | None | Supervised by trained physical therapists; small groups (<6 participants); intensity adjusted via Borg RPE scale (13 points = progression trigger); 55-minute sessions (10min warm-up + 40min training + 5min cool-down) |
| (Huang, Ku et al. 2017) | Resistance training (weight equipment, 60-70% 1RM) + aerobic training + combination training | 64.5 | 35.5 | None | Supervised by qualified professional trainers; progressive load adjustment (every 2 weeks); 60-minute sessions; RPE (0-10 scale) recorded post-session; training follows ACSM guidelines |
| (Chen, Wu et al. 2018) | Kettlebell training (60-70% 1RM) | 100 | 0 | None | Supervised by qualified trainers; progressive load adjustment (every 2 weeks); 60-minute sessions; chair-assisted training to prevent injuries; follows ACSM guidelines |
| (Hamaguchi et al, 2017) | Low-repetition light-load power training (weighted vest, 8 sets×3 reps, progressive load) | 92 | 10.5 | None | Supervised by trained personnel; progressive load adjustment (380-760g increment); 15-s rest between sets; Borg scale (RPE≤13) for intensity monitoring; chair-assisted posture maintenance |
| (Vasconcelos et al, 2016) | Progressive resistance training with high-speed component (open/closed chain exercises, 40%-75% 1RM) | 85 | 8.9 | Mild musculoskeletal pain, low back pain, lower limb cramps | Supervised by trained therapists (≥2 per session); group size ≤6 participants; modified Borg Scale for intensity monitoring; postural corrections for adverse effects; 1RM testing for load progression |
| (Ghasemikaram et al, 2021) | High-intensity resistance training (HIRT, machine-based, single-set high-effort, 5-10 reps, periodized) | 85.7 | 11.6 | None | Supervised by trained personnel; periodized training (5 meso-cycles); 1RM testing for load calibration; standardized assessment protocols; dietary supplementation (protein + vitamin D + calcium) provided |
| (Cebrià I Iranzo et al, 2018) | Peripheral muscle training (dumbbells/ankle/wrist weights, 40-60% maximal isometric strength) + respiratory muscle training (Threshold trainer, 40-60% MIP) | 80 | 54.3 | Mild muscular discomfort, generalized fatigue, nasal discomfort, lip constraint (RMTG) | Supervised by 2 physiotherapists per session; group size 8-10 participants; monthly workload adjustment; Borg CR10 Scale for exertion monitoring; pre-session vital signs (BP, HR, SpO₂) recording |
| (Wei et al, 2022) | Hybrid training (Yi Jin Jing + resistance training: elastic band + equipment, 40-85% 1RM) + resistance training alone | 86.5 | 13.5 | None | Supervised by 2 trainers (Yi Jin Jing) + 2 fitness coaches + 6 nurses; 3 training sessions/week; progressive load adjustment (3 cycles); 90-minute sessions (20min warm-up + core training + 10min cool-down); QCT imaging monitoring |
| (Seo et al, 2021) | Body weight-based + elastic band resistance training (3 sets–5 sets, 6–15 reps, progressive intensity via OMNI scale) | 81.5 | 18.5 | None | Supervised by NSCA-CSCS certified specialists; 3 sessions/week; 60-minute sessions (5min warm-up + 50min training + 5min cool-down); OMNI scale (0–10) for intensity monitoring; progressive overload adjustment |
| (Bellomo et al, 2013) | Resistance training (leg press/extension, 60-85% FMT, 3 sets) + global sensorimotor training + focal vibrational therapy (300Hz) | 90 | 11.1 | None | Supervised training sessions; progressive load adjustment (60-85% maximum theoretical force); standardized warm-up (5-10min cycling) + cool-down; blind outcome assessment; regular posture and movement monitoring |
| (Vikberg et al, 2019) | Body weight-based resistance training (suspension bands + optional weights/vests, 2-4 sets, 10-12 reps, progressive intensity via Borg CR-10 scale) | 91 | 13.9 | Mild shoulder pain, vertigo, knee pain, delayed-onset muscle soreness (DOMS) | Supervised by 2 instructors per session; group size ≤12 participants; Borg CR-10 scale (6-7/10) for intensity monitoring; weekly load adjustment; pre-session warm-up (5-10min) |
| (He et al, 2024) | Self-determined sequence training (strength training + Yi Jin Jing + hybrid training, 3 phases × 8 weeks, progressive intensity) | 79.7 | 20.2 | None | Supervised exercise sessions; 3 sessions/week; 90-minute sessions (20min warm-up + core training + 10min cool-down); QCT imaging monitoring; phase-based exercise adjustment based on participant preference |
| (Chiu et al, 2018) | Chair-based resistance training (2-5 lbs sandbags + grip ball, 3 sets, 4-10 reps) | 80.8 | 8.6 | None | Supervised by professional health trainers; 2 sessions/week; 60-minute sessions (5min warm-up + core training + 5min cool-down); RPE scale (6-20) for intensity monitoring; pre-session vital signs and health history assessment |
| (Rezaei et al, 2024) | TRX suspension training (body weight resistance, 3 sets, 8–15 reps, progressive intensity via mat position/body angle) | 83.3 | 13.0 | None | Supervised training sessions; 3 sessions/week; 60-minute sessions (10min warm-up + 40min core training + 10min cool-down); 2-weekly intensity adjustment; pre/post blood sampling and functional test monitoring |
| (Lichtenberg et al, 2019) | High-intensity resistance training (HI-RT, 70-85% 1RM, single-set training on resistance machines, 3 phases × 8-12 weeks) | 95 | 7.0 | Delayed onset of muscular soreness (DOMS) | Fully supervised by trained staff; 2 sessions/week; 50-minute core training sessions (after conditioning); progressive intensity adjustment (linear periodization); combined with protein (1.5g/kg/d) and vitamin D supplementation; regular health status monitoring |
| (Yamada et al, 2019) | Body-weight resistance exercise (with elastic band assistance) | Exercise: 81.0%; Nutritional supplementation: 98.8% | 19.6 | None | Supervised by physical therapists; no falls or musculoskeletal complications |
| (Lee et al, 2021) | Progressive elastic band resistance training (peRET, 3 sets × 10 reps, 25% resistance increment, color-coded bands) | 100 | 0 | None | Supervised throughout by a senior physiotherapist; Three training sessions per week (55 minutes per session: 10-minute warm-up + 40-minute core training + 5-minute cool-down); Group size ≤6 participants; Intensity assessed using the Borg scale (13-point standard); Resistance progressively adjusted according to individual tolerance. |
| (Chien et al, 2022) | Progressive sandbag resistance training (0.5kg→1kg, 3 sets × 8-15 reps, low-intensity/high-repetition) | 82 | 7.5 | None | Outpatient healthcare staff demonstrate exercise techniques; three weekly home training sessions (30 minutes per session: 5–10 minutes warm-up + core training + 5–10 minutes cool-down); weekly telephone/social media monitoring of adherence; RPE scale (13-point standard) to assess intensity; no safety incidents such as hypoglycaemia or falls. |
| (Dong et al, 2019) | Intradialytic resistance training (progressive intensity: ankle weight 0→5kg/foot + elastic ball, 10×10 cycles, 3-5s hold) | 91.1 | 8.9 | 2 cases of falls | Hemodialysis session integration (3 times/week, 12 weeks); continuous ECG monitoring (vital signs); Borg scale (≤15 points) for intensity control; real-time medical staff supervision; immediate exercise termination for abnormal signs (hypertension/hypotension, dyspnea, etc.) |

**References**

Bellomo, R. G., Iodice, P., Maffulli, N., Maghradze, T., Coco, V., & Saggini, R. (2013). Muscle strength and balance training in sarcopenic elderly: A pilot study with randomized controlled trial. European Journal of Inflammation, 11(1), 193–201. https://doi.org/10.1177/1721727X1301100118

Cebrià I Iranzo, M. À., Balasch-Bernat, M., Tortosa-Chuliá, M. Á., & Balasch-Parisi, S. (2018). Effects of resistance training of peripheral muscles versus respiratory muscles in older adults with sarcopenia who are institutionalized: A randomized controlled trial. Journal of Aging and Physical Activity, 26(4), 637–646. https://doi.org/10.1123/japa.2017-0268

Chen, H., Chung, Y., Chen, Y., Ho, S., & Wu, H. (2017). Effects of different types of exercise on body composition, muscle strength, and IGF‐1 in the elderly with sarcopenic obesity. Journal of the American Geriatrics Society, 65(4), 827–832. https://doi.org/10.1111/jgs.14722

Chen, H.-T., Wu, H.-J., Chen, Y.-J., Ho, S.-Y., & Chung, Y.-C. (2018). Effects of 8-week kettlebell training on body composition, muscle strength, pulmonary function, and chronic low-grade inflammation in elderly women with sarcopenia. Experimental Gerontology, 112, 112–118. https://doi.org/10.1016/j.exger.2018.09.015

Chien, Y.-H., Tsai, C.-J., Wang, D.-C., Chuang, P.-H., & Lin, H.-T. (2022). Effects of 12-week progressive sandbag exercise training on glycemic control and muscle strength in patients with type 2 diabetes mellitus combined with possible sarcopenia. International Journal of Environmental Research and Public Health, 19(22), 15009. https://doi.org/10.3390/ijerph192215009

Chiu, S.-C., Yang, R.-S., Yang, R.-J., & Chang, S.-F. (2018). Effects of resistance training on body composition and functional capacity among sarcopenic obese residents in long-term care facilities: A preliminary study. BMC Geriatrics, 18(1), 21. https://doi.org/10.1186/s12877-018-0714-6

Dong, Z.-J., Zhang, H.-L., & Yin, L.-X. (2019). Effects of intradialytic resistance exercise on systemic inflammation in maintenance hemodialysis patients with sarcopenia: A randomized controlled trial. International Urology and Nephrology, 51(8), 1415–1424. https://doi.org/10.1007/s11255-019-02200-7

Flor-Rufino, C., Barrachina-Igual, J., Pérez-Ros, P., Pablos-Monzó, A., Sanz-Requena, R., & Martínez-Arnau, F. M. (2023). Fat infiltration and muscle hydration improve after high-intensity resistance training in women with sarcopenia. A randomized clinical trial. Maturitas, 168, 29–36. https://doi.org/10.1016/j.maturitas.2022.09.001

Gadelha, A. B., Cesari, M., Corrêa, H. L., Neves, R. V. P., Sousa, C. V., Deus, L. A., Souza, M. K., Reis, A. L., Moraes, M. R., Prestes, J., Simões, H. G., Andrade, R. V., Melo, G. F., & Rosa, T. S. (2021). Effects of pre-dialysis resistance training on sarcopenia, inflammatory profile, and anemia biomarkers in older community-dwelling patients with chronic kidney disease: A randomized controlled trial. International Urology and Nephrology, 53(10), 2137–2147. https://doi.org/10.1007/s11255-021-02799-6

Ghasemikaram, M., Chaudry, O., Nagel, A. M., Uder, M., Jakob, F., Kemmler, W., Kohl, M., & Engelke, K. (2021). Effects of 16 months of high intensity resistance training on thigh muscle fat infiltration in elderly men with osteosarcopenia. GeroScience, 43(2), 607–617. https://doi.org/10.1007/s11357-020-00316-8

Guo, H., Cao, J., He, S., Wei, M., Meng, D., Yu, I., Wang, Z., Chang, X., Yang, G., & Wang, Z. (2024). Quantifying the enhancement of sarcopenic skeletal muscle preservation through a hybrid exercise program: Randomized controlled trial. JMIR Aging, 7, e58175–e58175. https://doi.org/10.2196/58175

Hamaguchi, K., Kurihara, T., Fujimoto, M., Iemitsu, M., Sato, K., Hamaoka, T., & Sanada, K. (2017). The effects of low-repetition and light-load power training on bone mineral density in postmenopausal women with sarcopenia: A pilot study. BMC Geriatrics, 17(1), 102. https://doi.org/10.1186/s12877-017-0490-8

He, S., Wei, M., Meng, D., Wang, Z., Yang, G., & Wang, Z. (2024). Self-determined sequence exercise program for elderly with sarcopenia: A randomized controlled trial with clinical assistance from explainable artificial intelligence. Archives of Gerontology and Geriatrics, 119, 105317. https://doi.org/10.1016/j.archger.2023.105317

Huang, S.-W., Ku, J.-W., Lin, L.-F., Liao, C.-D., Chou, L.-C., & Liou, T.-H. (2017). Body composition influenced by progressive elastic band resistance exercise of sarcopenic obesity elderly women: A pilot randomized controlled trial. European Journal of Physical and Rehabilitation Medicine, 53(4). https://doi.org/10.23736/S1973-9087.17.04443-4

Lee, Y.-H., Lee, P.-H., Lin, L.-F., Liao, C.-D., Liou, T.-H., & Huang, S.-W. (2021). Effects of progressive elastic band resistance exercise for aged osteosarcopenic adiposity women. Experimental Gerontology, 147, 111272. https://doi.org/10.1016/j.exger.2021.111272

Liao, C.-D., Tsauo, J.-Y., Huang, S.-W., Ku, J.-W., Hsiao, D.-J., & Liou, T.-H. (2018). Effects of elastic band exercise on lean mass and physical capacity in older women with sarcopenic obesity: A randomized controlled trial. Scientific Reports, 8(1), 2317. https://doi.org/10.1038/s41598-018-20677-7

Liao, C.-D., Tsauo, J.-Y., Lin, L.-F., Huang, S.-W., Ku, J.-W., Chou, L.-C., & Liou, T.-H. (2017). Effects of elastic resistance exercise on body composition and physical capacity in older women with sarcopenic obesity: A CONSORT-compliant prospective randomized controlled trial. Medicine, 96(23), e7115. https://doi.org/10.1097/MD.0000000000007115

Lichtenberg, T., Von Stengel, S., Sieber, C., & Kemmler, W. (2019). The favorable effects of a high-intensity resistance training on sarcopenia in older community-dwelling men with osteosarcopenia: The randomized controlled FrOST study. Clinical Interventions in Aging, Volume 14, 2173–2186. https://doi.org/10.2147/CIA.S225618

Mori, H., & Tokuda, Y. (2022). De-training effects following leucine-enriched whey protein supplementation and resistance training in older adults with sarcopenia: A randomized controlled trial with 24 weeks of follow-up. The Journal of Nutrition, Health and Aging, 26(11), 994–1002. https://doi.org/10.1007/s12603-022-1853-1

Rezaei, S., Eslami, R., & Tartibian, B. (2024). The effects of TRX suspension training on sarcopenic biomarkers and functional abilities in elderlies with sarcopenia: A controlled clinical trial. BMC Sports Science, Medicine and Rehabilitation, 16(1), 58. https://doi.org/10.1186/s13102-024-00849-x

Seo, M.-W., Jung, S.-W., Kim, S.-W., Lee, J.-M., Jung, H. C., & Song, J.-K. (2021). Effects of 16 weeks of resistance training on muscle quality and muscle growth factors in older adult women with sarcopenia: A randomized controlled trial. International Journal of Environmental Research and Public Health, 18(13), 6762. https://doi.org/10.3390/ijerph18136762

Vasconcelos, K. S. S., Dias, J. M. D., Araújo, M. C., Pinheiro, A. C., Moreira, B. S., & Dias, R. C. (2016). Effects of a progressive resistance exercise program with high-speed component on the physical function of older women with sarcopenic obesity: A randomized controlled trial. Brazilian Journal of Physical Therapy, 20(5), 432–440. https://doi.org/10.1590/bjpt-rbf.2014.0174

Vikberg, S., Sörlén, N., Brandén, L., Johansson, J., Nordström, A., Hult, A., & Nordström, P. (2019). Effects of resistance training on functional strength and muscle mass in 70-year-old individuals with pre-sarcopenia: A randomized controlled trial. Journal of the American Medical Directors Association, 20(1), 28–34. https://doi.org/10.1016/j.jamda.2018.09.011

Wei, M., Meng, D., Guo, H., He, S., Tian, Z., Wang, Z., Yang, G., & Wang, Z. (2022). Hybrid exercise program for sarcopenia in older adults: The effectiveness of explainable artificial intelligence-based clinical assistance in assessing skeletal muscle area. International Journal of Environmental Research and Public Health, 19(16), 9952. https://doi.org/10.3390/ijerph19169952

Yamada, M., Kimura, Y., Ishiyama, D., Nishio, N., Otobe, Y., Tanaka, T., Ohji, S., Koyama, S., Sato, A., Suzuki, M., Ogawa, H., Ichikawa, T., Ito, D., & Arai, H. (2019). Synergistic effect of bodyweight resistance exercise and protein supplementation on skeletal muscle in sarcopenic or dynapenic older adults. Geriatrics & Gerontology International, 19(5), 429–437. https://doi.org/10.1111/ggi.13643

Chen, H.-T., et al. (2018). "Effects of 8-week kettlebell training on body composition, muscle strength, pulmonary function, and chronic low-grade inflammation in elderly women with sarcopenia." Experimental gerontology **112**: 112-118.

Huang, S. W., et al. (2017). "Body composition influenced by progressive elastic band resistance exercise of sarcopenic obesity elderly women: a pilot randomized controlled trial." Eur J Phys Rehabil Med **53**(4): 556-563.

Liao, C.-D., et al. (2018). "Effects of elastic band exercise on lean mass and physical capacity in older women with sarcopenic obesity: A randomized controlled trial." Scientific reports **8**(1): 2317.

Liao, C.-D., et al. (2017). "Effects of protein supplementation combined with resistance exercise on body composition and physical function in older adults: a systematic review and meta-analysis." The American journal of clinical nutrition **106**(4): 1078-1091.
